# Supplementary material for: Propagation of ground penetrating radar waves in Chinese coals
Source: PLoS One. 2020 May 21;15(5):e0233434. doi: 10.1371/journal.pone.0233434 (PMC7241710; doi:10.1371/journal.pone.0233434)
Supplement: S1 File — (PDF) [file pone.0233434.s001.pdf]

Certificate of English Editing

Title

Study of Propagation of Ground Penetrating Radar Waves in Coal for Emergency Rescue of Trapped Miners

Authors

Duo Zhang, Rui Tang Hu Wen, Shixing Fan

Date of Editing

13 April 2020

This document certifies that the paper listed above has been edited to ensure that the language is clear and free of errors. The edit was performed by professional editors at NES(Native English Service). The intent of the author's message was not altered in any way during the editing process. The quality of the edit has been guaranteed, with the assumption that our suggested changes have been accepted and have not been further altered without the knowledge of our editors.

Certificate issued by:

NES Institution

Phone +86-10-8639-2489

Email lunwen@nesediting.com

Xiaoyunli No 8. Chaoyang District, Beijing, P.C. 100025, China
