# Supplementary material for: Propagation of ground penetrating radar waves in Chinese coals
Source: PLoS One. 2020 May 21;15(5):e0233434. doi: 10.1371/journal.pone.0233434 (PMC7241710; doi:10.1371/journal.pone.0233434)
Supplement: Attachment — Submitted filename: Review Comments.pdf [file pone.0233434.s003.pdf]

Review Comments PONE-D-19-29325: “Propagation Law of Ultra-wideband Radar Waves in Coal” by Duo Zhang, Rui Tang, Hu Wen and Shixing Fan

The key contribution of this paper is that it reports the effects of EM wave frequencies on two fundamental parameters or properties: resistivity and dielectric permitivity for different rank coal samples. I am not quite sure one can claim the outcome/results of the lab testing of these parameter can be declared as “Law”. The claim of the Ultra-wideband radar wave is also arguable as the radar wave used is not necessarily ultra-wideband but the frequency was varied from 100 Mhz – 1000 MHz. Otherwise they would not be able to obtain the parameter variations with frequencies. Based on these measurements, it also derived the EM wave attenuation and speed variations with frequencies for different rank coal samples.

The speed is computed by using the simplified version of the wave speed formula (3). I suggest you may also compute with a more accurate formula such as (Zhou and Fullagar, 2001):

The radar phase speed ν is given by:

$$\nu = \frac{\omega}{\beta}, \qquad (2)$$

where $\omega = 2\pi f$ is the angular frequency and where wave number β takes the form:

$$\beta = \left\{ \frac{\omega^2 \mu \varepsilon}{2} \left[\left(1 + \frac{\sigma^2}{\varepsilon^2 \omega^2} \right)^{1/2} + 1 \right] \right\}^{1/2}. \qquad (3)$$

So that it may get different values to the simplified one. Surely the results may do not change much as the frequencies used are relatively high and the approximated version could provide sufficient accuracy.

One of the objectives of the paper is to use GPR for detection of mine trapped personnel after a coal mine disaster. With a frequency ranging from 100 MHz to 1000 MHz, I wonder what kind of penetration capability it will be although the paper claims, in Introduction, the GPR has “strong penetration”. The detection examples in Figures 11 & 12 are simplified too much with no practical use at all. At most, you only illustrate you can estimate the distances of reflection objects without knowing which direction they come from. Such ability is well illustrated in seismic and GPR textbooks. GPR is relatively matured technique. Is there any successful examples such as from controlled experiments in coal mine environment? I guess not. Even if you can receive the reflections from trapped personnel, you need to address the issue such as how to differentiate the human reflections from many other reflections from imhomogeinities of the strata, underground equipment and objects. If you cannot resolve this issue, your GPR measurements have no use to this particular applications.

Based on my above comments, I suggest the author concentrated on the property measurement report rather than try also to address the trapped human detection. Properly understanding the property of coal samples for different frequencies is a good contribution to the literature.

In addition, please improve your English expression of the paper to ensure communicate your finding to your reader effectively. This can be achieved by asking a native English speaker or English Editing services to help you before submit your paper.

Reference

Zhou, B. and Fullagar, P.K., 2001, Delineation of sulphide ore-zones by borehole radar tomography at Hellyer Mine, Australia: Journal of Applied Geophysics, 47, No 3-4, pp.261-269.
