# Supplementary material for: Propagation of ground penetrating radar waves in Chinese coals
Source: PLoS One. 2020 May 21;15(5):e0233434. doi: 10.1371/journal.pone.0233434 (PMC7241710; doi:10.1371/journal.pone.0233434)
Supplement: Attachment — Submitted filename: PONE-D-19-29325_R1_reviewer.pdf [file pone.0233434.s007.pdf]

PLOS ONE

Propagation of Ground Penetrating Radar Waves in Chinese Coals

--Manuscript Draft--

**Manuscript Number:**	PONE-D-19-29325R1
**Article Type:**	Research Article
**Full Title:**	Propagation of Ground Penetrating Radar Waves in Chinese Coals
**Short Title:**	Propagation of Ground Penetrating Radar Waves in Chinese Coals
**Corresponding Author:**	Duo Zhang, Ph.D.<br>Xi'an University of Science and Technology<br>Xi'an, Shaanxi CHINA
**Keywords:**	Coal; Dielectric characterization; UWB; Relative dielectric constant; Specific resistivity; Propagation law
**Abstract:**	The paper presents data from laboratory testing of electrical properties of various coal samples taken from Chinese mines. Test were performed on a commercial Broadband Dielectric Spectrum Test System -Concept-80. It allows to determine the values of dielectric permittivity and specific resistivity of grinded coal samples subjected to the electromagnetic fields in wide frequency spectrum. On the base of laboratory data a set of approximating equations showing the change of electric properties in frequency domain ranging from 100-1000 MHz was obtained. These equations together with known from EM theory formulas for EM waves speed and attenuation were used for modeling and studying the process of radar waves propagation in a coal seam and reflection from the miners entrapped in collapsed tunnels. The modeling concept assumes that a radar transducer with dominating frequency MHz will be lowered through a vertical or inclined rescue borehole to the depth of coal seam with mining opening. It is assumed that in not collapsed part of tunnel only miner is present. Thus on the path of radar wave from transducer to a human we have only one geological interface reflecting the radar signals (coal-air) and one connected with human body. The human reflector can be located in various distances from the tunnel face. This factor was included in analyzed geometrical model. On the base of modeling results the authors of the paper conclude that a reliable human origin reflected radar signal can be measured when a distance between human and transducer do not exceed 5 m.
**Order of Authors:**	Duo Zhang, Ph.D.
	Rui Tang
	Hu Wen
	Shixing Fan
**Response to Reviewers:**	We thank the anonymous reviewers for taking precious time to review our manuscript. We appreciate the reviewers giving us valuable comments that are helpful to improve our manuscript. Here we attempt to reply where and how we revised our manuscript in the light of reviewers' comments. Revised contents of our manuscript and our replies are marked in red font.<br><br>Reviewer #1<br>Thanks for the reviewer's comments. In the revised manuscript, we have modified the title as well as removed the word of "Law". The controversial term UWB to GPR. As shown in Figure 1, we have developed a prototype radar device that can penetrate the coal seam to detect life information. During the research, we tested the dielectric constant of coal and analyzed the propagation of radar waves in the coal. At the initial stage of research, we have assumed some basic physical models. Experts from the China National Key R & D Project team tested our radar prototype at Jining No. 2 Coal Mine. The results show that the radar can penetrate 5m coal seam. We use a combination of the delay band intersection algorithm and the gyroscope at the bottom of the radar to determine the direction of the target. The electrical parameters of strata and human beings are different. Also the directions of reflected waves are different. Therefore, it is possible to distinguish between reflected waves of people and strata. Underground equipments and objects bring enormous interference waves during the

Powered by Editorial Manager® and ProduXion Manager® from Aries Systems Corporation

	detection process. We use the following method to screen effective waves. FIR low-pass filter is made use of eliminating direct waves and some background noises. The adaptive minimum mean square algorithm is used to eliminate periodic interference waves and narrowband waves in order to improve the signal-to-noise ratio.<br>The speed formula was simplified from the accurate formula suggested by the reviewer. We find that the difference between the simplified formula and theaccurate formula was very small. To the certain accuracy, the difference between the results of the two formulas can no longer be seen. For the rigor of the manuscript, we decided to accept the reviewer's opinion that the exact formula was chosen.<br>The article has been edited by academic editing with the native language.<br><br><br>Reviewer #2<br>We thank the reviewer carefully checked on this part. The title has been modified to Thank you very much for your detailed suggestions and comments on the manuscript. In particular, the reviewers' general remarks can be directly used as a new abstract of the paper. We have carefully revised the manuscript based on the comments. For example, Figures 1 and 2 have been removed from the paper. We have checked and corrected the words, descriptions, spelling and grammar in the draft in detail.
**Additional Information:**	
**Question**	**Response**
**Financial Disclosure**<br><br>Enter a financial disclosure statement that describes the sources of funding for the work included in this submission. Review the submission guidelines for detailed requirements. View published research articles from *PLOS ONE* for specific examples.<br><br>This statement is required for submission and **will appear in the published article** if the submission is accepted. Please make sure it is accurate.	Funding: The project was supported by the National Natural Science Foundation of China (grant numbers 51904234 and 51974240) and the Key R & D Program of Shaanxi Provincial (grant number 2017ZDCXL-GY-01-02-03).

**Unfunded studies**<br>Enter: *The author(s) received no specific funding for this work.*<br><br>**Funded studies**<br>Enter a statement with the following details:<br>• Initials of the authors who received each award<br>• Grant numbers awarded to each author<br>• The full name of each funder<br>• URL of each funder website<br>• Did the sponsors or funders play any role in the study design, data collection and analysis, decision to publish, or preparation of the manuscript?<br>• **NO** - Include this sentence at the end of your statement: *The funders had no role in study design, data collection and analysis, decision to publish, or preparation of the manuscript.*<br>• **YES** - Specify the role(s) played.<br><br>* typeset	
**Competing Interests**<br><br>Use the instructions below to enter a competing interest statement for this submission. On behalf of all authors, disclose any competing interests that could be perceived to bias this work—acknowledging all financial support and any other relevant financial or non-financial competing interests.<br><br>This statement **will appear in the published article** if the submission is accepted. Please make sure it is accurate. View published research articles from *PLOS ONE* for specific examples.	The authors have declared that no competing interests exist.

**NO authors have competing interests**<br><br>Enter: *The authors have declared that no competing interests exist.*<br><br>**Authors with competing interests**<br><br>Enter competing interest details beginning with this statement:<br><br>*I have read the journal's policy and the authors of this manuscript have the following competing interests: [insert competing interests here]*<br><br>* typeset	
**Ethics Statement**<br><br>Enter an ethics statement for this submission. This statement is required if the study involved:<br><br>• Human participants<br>• Human specimens or tissue<br>• Vertebrate animals or cephalopods<br>• Vertebrate embryos or tissues<br>• Field research<br><br>Write "N/A" if the submission does not require an ethics statement.<br><br>General guidance is provided below. Consult the submission guidelines for detailed instructions. **Make sure that all information entered here is included in the Methods section of the manuscript.**	N / A

**Format for specific study types**<br><br>**Human Subject Research (involving human participants and/or tissue)**<br>• Give the name of the institutional review board or ethics committee that approved the study<br>• Include the approval number and/or a statement indicating approval of this research<br>• Indicate the form of consent obtained (written/oral) or the reason that consent was not obtained (e.g. the data were analyzed anonymously)<br><br>**Animal Research (involving vertebrate animals, embryos or tissues)**<br>• Provide the name of the Institutional Animal Care and Use Committee (IACUC) or other relevant ethics board that reviewed the study protocol, and indicate whether they approved this research or granted a formal waiver of ethical approval<br>• Include an approval number if one was obtained<br>• If the study involved *non-human primates*, add *additional details* about animal welfare and steps taken to ameliorate suffering<br>• If anesthesia, euthanasia, or any kind of animal sacrifice is part of the study, include briefly which substances and/or methods were applied<br><br>**Field Research**<br><br>Include the following details if this study involves the collection of plant, animal, or other materials from a natural setting:<br>• Field permit number<br>• Name of the institution or relevant body that granted permission	
**Data Availability**<br><br>Authors are required to make all data underlying the findings described fully available, without restriction, and from the time of publication. PLOS allows rare exceptions to address legal and ethical concerns. See the PLOS Data Policy and FAQ for detailed information.	Yes - all data are fully available without restriction

Powered by Editorial Manager® and ProduXion Manager® from Aries Systems Corporation

A Data Availability Statement describing where the data can be found is required at submission. Your answers to this question constitute the Data Availability Statement and **will be published in the article**, if accepted.<br><br>**Important:** Stating 'data available on request from the author' is not sufficient. If your data are only available upon request, select 'No' for the first question and explain your exceptional situation in the text box.<br><br>Do the authors confirm that all data underlying the findings described in their manuscript are fully available without restriction?	
**Describe where the data may be found in full sentences. If you are copying our sample text, replace any instances of XXX with the appropriate details.**<br><br>• If the data are **held or will be held in a public repository**, include URLs, accession numbers or DOIs. If this information will only be available after acceptance, indicate this by ticking the box below. For example: *All XXX files are available from the XXX database (accession number(s) XXX, XXX.).*<br>• If the data are all contained **within the manuscript and/or Supporting Information files**, enter the following: *All relevant data are within the manuscript and its Supporting Information files.*<br>• If neither of these applies but you are able to provide **details of access elsewhere**, with or without limitations, please do so. For example:<br><br>*Data cannot be shared publicly because of [XXX]. Data are available from the XXX Institutional Data Access / Ethics Committee (contact via XXX) for researchers who meet the criteria for access to confidential data.*<br><br>*The data underlying the results presented in the study are available from (include the name of the third party*	All relevant data are within the manuscript and its Supporting Information files

and contact information or URL).<br>• This text is appropriate if the data are owned by a third party and authors do not have permission to share the data.<br><br>* typeset	
Additional data availability information:	Tick here if the URLs/accession numbers/DOIs will be available only after acceptance of the manuscript for publication so that we can ensure their inclusion before publication.

Powered by Editorial Manager® and ProduXion Manager® from Aries Systems Corporation

Manuscript Click here to access/download;Manuscript;Manuscript.doc

Propagation of Ground Penetrating Radar Waves in Chinese Coals

Duo Zhang [a, b, c, *], Rui Tang [a, b], Hu Wen [a, b, c], Shixing Fan [a, b]

[a] School of Safety Science and Engineering, Xi'an University of Science and Technology, Xi'an Shaanxi 710054, China

[b] Key Laboratory of Mine and Disaster Prevention and Control of Ministry of Education, Xi'an University of Science and Technology, Xi'an Shaanxi 710054, China

[c] Postdoctoral Mobile station of Mining Engineering, Xi'an University of Science and Technology, Xi'an Shaanxi 710054, China

[*]Corresponding author: Duo Zhang. E-mail addresses: zhangd@xust.edu.cn

Abstract: The paper presents data from laboratory testing of electrical properties of various coal samples taken from Chinese mines. Test were performed on a commercial Broadband Dielectric Spectrum Test System -Concept-80. It allows to determine the values of dielectric permittivity and specific resistivity of grinded coal samples subjected to the electromagnetic fields in wide frequency spectrum. On the base of laboratory data a set of approximating equations showing the change of electric properties in frequency domain ranging from 100-1000 MHz was obtained. These equations together with known from EM theory formulas for EM waves speed and attenuation were used for modeling and studying the process of radar waves propagation in a coal seam and reflection from the miners entrapped in collapsed tunnels. The modeling concept assumes that a radar transducer with dominating

frequency MHz will be lowered through a vertical or inclined rescue borehole to the depth of coal seam with mining opening. It is assumed that in not collapsed part of tunnel only miner is present. Thus on the path of radar wave from transducer to a human we have only one geological interface reflecting the radar signals (coal-air) and one connected with human body. The human reflector can be located in various distances from the tunnel face. This factor was included in analyzed geometrical model. On the base of modeling results the authors of the paper conclude that a reliable human origin reflected radar signal can be measured when a distance between human and transducer do not exceed 5 m.

Keywords: coal, dielectric permittivity, specific resistivity, radar wave, propagtion.

1. Introduction

In China, coal is still main energy source for the foreseeable future [1, 2]. In addition, it also accounts for a large proportion of the world's primary energy consumption [3-5]. Therefore, safety issues in coal mining processes have always been the focus and hotspot of research. Coal mine emergency rescue is the last barrier to safety and security.

In recent years, with the progress in science, technology, management, and quality of workers, the safety of coal mines has obviously improved. The total number of accidents and death tolls have dropped significantly. However, major accidents in coal mines still occur from time to time. For example, in 2016, there were two particularly significant coal mine accidents and eight major accidents [6]. Coal mine accidents cause serious casualties, as along with economic losses and adverse social

impacts [7]. Obviously, the sooner we knew the location and condition of the people in distress, the greater the possibility that they would be rescued. We should consider the ground rescue method when we encountered the following two situations. First was that the roadway could not be used due to coal or rock fall, high temperature, water, etc. Second was that the lives of rescue workers are threatened uncontrollably. As of now, there have been some successful drilling rescue cases. For example, a water permeable accident occurred in the Kuixi Coal Mine in USA in July 2002. A mine collapse accident occurred in San Jose copper mine in Chile in August 2010 [8]. A mine collapse accident occurred in Yurong gypsum mine in China in December 2015 [9]. Long-distance drilling might be deflected during construction under the current technical conditions (as shown in Fig. 3). Under these circumstances, life information detection technologies, such as video or audio, infrared, and gas, would not be available because they cannot penetrate the coal seam [10]. Among numerous non-contact life information detection technologies, GPR radar was considered to be the most effective technology due to its high resolution, deep penetration, and low power radiation, which is suitable for the life rescue systems [11, 12]. It has been used in life detection and rescue [13]. Many works have been conducted on life detection based on radar [14-18]. Therefore, we believed that GPR radar technology might be the future development direction of life information detection through coal seams. This study showed that the propagation of GPR radar waves in coal, and the related influencing factors have very important scientific and practical significance.

Numerous scholars have conducted extensive research on the electromagnetic

properties of coal and the propagation of electromagnetic waves in coal. Fan ~~and Jia~~ et al studied dielectric properties of coals in the low-terahertz frequency region [19]. Fan ~~and Chang~~ et al studied the dielectric properties of coal in the terahertz frequency region of 100–500 GHz [20]. Qin and Wei researched the relative dielectric constant sweep measurement based on a vector network analyzer [21]. Brach studied the real part of the permittivity of a series of coals as a function of frequency (100 Hz–1.3×10^7 Hz) and temperature (200–400 K), and the results showed that certain parameters are related to the microstructure of the carbonaceous phase [22]. Marland et al. tested the dielectric properties of coal using the microwave cavity method in the laboratory, and qualitatively discussed the effects of temperature, frequency, coal rank, minerals and water content on the coal dielectric properties [23]. Valentina studied the influence of polyethylene terephthalate on the carbonization of bituminous coals and on the modification of their electric and dielectric properties [24] The relationship between interior moisture distribution and coal dielectric properties has been constructed by Wang. The characteristics of permittivity variation with moisture content has also been studied [25]. Peng studied the dielectric characteristics of an Indonesian low-rank coal based on determination of its permittivity from 21 ^{o}C to approximately 1000 ^{o}C at 915 and 2450 MHz in argon [26]. Tao et al., analyzed the effect of sulfur content on the dielectric properties of coal [27]. Xu studied the influence of the crystallite structure on the dielectric properties of coal chars [28]. Liu studied the effect of coal rank on the dielectric properties through structural differences in various coal chars [29]. Emslie ~~et al.~~ put forward the model of a coal

seam waveguide, for the first time, when studying radio wave communication in coal mines [30]. Hill derived the theoretical formula of electromagnetic wave propagation under the layered model and studied the propagation of electromagnetic waves in uneven coal seams [31, 32]. Greenfield used finite difference simulations to analyze the attenuation characteristics of electromagnetic waves in discontinuous coal seams [33]. Luo and Stolarczyk et al., analyzed the effect of electrical properties of coal seams on the attenuation characteristics of electromagnetic waves [34-37]. Zhu et al. reviewed the research progress on the measurement technique of coal dielectric constants and analyzed the dielectric models of several coals [38]. In summary, it is easy to see that there are relatively few investigated on the electrical parameters of different ranks of coal in the frequency range of 100-1000 MHz.

In this paper, we report an experimental study on the dielectric properties of five coal samples of different ranks, with frequencies ranging from 100 MHz to 1000 MHz. The propagation approximating equations of radar waves in coal is constructed, and the propagation process is simulated and solved. The effective distance range of radar waves penetration in coal was given.

2. Experiment and methods

2.1. Dielectric properties measurement

In this study, five kinds of coal samples were used, according to the rank of coal, namely, lignite (1#), in the Dananhu Coal Mine of Xinjiang; long flame coal (2#), in the Tangjiahui Coal Mine of Inner Mongolia; gas coal (3#), in the Baodian Coal Mine of Shandong; lean coal (4#), in the Sangshuping Coal Mine of Shaanxi; anthracite

(5#), in the Huangyanhui Coal Mine of Shanxi. The proximate analysis and elemental analysis results are shown in Table 1. The fresh coal samples that were not subjected to water injection, spraying, and other treatments were sealed and packed with multilayer plastic and nylon bags, and then transported to the laboratory. Each experiment was performed four times to ensure test reproducibility. The samples were prepared as follows. A fresh coal sample was crushed, and pulverized coal whose diameter was less than 0.074 mm was screened out. A certain amount of pulverized coal was weighed into a tableting machine to prepare a circular coal sample piece having a diameter of 1.28 cm and a thickness of 1.0 mm. The conductive silver colloid was evenly coated on both sides of the coal sample, and could be tested after the silver gel was dried. The preparation process of the sample is shown in Fig. 4.

The experimental equipment was a Concept-80 broadband dielectric spectrum test system, which mainly included control software, an E4991A impedance analyzer and test fixture, as shown in Fig. 5.

2.2. The Propagation equations of Radar Waves in Coal

2.2.1. Dielectric properties of tested coal samples

The relative dielectric constant values of the five tested coals are shown in Fig. 6, and resistivity values are shown in Fig. 7.

It can be seen from Fig. 6 that the relative dielectric constants of five different coals followed similar regularities. They decreased first and then increased with increasing test frequency. The minimum values of dielectric constant appeared around 300 MHz, while the largest one were near 1000 MHz. At the same test frequency, lean

coal had the highest relative dielectric constant, followed by lignite, gas coal, long flame coal, and finally anthracite, as shown in Fig. 6(F). The lignite contained a large number of hydrophilic polar functional groups, which could adsorb a large amount of intrinsic moisture. Polar functional groups and moisture had a high relative dielectric constant. Therefore, the dielectric constant of lignite was higher. The order of relative dielectric constants of the other four ranks of coal should be determined by the carbon content in coal. The relative dielectric constant of lean coal was ~~larger~~ than that of lignite because it was influenced by other factors, including porosity [22]. The relative dielectric constants and frequencies were fitted and were found to satisfy the cubic polynomial relationship. The results are shown in Eq. (1), and correlation coefficients are shown in Table 2.

$$\varepsilon = C_3 f^3 + C_2 f^2 + C_1 f + C_0, \left(f \in [100MHz, 1000MHz] \right) \tag{1}$$

Where ε is the relative dielectric permittivity, f is frequency, and C_i is a coefficient (i=0, 1, 2, 3).

It could be seen from Fig. 7 that the resistivity of coal with a different degree of metamorphism had an overall decreasing trend with an increase in test frequency. There was a convex transformation point near 500 MHz, which could divide the trend of resistivity with frequency into two stages. It was found, through fitting, that part Ⅰ conformed to a cubic polynomial and part Ⅱ conformed to a quadratic polynomial. The results were shown in Eq. (2) and Table 3. After 700 MHz, the resistivity of tested coals tended to be flat and the differences between them are small. As the test frequency increased, electrons ~~a~~ in bundled state of the coal structure group might be

converted into freely excited electrons. This caused a significant increase in free radicals (ie, unpaired electrons) of coals, so the resistivity would decrease. Previous studies have shown that anthracite was a resistive absorbing material [39], and the resistivity of mineral impurities in it was higher than that of organic matter [40]. These factors might make the anthracite resistivity relatively ~~large,~~

$$\rho=\begin{cases} C_{13} f^3 + C_{12} f^2 + C_{11} f + C_{10}, & (f \in [100MHz, 500MHz]) \\ C_{22} f^2 + C_{21} f + C_{20}, & (f \in (500MHz, 1000MHz]) \end{cases} \tag{2}$$

2.2.2. The equation of radar wave propagation in coal

To simplify the model, it was assumed that the coal body was a homogeneous medium. Since radar waves area type of electromagnetic wave, their propagation velocity in coal could also be expressed by Eq. (3) [41]. From Eq. (3)[illegible]d Fig. 8, the propagation velocity of radar waves in different coals could be obtained, as shown in Table 4.

$$v=\left[\frac{\varepsilon}{2}\left(\sqrt{1+(1/2\pi f \varepsilon \rho)^2}+1\right)\right]^{-1/2} \tag{3}$$

Where v is the propagation speed of radar waves in coal, in m/s. ε is the specific relative dielectric permittivity of coal.

According to the literature [42], the attenuation coefficient of electromagnetic wave propagation in coal could be characterized by Eq. (4). Combined with the dielectric constant and electrical conductivity of coal, the variation of attenuation coefficient with frequency could be obtained, as shown in Fig. 8

$$a=2\pi f\left[\frac{\varepsilon\mu}{2}\left(\sqrt{1+(1/2\pi f \rho \varepsilon)^2}-1\right)\right]^{1/2} \tag{4}$$

where α is the attenuation coefficient of radar wave in coal, f is the frequency, ε is the relative dielectric permittivity of coal, and ρ is the specific resistivity of coal.

It could be seen from Fig. 8 that the propagation attenuation coefficient of electromagnetic waves in five types of coal followed a similar rule in relation to frequency. It could be divided into two parts, according to the boundary line near 500 MHz. In part Ⅰ, the attenuation coefficient was a cubic function of frequency. It increased first and then decreased with increasing frequency. At the same frequency, the attenuation coefficients of coals, from large to small, were lean coal, gas coal, long flame coal, anthracite, and lignite. In part Ⅱ, the attenuation coefficient was a quadratic function of frequency. It increases with increasing frequency. In ①of part Ⅱ, at the same frequency, the attenuation coefficient from large to small was anthracite, long flame coal (gas coal, lean coal), and brown coal. Among them, the attenuation coefficient of long flame coal, gas coal and lean coal had little difference. In ②of part Ⅱ, at the same frequency, the attenuation coefficient from large to small was anthracite (long flame coal, gas coal), lean coal, and brown coal. Among them, the attenuation coefficient of anthracite, long flame coal, and gas coal were similar.

The attenuation coefficient and frequency were fitted, and results were shown in Eq. (5) and Table 5.

$$a=\begin{cases} C'_{13} f^3 + C'_{12} f^2 + C'_{11} f + C'_{10}, & (f \in [100MHz,500MHz]) \\ C'_{22} f^2 + C'_{21} f + C'_{20}, & (f \in (500MHz,1000MHz]) \end{cases} \quad (5)$$

According to literature [43], reflection and refraction coefficients of radar waves propagating in coal could be characterized by Eqs. (6) and (7).

$$\text{Reflection coefficient:}\quad R_{ij}=\frac{\sqrt{\varepsilon_i}-\sqrt{\varepsilon_j}}{\sqrt{\varepsilon_i}+\sqrt{\varepsilon_j}} \tag{6}$$

$$\text{Refraction coefficient:}\quad T_{ij}=\frac{2\sqrt{\varepsilon_i}}{\sqrt{\varepsilon_i}+\sqrt{\varepsilon_j}} \tag{7}$$

where R_{ij} is the reflection coefficient of radar waves at the interface between medium *i* and medium *j*. T_{ij} is the refraction coefficient of radar waves at the interface between medium *i* and medium *j*. ε_i is the relative dielectric constant of medium *i*. ε_j is the relative dielectric constant of medium *j*.

From eqs. (6) and (7), respectively, the reflection and refraction coefficients on various interfaces (as shown in Fig. 9) during radar wave propagation could be obtained. The reflection and refraction coefficients of the interface were calculated by taking the relative dielectric constant of coal at a frequency of 500 MHz as an example. The results are shown in Table 6. It was not difficult to find that a negative number appears in the reflection coefficient because the radar wave from the side with a small relatively dielectric constant moves to the side with high relatively dielectric constant, indicating that the reflected wave was opposite in phase to the incident wave.

The propagation distance of radar waves in coal was determined by the attenuation coefficient. It could be seen from Eq. (4) that the independent variables determining variable α (attenuation coefficient) are f (frequency), ε (relatively dielectric constant), the ρ (resistivity), i.e., $\alpha = F(f,\varepsilon,\rho)$. As can be seen from equation (1), ε (relatively dielectric constant) is a function of f (frequency), i.e., $f = F^{-1}(\varepsilon)$. From Eq. (5), it is found that f (frequency) is a function of α (attenuation

coefficient), i.e., $\alpha = F^{-1}(f)$. Thus, the propagation attenuation law of radar waves in coal is given by

$$\begin{cases} a = 2\pi f\left[\dfrac{\varepsilon}{2}\left(\sqrt{1+\left(1/2\pi f\rho\varepsilon\right)^{2}}-1\right)\right]^{1/2} \\ a = \begin{cases} C_{13}'f^{3}+C_{12}'f^{2}+C_{11}'f+C_{10}', & (f\in[100MHz,500MHz]) \\ C_{22}'f^{2}+C_{21}'f+C_{20}', & (f\in(500MHz,1.0GHz]) \end{cases} \\ \varepsilon = C_{3}f^{3}+C_{2}f^{2}+C_{1}f+C_{0}, \left(f\in[100MHz,1.0GHz]\right) \\ \rho = \begin{cases} C_{13}f^{3}+C_{12}f^{2}+C_{11}f+C_{10}, & (f\in[100MHz,500MHz]) \\ C_{22}f^{2}+C_{21}f+C_{20}, & \left(f\in(500MHz,1.0GHz]\right) \end{cases} \end{cases} \tag{8}$$

where α is the attenuation coefficient of radar wave propagation in coal. f is the frequency. ε is a relative dielectric constant. ρ is the resistivity. C_{ij}', C_i, $C_{ij}, \left(i, j\in[0,1,2,3]\right)$ are the coefficients of the corresponding function, respectively.

It was necessary to analyze the characteristics of waves to determine whether there was anyone behind the coal when using radar for rescue. The characteristics of waves were determined by laws of refraction and reflection, that is, Eq. (9), consisting of Eqs. (6) and (7).

$$\begin{cases} R_{ij} = \dfrac{\sqrt{\varepsilon_i}-\sqrt{\varepsilon_j}}{\sqrt{\varepsilon_i}+\sqrt{\varepsilon_j}} \\ T_{ij} = \dfrac{2\sqrt{\varepsilon_i}}{\sqrt{\varepsilon_i}+\sqrt{\varepsilon_j}} \end{cases} \tag{9}$$

where ε_i and ε_j are the relative dielectric constants of substance i and j, respectively. R_{ij} and T_{ij} are the reflection and refraction coefficients, respectively.

According to the relationship between distance, speed and time, the distance between the radar and person in distress can be calculated according to the following formula.

$$
\begin{cases}
L = v \cdot t \\
v = \left[\dfrac{\varepsilon}{2} \left(\sqrt{1 + (1/2\pi f \varepsilon \rho)^2} + 1 \right) \right]^{-1/2}
\end{cases}
\tag{10}
$$

where v is the propagation speed of radar waves in coal, in m/s.

The equations formed by the combination of Eqs. (8), (9), and (10) were the control equations for the propagation of radar waves in coal.

$$
\begin{cases}
a = 2\pi f \left[\dfrac{\varepsilon}{2} \left(\sqrt{1 + (1/2\pi f \rho \varepsilon)^2} - 1 \right) \right]^{1/2} \\
a = \begin{cases} C'_{13} f^3 + C'_{12} f^2 + C'_{11} f + C'_{10}, (f \in [100MHz, 500MHz]) \\ C'_{22} f^2 + C'_{21} f + C'_{20}, (f \in (500MHz, 1.0GHz]) \end{cases} \\
\varepsilon = C_3 f^3 + C_2 f^2 + C_1 f + C_0, (f \in [100MHz, 1.0GHz]) \\
\rho = \begin{cases} C_{13} f^3 + C_{12} f^2 + C_{11} f + C_{10}, (f \in [100MHz, 500MHz]) \\ C_{22} f^2 + C_{21} f + C_{20}, (f \in (500MHz, 1.0GHz]) \end{cases} \\
R_{ij} = \dfrac{\sqrt{\varepsilon_i} - \sqrt{\varepsilon_j}}{\sqrt{\varepsilon_i} + \sqrt{\varepsilon_j}} \\
T_{ij} = \dfrac{2\sqrt{\varepsilon_i}}{\sqrt{\varepsilon_i} + \sqrt{\varepsilon_j}} \\
v = \left[\dfrac{\varepsilon}{2} \left(\sqrt{1 + (1/2\pi f \varepsilon \rho)^2} + 1 \right) \right]^{-1/2} \\
L = v \cdot t
\end{cases}
\tag{11}
$$

3. Results and discussion

The equations (11) could be solved using GprMax code. GprMax is a simulation software based on FDTD theory, which was developed by Dr. Antonis Giannopoulos of the University of Edinburgh [44]. The GprMax has been widely used for simulation and modelling of radar waves propagation in such GPR applications as underground pipeline surveys, roadbed inspections, ancient building surveys, military surveys, quality inspections, and hidden grave surveys [45-49]. The simulation calculation process is shown in Fig. 10. The simulated physical model is shown in Fig. 11(I).

As shown in Fig. 11(II), the thickness of a coal body between a trapped person and radar was L_1, the distance between a person and coal wall was L_2, the width of the

model was L_3, the length of model was L_4, and the size of a human body is 1.6 m ×0.4 m. The specific settings of different models are shown in Table 7. Then, the simulated data was post-processed using Matlab, and the result is shown in Fig. 11(Ⅲ).

It was assumed that coal was a semi-infinite continuous space with homogeneous isotropy. The dielectric constant of a human was 50 [50]. The dielectric constant of air was 1, the antenna stepping distance was 0.06 m, and the transmitting and receiving antenna spacing was 0.065 m. The number of measuring lines was 115, the spatial grid step was 0.005 m×0.005 m, the permeability of all media in the model was 1.0, the boundary condition was a perfect matching layer (PML), the excitation function of the radar was Ricker, and the center frequency was 500 MHz. The partial electrical parameters of the model were set as shown in Table 8. Due to the large number of graphs of simulation results, only lignite was taken as an example for analysis here. The results are shown in Fig. 12.

From Fig. 12, we found that the amplitudes of return wave from the interface between the coal pillar and roadway in seven models were 12.51, 5.48, 2.56, 1.23, 0.63, 0.30, and 0.15 mV/m, and the return amplitude of the human body is -20.63. -10.24, -5.30, -2.72, -1.37, -0.69, and -0.35 mV/m. It was not difficult to find that the intensity of amplitude gradually decreased with the increase of L_1 (thickness of the coal pillar). The larger the L_1, the longer the electromagnetic wave travels in coal, and the loss of electromagnetic wave energy also increased, so the intensity of the target return wave amplitude was gradually smaller. By fitting, it was found that the amplitude of the human body wave decreases with the increase of L_1 as an

exponential function (as shown in Fig. 13). The effective propagation distance of radar waves in coal was between 6 and 8 m for transducer with central frequency 500 MHz.

4. Conclusions

Dielectric properties of five different coals in from Chinese deposits were studied using the alternating current impedance method and the Concept-80 broadband dielectric Spectrum test system for electromagnetic waves with frequencies ranging from 100 MHz to 1000 MHz, for the first time, to the best of our knowledge. The propagation process of radar waves in coal was analyzed.

The experimental results showed that the dielectric constants of five different coals followed similar rules. The values of relative dielectric constant of coals decreased first and then increased with frequency. At the same frequency, the order of values of the relative permittivity of coal from high to low was lean coal, lignite, gas coal, long flame coal, and anthracite. The values of specific resistivity of coal decreased considerably with increasing frequency, but there was observed an upward bump around frequency 500 MHz.

The key parameters of radar waves propagating in coal were analyzed. The propagation velocity values of radar waves in different rank coals and the reflection and refraction coefficients values at different types of interfaces were calculated. The attenuation coefficient of radar waves in five kinds of coals followed a similar rule.

With 500 MHz as the dividing point, the relation between attenuation coefficient of radar waves and frequency was divided into two parts. In part Ⅰ, the frequency was

a cubic function of the attenuation coefficient. The attenuation coefficient initially increases to a certain point and then decreases with increasing of frequency values. In part Ⅱ, the frequency was a quadratic function of the attenuation coefficient, and the values of attenuation coefficient increased with frequency increase. A set of equations for the propagation of radar waves in coal were established, based on the above researches. By analyzing the governing equations, it was found that the effective penetration distance of radar wave with dominant frequency 500MHz in coal ranges is between 6 and 8 m.

Acknowledgements

Funding: The project was supported by the National Natural Science Foundation of China (grant numbers 51904234 and 51974240) and the Key R & D Program of Shaanxi Provincial (grant number 2017ZDCXL-GY-01-02-03).

References

1. Guo L, Zhai M, Wang Z, Zhang Y, Dong P. Comprehensive coal quality index for evaluation of coal agglomeration characteristics, Fuel 2018; 231(12): 379-386.

2. Song H, Liu J, Xue F, Cheng F. The application of ultra-fine fly ash in the seal coating for the wall of underground coal mine, Advanced Powder Technology 2016; 27: 1645-1650.

3. Diniz DA, Prasad P, Pagan RJ. Modern environmental improvement pathways for the coal power generation industry in Australia. Process Safety and Environmental Protection 2004; 82: 191-199.

4. Warmuzinski K. Harnessing methane emissions from coal mining. Process Safety and Environmental Protection 2008; 86: 315-320.

5. British Petroleum (BP), Statistical Review of World Energy, 2015. http://www.bp. ucom/statistical review.

6. Wang D. Thermodynamic disaster in coal mine and its characteristics. Journal of China Coal Society 2018; 43(1): 137-142.

7. Wang K, Jiang S, Ma X, Wu Z, Shao H, Zhang W, et al. Information fusion of plume control and personnel escape during the emergency rescue of external-caused fire in a coal mine. Process Safety and Environmental Protection 2016; 103: 46-59.

8. Ken B. Buried alive, but carbide drills at core of Chile miners' rescue. Metal Powder Report 2010; 65(7): 7-10.

9. Yang T, Du B. Construction Technology of Large Diameter rescue borehole in Pingyi gypsum mine disaster of Shandong, Exploration Engineering (Rock & Soil Drilling and Tunneling) 2017, 44(5): 19-23. (In Chinese)

10. Wen H, Zhang D, Zheng X-Z. Research Progress and Trend of Life Detection Technology of Drilling and Rescue in Mine. Safety in Coal Mines 2017, 48(9): 85-88. (In Chinese)

11. Li Z, Li W, Lv H, Zhang Y, Jing X, Wang J. A novel method for respiration-like clutter cancellation in life detection by dual-frequency IR-UWB radar, IEEE Transactions on Microwave Theory and Techniques 2013; 61 (5): 2086-2092.

12. Hu W, Zhao Z, Wang Y, Zhang H, Lin F. Noncontact accurate measurement of cardiopulmonary activity using a compact quadrature Doppler radar sensor, I IEEE Transactions on Biomedical Engineering 2014; 61 (3): 725-735.

13. Chen K. Huang Y, Zhang J, Norman A. Microwave life-detection systems for searching human subjects under earthquake rubble or behind barrier, IEEE Transactions on Biomedical Engineering 2000; 47 (1): 105-114.

14. JalaliBidgoli F, Moghadami S, Ardalan S. A compact portable microwave lifedetection device for finding survivors. IEEE Embedded Systems Letters 2016; 8(1): 10-13.

15. Liang X, Zhang H, Fang G, Ye S, Gulliver T. An improved algorithm for throughwall target detection using ultra-wideband impulse radar. IEEE Access 2017; (99): 22101-22118.

16. Guan S, Rice JA, Li C, Gu C. Automated dc offset calibration strategy for structural health monitoring based on portable cw radar sensor. IEEE Transactions on Instrumentation and Measurement 2014; 63(12): 3111-3118.

17. Wang F, Horng TS, Peng K, Jau J, Li J, Chen C. Single-antenna Doppler radars using self and mutual injection locking for vital sign detection with random body movement cancellation. IEEE Transactinos on Microwave Theory and Techniques 2011; 59 (12): 3577-3587.

18. Liang X, Zhang H, Lyu T, Xu L, Cao C, Gulliver TA. Ultra-wide band impulse radar for life

detection using wavelet packet decomposition. Physical Communication 2018; 29:31-47.

19. Fan W, Jia C-Y, Hu W, Yang C, Liu L, Zhang X, et al. Dielectric properties of coals in the low-terahertz frequency region. Fuel 2015; 162: 294-304.

20. Fan W, Chang T, Liu L, Cui H. Dielectric properties of coal in the terahertz frequency region of 100–500 GHz. Fuel 2017; 188: 246-253.

21. Qin F, Wei G. Measurement with Sweeping Frequency for Complex Permittivity Based on VNA. Journal of Astronautic Metrology and Measurement 2011; 31(3): 27-30.

22. Brach I, Giuntini JC, Zanchetta JC. Real part of the permittivity of coals and their rank. Fuel 1994; 73(5): 738-741.

23. Marland S, Merchant A., Rowson N. Dielectric properties of coal. Fuel 2001; 80: 1839-1849.

24. Valentina VZ. Influence of polyethylene terephthalate on the carbonisation of bituminous coals and on the modification of their electric and dielectric properties. Fuel; 2006, 85:1652-1665.

25. Wang Q, Zhang X, Gu F. Investigation on interior moisture distribution inducing dielectric anisotropy of coals. Fuel processing technology 2005; 89:633-641.

26. Peng Z, Lin X, Li Z, Hwang J, Kim B, Zhang Y, et al. Dielectric characterization of Indonesian low-rank coal for microwave processing. Fuel processing technology 2017; 156: 171-177.

27. Tao X, Tang L, Xie M, He H, Xu N, Feng L, et al. Dielectric properties analysis of sulfur-containing models in coal and energy evaluation of their sulfur-containing bond dissociation in microwave field. Fuel 2016; 181: 1027-1033.

28. Xu L, Liu H, Jin Y, Fan B, Qiao X, Jing B. Structural order and dielectric properties of coal chars. Fuel 2014; 137: 164-171.

29. Liu H, Xu L, Jin Y, Fan B, Qiao X, Yang Y. Effect of coal rank on structure and dielectric

properties of chars. Fuel 2015; 153: 249-256.

30. Emslie AG, Lagace RL. Propagation of low and medium frequency radio waves in a coal seam. Radio Science 1976; (11): 253-261.

31. Hill DA. Radio propagation in a coal seam and the inverse problem. Journal of Research of the National Bureau of Standards 1984; (89): 385-394.

32. Hill DA. Electromagnetic detection of long conductors in tunnels. IEEE Trans 1985; 33(4).

33. Greenfield RJ,Wu ST. Electromagnetic wave propagation in disrupted coal seams. Geophysics 1991; 56(10): 1571-1577.

34. Luo Y, Stolarczyk LG, Peng SD. Demonstration of the advanced radio imaging method (rim): ground truth results for three-dimensional imaging of coal seams. Sme Meeting 2004.

35. Stolarczyk LG, Fry RC. Radio imaging method (RIM) or diagnostic imaging of anomalous geologic structures in coal seam waveguides. Transactions of Society for Mining, Metallurgy, and Exploration, Inc. 1990; (288): 1806-1814.

36. Stolarczyk LG. Electromagnetic seam wave mapping of roof rock conditions across a longwall panel. 18th International Conference on Ground Control in Mining, Aug 3-5.1999.

37. Stolarczyk LG, Peng SS, Luo Y. Imaging ahead of Mining with Radio Imaging Method (RIM-IV) Instrumentation and Three-dimensional Tomography. Software Proceedings of 22nd International Conference on Ground Control in Mining 2003, Morgantown, WV, 136-143.

38. Zhu H, Xin M, Chang M, Liu D. Research progress on measuring technology of coal dielectric constant. Coal Science Technology 2016; 44(9): 6-12.

39. Singclair DC, West AR. Impedance and modulus spectroscopy of semiconducting $BaTiO_3$

showing positive temperature coefficient of resistance. Journal of Applied. Physics 1989; 66(8): 3850-3856.

40. Feng X, Chen J, Li N, Cui H, Liu J. Study on electromagnetic properties of anthracite and soft coal in microwave field. Study on electromagnetic properties of anthracite 2007; 38(5): 405-411. (In Chinses)

41. Zhou, B. and Fullagar, P.K., 2001, Delineation of sulphide ore-zones by borehole radar tomography at Hellyer Mine, Australia: Journal of Applied Geophysics, 47, No 3-4, pp.261-269.

42. Qi C. Ground penetrating radar signal processing in advanced detection in coal seam, Xi'an university of science and technology 2014. (In Chinese)

43. Liu X. Research the propagation rules of middle-high frequency electromagnetic wave in coal medium. China University of Mining and Technology 2012.

44. Giannopoulos A. Modelling ground penetrating radar by GprMax. Construction and Building Materials 2005; 19: 755-762.

45. Núñez-Nieto X, Solla M, Novob A, Lorenzo H. Three-dimensional ground-penetrating radar methodologies for thecharacterization and volumetric reconstruction of underground tunneling. Construction and Building Materials 2014; 71: 551-560.

46. Adam DB, Jamie K, Pringle. Semblance analysis to assess GPR data from a five-year forensic study of simulated clandestine graves. Journal of Applied Geophysics 2016; 125: 17-44.

47. Lopera O, Milisavljevic N. Prediction of the effects of soil and target properties on the antipersonnel landmine detection performance of ground- penetrating radar: A Colombian case study. Journal of Applied Geophysics 2007; 63: 13-23.

48. Shangguan P, Imad L. AQ, Lahouar S. Pattern recognition algorithms for density estimation of asphalt pavement during compaction: a simulation study. Journal of Applied Geophysics 2014; 107: 8-15.

49. Jaw SW, Hashim M. Locational accuracy of underground utility mapping using ground penetrating radar. Tunnelling and Underground Space Technology Incorporating Trenchless Technology Research 2013; 35: 20-29.

50. Dogaru T, Nguyen L, Le C. Computer models of the human body signature for sensing through the wall radar applications. DTIC Document Adelphi MD 2007.

51. Lee JH, Wang W. Characterization of snow cover using ground penetrating radar for vehicle trafficability–Experiments and modeling. Journal of Terramechanics 2009; 46: 189-202.

Table captions

Table 1 Proximate and elemental analysis of coal.

Table 2 Coefficients of fitting equation.

Table 3 Coefficients of fitting equation.

Table 4 Propagation speed of radar waves in coal (m/s).

Table 5 Coefficients of fitting equation.

Table 6 Reflection and refraction coefficients.

Table 7 Size of model (m).

Table 8 Dielectric characterization of Coal.

Table 1 Proximate and elemental analysis of coal.

Coal	Proximate analysis (%)				Elemental analysis (%)					Porosity
	M_{ad}	A_{ad}	V_{ad}	FC_{ad}	C	H	O	N	S	(%)
1#	8.80	36.62	24.68	29.90	38.94	4.01	22.00	1.15	0.27	10.11
2#	5.77	11.93	32.32	49.98	64.86	5.63	19.20	1.73	0.58	13.34
3#	2.33	15.92	35.45	46.30	63.92	4.85	17.40	2.37	0.51	6.16
4#	0.53	24.49	12.95	62.03	62.51	3.76	17.00	1.61	3.41	9.19
5#	1.20	8.84	7.85	82.11	81.82	3.79	4.90	0.85	1.49	16.20

Table 2 Coefficients of fitting equation.

Coals	C_3	C_2	C_1	C_0	R^2（%）
1#	-9.24×10^{-10}	1.84×10^{-6}	-9.14×10^{-4}	3.62	98.98
2#	-5.82×10^{-10}	1.17×10^{-6}	-5.43×10^{-4}	3.07	98.77
3#	-6.34×10^{-10}	1.28×10^{-6}	-5.96×10^{-4}	3.35	99.21
4#	-7.90×10^{-10}	1.52×10^{-6}	-6.58×10^{-4}	3.78	99.40
5#	-6.42×10^{-10}	1.34×10^{-6}	-6.86×10^{-4}	3.05	98.49

Table 3 Coefficients of fitting equation.

Partion	Coefficient	1#	2#	3#	4#	5#
I	C_{13}	-8.00×10^{-5}	-5.45×10^{-5}	-4.96×10^{-5}	-4.08×10^{-5}	-7.48×10^{-5}
	C_{12}	0.10	0.07	0.06	0.05	0.10
	C_{11}	-43.06	-30.19	-27.52	-23.03	-40.16
	C_{10}	6.13×10^{3}	4.45×10^{3}	4.06×10^{3}	3.43×10^{3}	5.78×10^{3}
	R^2	98.40 %	99.25 %	99.25 %	99.24 %	98.95 %
II	C_{22}	1.28×10^{-3}	9.50×10^{-4}	8.74×10^{-4}	7.99×10^{-4}	1.12×10^{-3}
	C_{21}	-2.36	-1.81	-1.66	-1.48	-2.22
	C_{20}	1.21×10^{3}	9.60×10^{3}	8.80×10^{2}	7.86×10^{2}	1.10×10^{3}
	R^2	97.94 %	99.11 %	98.84 %	98.58 %	98.91 %

Table 4 Propagation speed of radar waves in coal (m/s).

Frequency (MHz)	1#	2#	3#	4#	5#
100	15.70×10^{7}	17.23×10^{7}	16.14×10^{7}	15.53×10^{7}	17.32×10^{7}
200	16.01×10^{7}	17.29×10^{7}	16.64×10^{7}	15.57×10^{7}	17.43×10^{7}
300	16.05×10^{7}	17.32×10^{7}	16.90×10^{7}	15.59×10^{7}	17.46×10^{7}
400	16.03×10^{7}	17.29×10^{7}	16.64×10^{7}	15.55×10^{7}	17.46×10^{7}
500	16.01×10^{7}	17.23×10^{7}	16.14×10^{7}	15.53×10^{7}	17.39×10^{7}
600	15.94×10^{7}	17.20×10^{7}	16.64×10^{7}	15.45×10^{7}	17.37×10^{7}
700	15.87×10^{7}	17.12×10^{7}	16.15×10^{7}	15.38×10^{7}	17.29×10^{7}
800	15.81×10^{7}	17.66×10^{7}	16.42×10^{7}	15.32×10^{7}	17.26×10^{7}
900	15.76×10^{7}	17.14×10^{7}	16.29×10^{7}	15.30×10^{7}	17.17×10^{7}
1000	15.74×10^{7}	16.84×10^{7}	16.26×10^{7}	15.26×10^{7}	17.14×10^{7}

Table 5 Coefficients of fitting equation.

Part	Coefficient	1#	2#	3#	4#	5#
I	C'_{13}	-8.31×10^{-14}	-6.70×10^{-14}	-7.01×10^{-14}	-7.58×10^{-14}	-6.91×10^{-14}
	C'_{12}	6.36×10^{-11}	4.95×10^{-11}	5.17×10^{-11}	5.55×10^{-11}	5.21×10^{-11}
	C'_{11}	-1.03×10^{-8}	-6.20×10^{-9}	-6.46×10^{-9}	-6.90×10^{-9}	-7.25×10^{-9}
	C'_{10}	$4.71\times10^{--7}$	1.97×10^{-7}	2.05×10^{-7}	2.25×10^{-7}	2.52×10^{-7}
	R^2	99.36%	99.72%	99.72%	99.71%	99.69%
II	C'_{22}	-3.31×10^{-12}	-1.31×10^{-12}	-2.22×10^{-12}	-4.63×10^{-12}	-6.46×10^{-11}
	C'_{21}	8.44×10^{-9}	6.78×10^{-9}	7.90×10^{-9}	1.04×10^{-8}	6.12×10^{-9}
	C'_{20}	-2.61×10^{-6}	2.04×10^{-6}	-2.34×10^{-6}	-2.94×10^{-6}	-1.97×10^{-6}
	R^2	98.88%	99.52%	99.37%	98.66%	99.58%

Table 6 Reflection and refraction coefficients.

Coal	Type	(1) air→coal	(2) coal→air	(3) air→person	(4) person→air	(5) coal→person	(6) person→coal
1#	R_{ij}	-0.31	0.31	-0.75	0.75	-0.58	0.58
	T_{ij}	0.698	1.31	0.25	1.75	0.42	1.58
2#	R_{ij}	-0.27	0.27	-0.75	0.75	-0.60	0.60
	T_{ij}	0.28	1.27	0.25	1.75	0.40	1.60
3#	R_{ij}	-0.29	0.29	-0.75	0.75	-0.59	0.59
	T_{ij}	0.71	1.29	0.25	1.75	0.41	1.59
4#	R_{ij}	-0.32	0.32	-0.75	0.75	-0.57	0.57
	T_{ij}	0.68	1.32	0.25	1.75	0.43	1.57
5#	R_{ij}	-0.27	0.27	-0.75	0.75	-0.61	0.67
	T_{ij}	0.737	1.27	0.25	1.75	0.39	1.67

Table 7 Size of model (m).

Model	M1	M2	M3	M4	M5	M6	M7
L_1	2	3	4	5	6	7	8
L_2	1.3	1.3	1.3	1.3	1.3	1.3	1.3
L_3	3.3	4.3	5.3	6.3	7.3	8.3	9.3
L_4	10	10	10	10	10	10	10

Table 8 Dielectric characterization of Coal**.**

Coal	1#	2#	3#	4#	5#
ε	3.51	3.03	3.30	3.73	2.99
ρ (Ω·m)	328.95	290.07	267.78	242.41	318.53

Figure Click here to access/download;Figure;Figure captions.doc

Figure captions

Fig. 1. Schematic concept of and application of borehole GPR method for location of miner trapped in underground tunnel in coal mine.

Fig. 2. Flow chart of processing test of coal samples.

Fig. 3. Concept-80 Broadband Dielectric Spectrum Test System.

Fig. 4. Changes of the relative dielectric permittivity with frequency for various coals.

Fig. 5. Variations of various coals resistivity with frequency.

Fig. 6. Changes of attenuation coefficient with frequency.

Fig. 7. Schematic diagram of types of reflection and refraction.

Fig. 8. Simulation calculation flow.

Fig. 9. Geometrical model of rock strata applied in numerical simulation.

Fig. 10. A plot of signal amplitudes versus travel time.

Fig. 11. Relationship (or a plot) between amplitude of a radar signal reflected from a person and a coal seam thickness L_1.

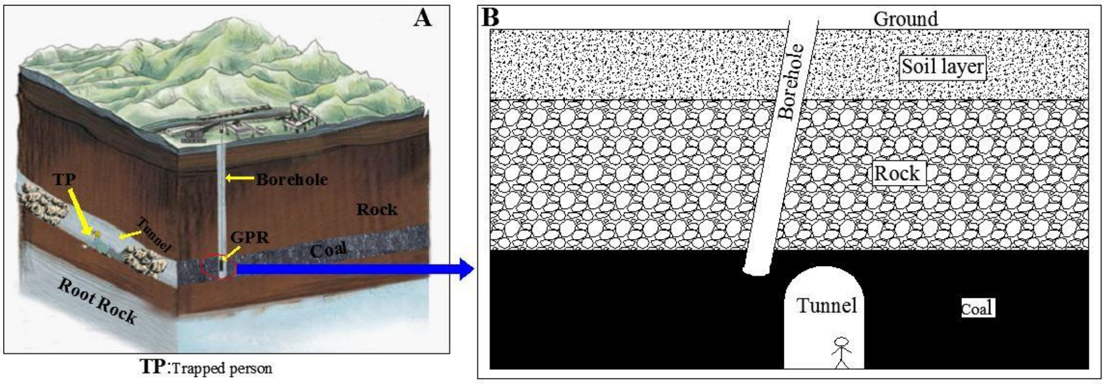

Fig. 1. Schematic concept of and application of borehole GPR method for location of miner trapped in underground tunnel in coal mine.

A is a three-dimensional diagram of rescue drilling during the tunnel collapse; B is a schematic diagram of the drilling offset.

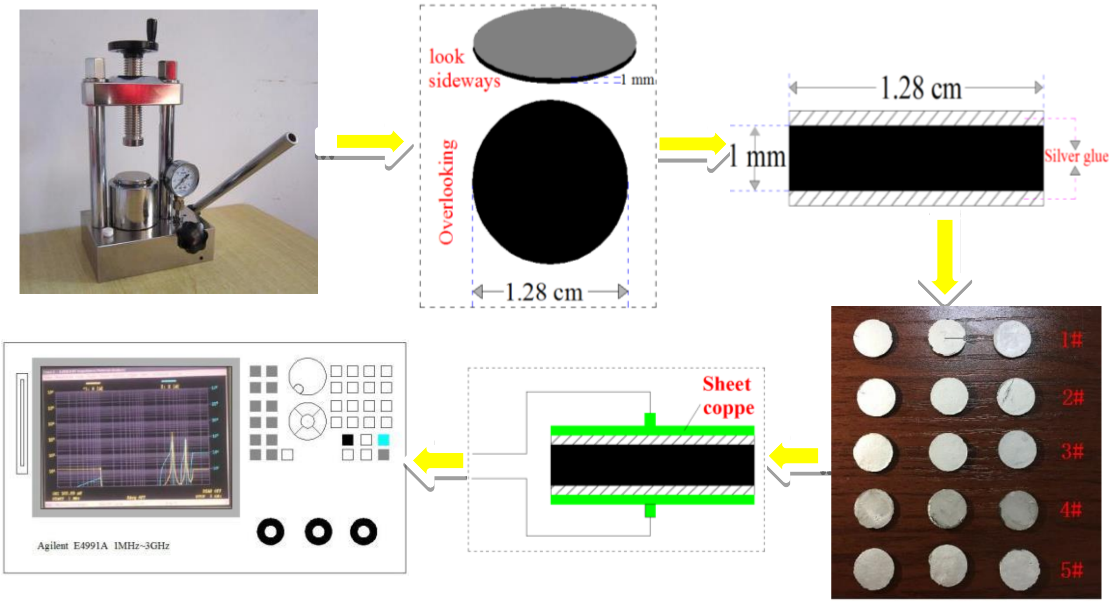

Fig. 2. Flow chart of processing test of coal samples.

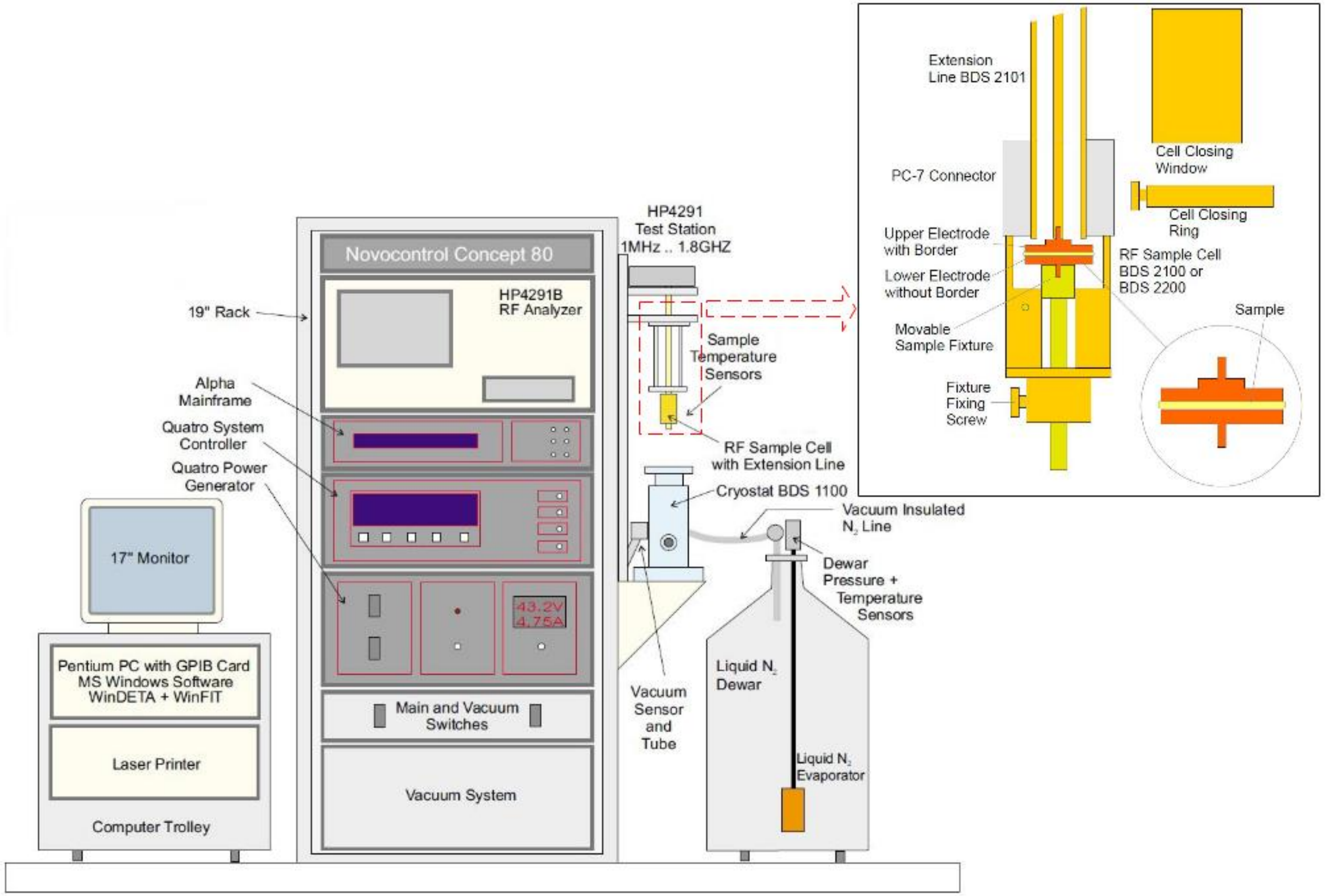

Fig. 3. Concept-80 Broadband Dielectric Spectrum Test System.

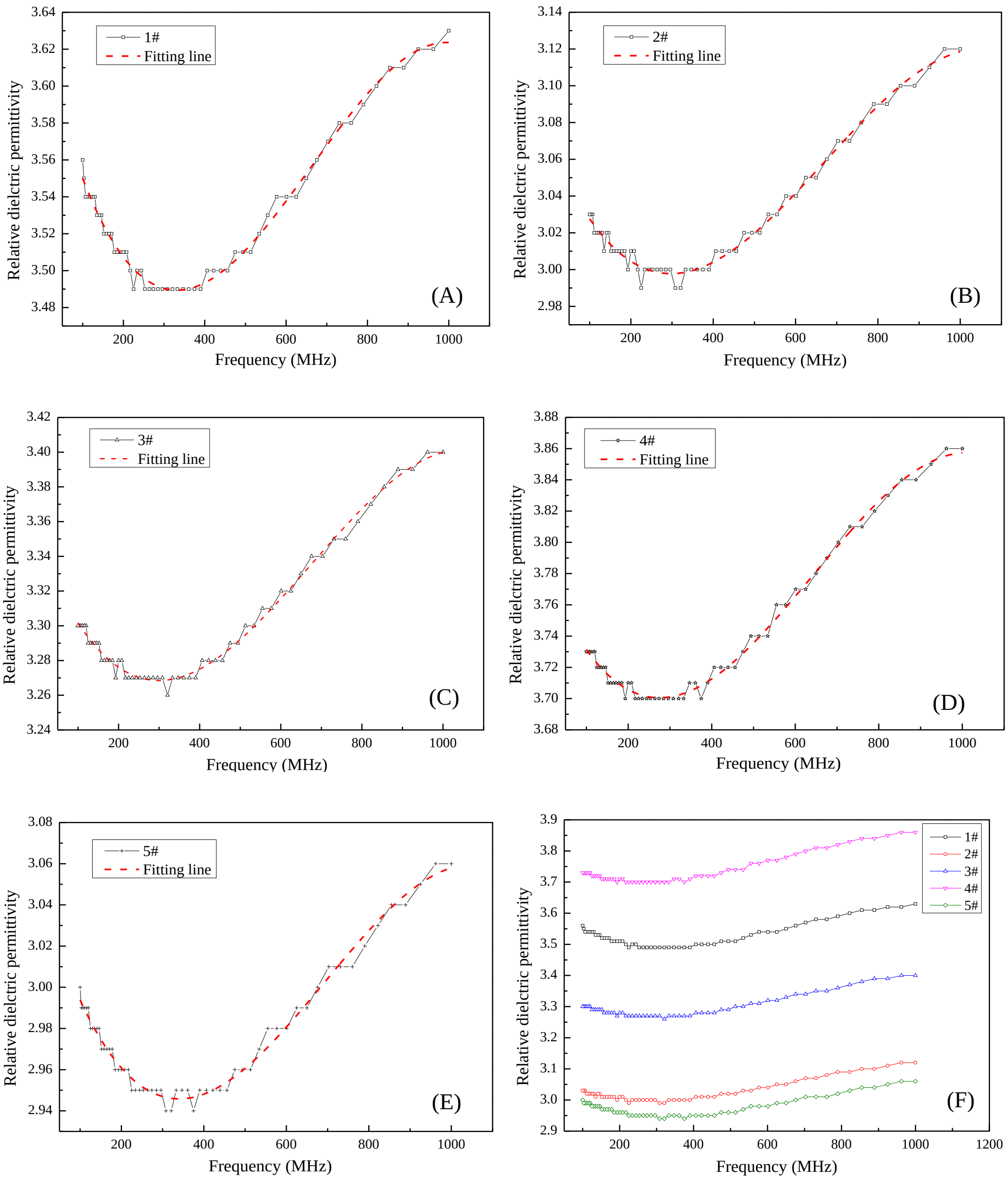

Fig. 4. Changes of the relative dielectric permittivity with frequency for various coals

A: lignite; B: long flame coal; C: gas coal; D: lean coal; E: anthracite;

F: various coals.

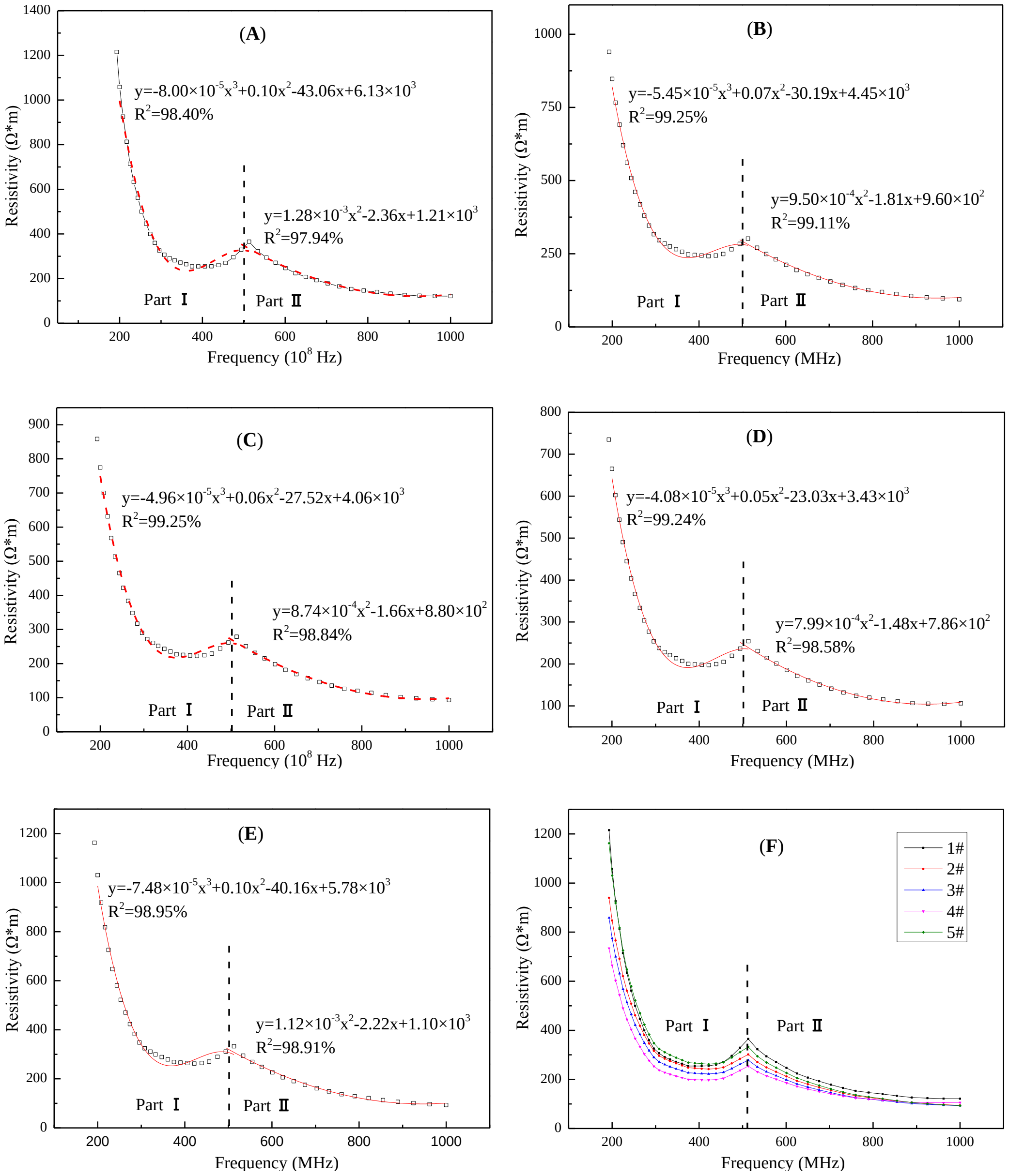

Fig. 5. Variations of various coals resistivity with frequency.

A: lignite; B: long flame coal; C: gas coal; D: lean coal; E: anthracite; F: various coals.

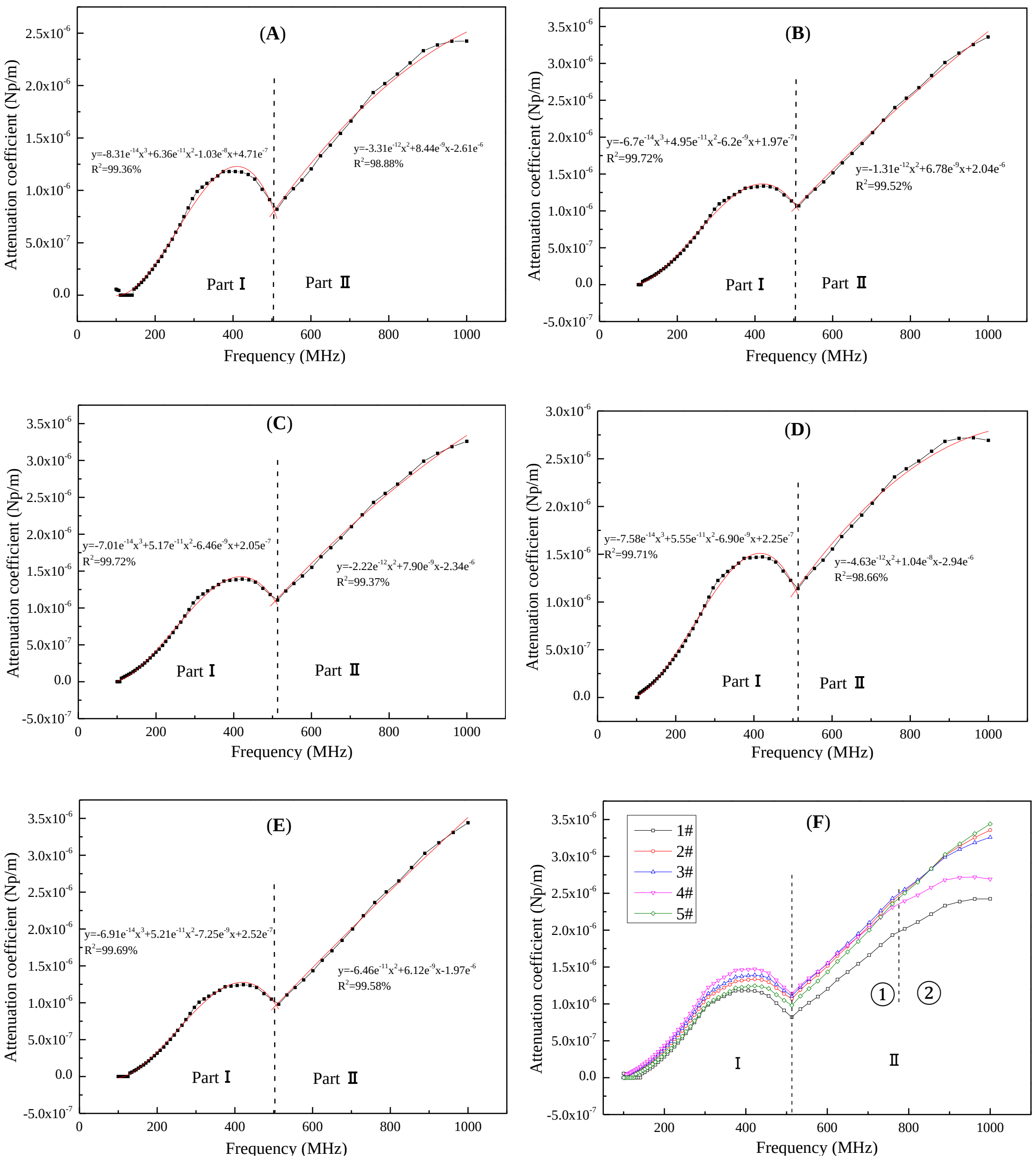

Fig. 6. Changes of attenuation coefficient with frequency.

A: lignite; B: long flame coal; C: gas coal; D: lean coal; E: anthracite; F: various coals.

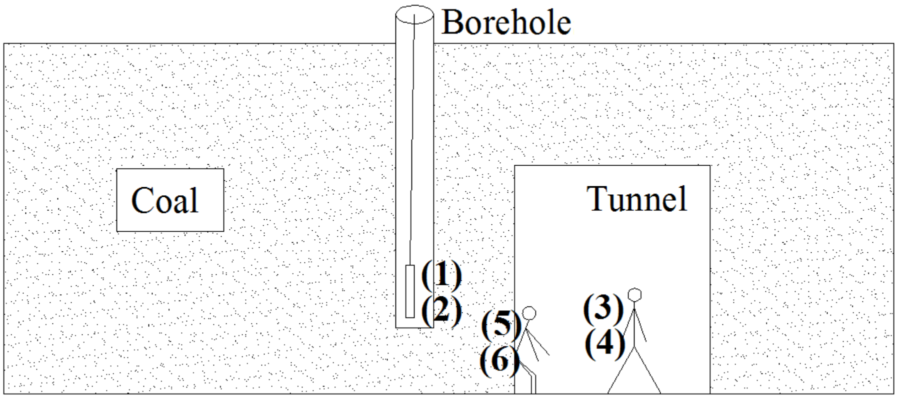

Fig. 7. Schematic diagram of types of reflection and refraction.

Radar waves travel from one object to another. (1): air→coal; (2): coal→air; (3): air→person; (4): person→air; (5): coal→person; (6): person→coal.

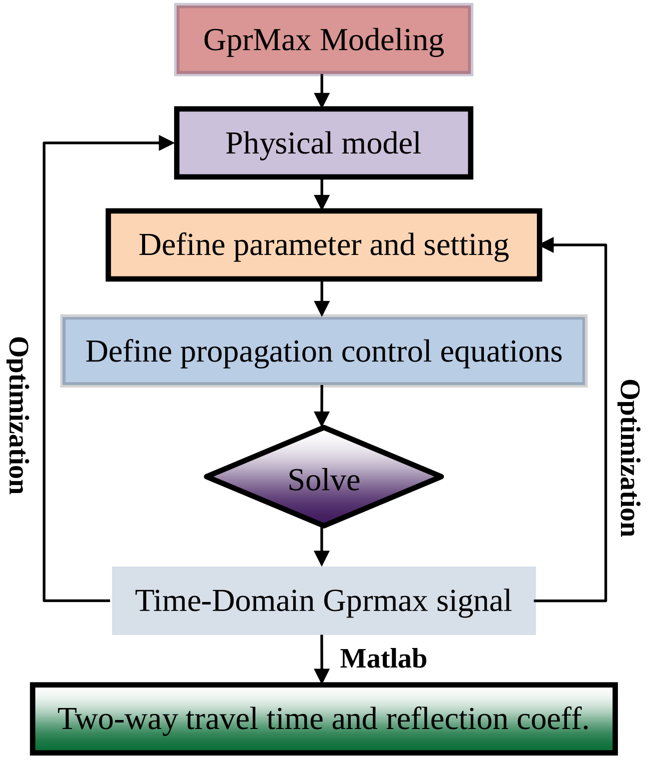

Fig. 8. Simulation calculation flow.

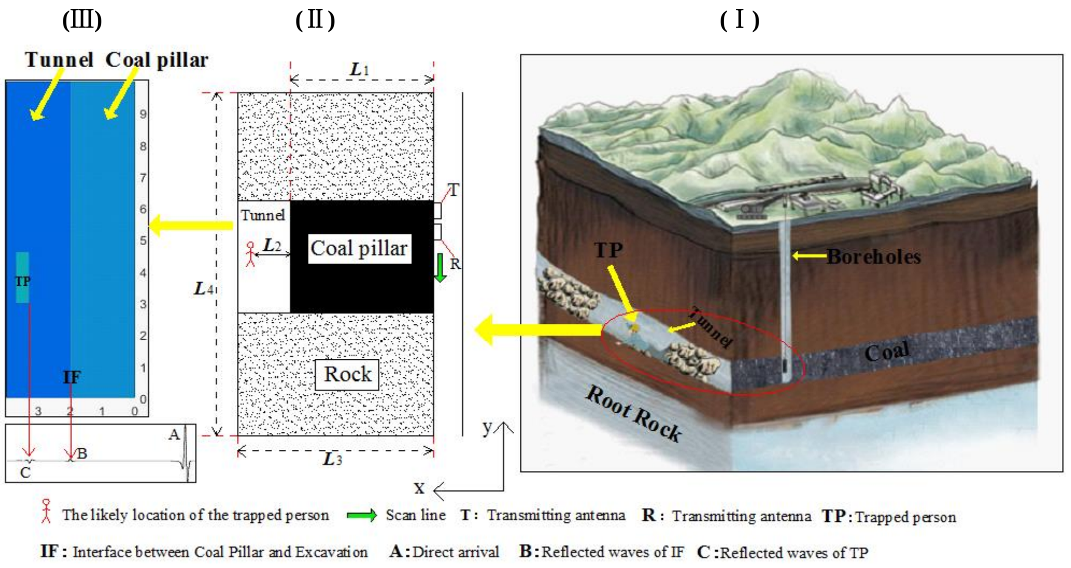

Fig. 9. Geometrical model of rock strata applied in numerical simulation.

Ⅰ: Schematic of illustration of a disaster; Ⅱ: Schematic of simplified of physical model; Ⅲ: Display of physical model in GPR software.

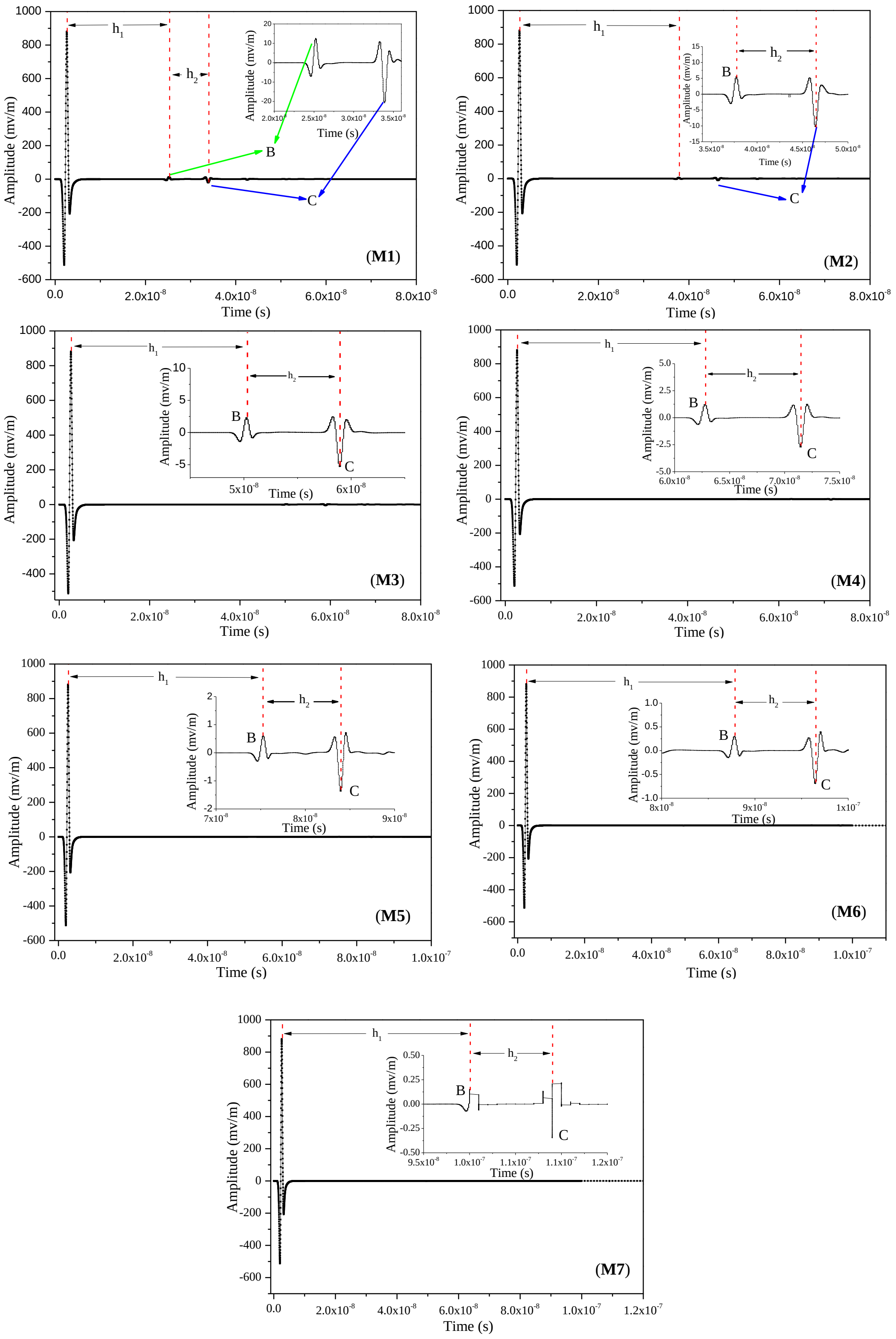

Fig. 10. A plot of signal amplitudes versus travel time.

Mx m are the thickness of the coal body between the radar and the trapped person.

M1: 2 m; M2: 3 m; M3: 4 m; M4: 5 m; M5: 6 m; M6: 7 m; M7: 8 m.

h_i is two-way travel time of radar wave between two objects [52]. The meaning of B and C is given in Fig. 9

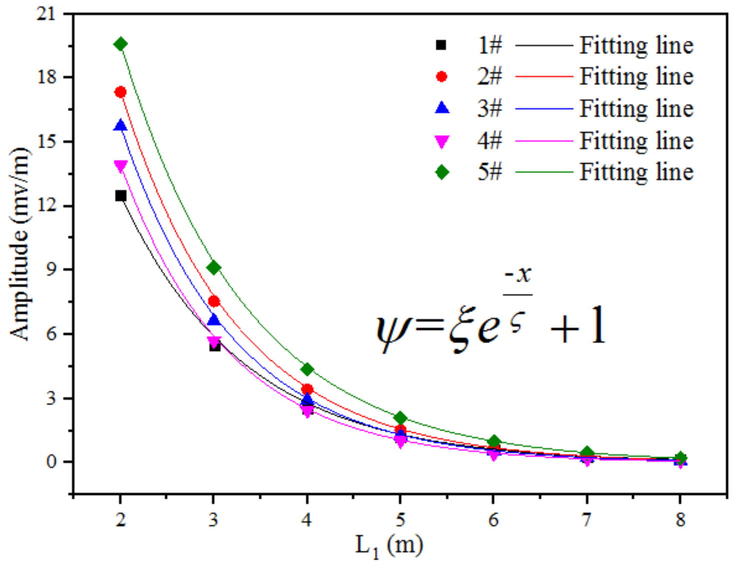

Fig. 11. Relationship (or a plot) between amplitude of a radar signal reflected from a person and a coal seam thickness L1.

Click here to access/download
Supporting Information
Certificate of English Editing.pdf

Propagation of Ground Penetrating Radar Waves in Chinese Coals

Duo Zhang [a, b, c, *], Rui Tang [a, b], Hu Wen [a, b, c], Shixing Fan [a, b]

[a] School of Safety Science and Engineering, Xi'an University of Science and Technology, Xi'an Shaanxi 710054, China

[b] Key Laboratory of Mine and Disaster Prevention and Control of Ministry of Education, Xi'an University of Science and Technology, Xi'an Shaanxi 710054, China

[c] Postdoctoral Mobile station of Mining Engineering, Xi'an University of Science and Technology, Xi'an Shaanxi 710054, China

[*]Corresponding author: Duo Zhang. E-mail addresses: zhangd@xust.edu.cn

Abstract: The paper presents data from laboratory testing of electrical properties of various coal samples taken from Chinese mines. Test were performed on a commercial Broadband Dielectric Spectrum Test System -Concept-80. It allows to determine the values of dielectric permittivity and specific resistivity of grinded coal samples subjected to the electromagnetic fields in wide frequency spectrum. On the base of laboratory data a set of approximating equations showing the change of electric properties in frequency domain ranging from 100-1000 MHz was obtained. These equations together with known from EM theory formulas for EM waves speed and attenuation were used for modeling and studying the process of radar waves propagation in a coal seam and reflection from the miners entrapped in collapsed tunnels. The modeling concept assumes that a radar transducer with dominating

frequency MHz will be lowered through a vertical or inclined rescue borehole to the depth of coal seam with mining opening. It is assumed that in ~~not~~ collapsed part of tunnel only miner is present. Thus on the path of radar wave from transducer to a human we have only one geological interface reflecting the radar signals (coal-air) and one connected with human body. The human reflector can be located in various distances from the tunnel face. This factor was included in analyzed geometrical model. On the base of modeling results the authors of the paper conclude that a reliable human origin reflected radar signal can be measured when a distance between human and transducer do not exceed 5 m.

Keywords: coal, dielectric permittivity, specific resistivity, radar wave, propagtion.

1. Introduction

In China, coal is still main energy source for the foreseeable future [1, 2]. In addition, it also accounts for a large proportion of the world's primary energy consumption [3-5]. Therefore, safety issues in coal mining processes have always been the focus and hotspot of research. Coal mine emergency rescue is the last barrier to safety and security.

In recent years, with the progress in science, technology, management, and quality of workers, the safety of coal mines has obviously improved. The total number of accidents and death tolls have dropped significantly. However, major accidents in coal mines still occur from time to time. For example, in 2016, there were two particularly significant coal mine accidents and eight major accidents [6]. Coal mine accidents cause serious casualties, as along with economic losses and adverse social

impacts [7]. Obviously, the sooner we knew the location and condition of the people in distress, the greater the possibility that they would be rescued. We should consider the ground rescue method when we encountered the following two situations. First was that the roadway could not be used due to coal or rock fall, high temperature, water, etc. Second was that the lives of rescue workers are threatened uncontrollably. As of now, there have been some successful drilling rescue cases. For example, a water permeable accident occurred in the Kuixi Coal Mine in USA in July 2002. A mine collapse accident occurred in San Jose copper mine in Chile in August 2010 [8]. A mine collapse accident occurred in Yurong gypsum mine in China in December 2015 [9]. Long-distance drilling might be deflected during construction under the current technical conditions (as shown in Fig. 3). Under these circumstances, life information detection technologies, such as video or audio, infrared, and gas, would not be available because they cannot penetrate the coal seam [10]. Among numerous non-contact life information detection technologies, GPR radar was considered to be the most effective technology due to its high resolution, deep penetration, and low power radiation, which is suitable for the life rescue systems [11, 12]. It has been used in life detection and rescue [13]. Many works have been conducted on life detection based on radar [14-18]. Therefore, we believed that GPR radar technology might be the future development direction of life information detection through coal seams. This study showed that the propagation of GPR radar waves in coal, and the related influencing factors have very important scientific and practical significance.

Numerous scholars have conducted extensive research on the electromagnetic

properties of coal and the propagation of electromagnetic waves in coal. Fan and Jia et al studied dielectric properties of coals in the low-terahertz frequency region [19]. Fan and Chang et al studied the dielectric properties of coal in the terahertz frequency region of 100–500 GHz [20]. Qin and Wei researched the relative dielectric constant sweep measurement based on a vector network analyzer [21]. Brach studied the real part of the permittivity of a series of coals as a function of frequency (100 Hz–1.3×10^{7} Hz) and temperature (200–400 K), and the results showed that certain parameters are related to the microstructure of the carbonaceous phase [22]. Marland et al. tested the dielectric properties of coal using the microwave cavity method in the laboratory, and qualitatively discussed the effects of temperature, frequency, coal rank, minerals and water content on the coal dielectric properties [23]. Valentina studied the influence of polyethylene terephthalate on the carbonization of bituminous coals and on the modification of their electric and dielectric properties [24]. The relationship between interior moisture distribution and coal dielectric properties has been constructed by Wang. The characteristics of permittivity variation with moisture content has also been studied [25]. Peng studied the dielectric characteristics of an Indonesian low-rank coal based on determination of its permittivity from 21 ^{o}C to approximately 1000 ^{o}C at 915 and 2450 MHz in argon [26]. Tao et al., analyzed the effect of sulfur content on the dielectric properties of coal [27]. Xu studied the influence of the crystallite structure on the dielectric properties of coal chars [28]. Liu studied the effect of coal rank on the dielectric properties through structural differences in various coal chars [29]. Emslie et al. put forward the model of a coal

seam waveguide, for the first time, when studying radio wave communication in coal mines [30]. Hill derived the theoretical formula of electromagnetic wave propagation under the layered model and studied the propagation of electromagnetic waves in uneven coal seams [31, 32]. Greenfield used finite difference simulations to analyze the attenuation characteristics of electromagnetic waves in discontinuous coal seams [33]. Luo and Stolarczyk et al., analyzed the effect of electrical properties of coal seams on the attenuation characteristics of electromagnetic waves [34-37]. Zhu et al. reviewed the research progress on the measurement technique of coal dielectric constants and analyzed the dielectric models of several coals [38]. In summary, it is easy to see that there are relatively few investigated on the electrical parameters of different ranks of coal in the frequency range of 100-1000 MHz.

In this paper, we report an experimental study on the dielectric properties of five coal samples of different ranks, with frequencies ranging from 100 MHz to 1000 MHz. The propagation approximating equations of radar waves in coal is constructed, and the propagation process is simulated and solved. The effective distance range of radar waves penetration in coal was given.

2. Experiment and methods

2.1. Dielectric properties measurement

In this study, five kinds of coal samples were used, according to the rank of coal, namely, lignite (1#), in the Dananhu Coal Mine of Xinjiang; long flame coal (2#), in the Tangjiahui Coal Mine of Inner Mongolia; gas coal (3#), in the Baodian Coal Mine of Shandong; lean coal (4#), in the Sangshuping Coal Mine of Shaanxi; anthracite

(5#), in the Huangyanhui Coal Mine of Shanxi. The proximate analysis and elemental analysis results are shown in Table 1. The fresh coal samples that were not subjected to water injection, spraying, and other treatments were sealed and packed with multilayer plastic and nylon bags, and then transported to the laboratory. Each experiment was performed four times to ensure test reproducibility. The samples were prepared as follows. A fresh coal sample was crushed, and pulverized coal whose diameter was less than 0.074 mm was screened out. A certain amount of pulverized coal was weighed into a tableting machine to prepare a circular coal sample piece having a diameter of 1.28 cm and a thickness of 1.0 mm. The conductive silver colloid was evenly coated on both sides of the coal sample, and could be tested after the silver gel was dried. The preparation process of the sample is shown in Fig. 4.

The experimental equipment was a Concept-80 broadband dielectric spectrum test system, which mainly included control software, an E4991A impedance analyzer and test fixture, as shown in Fig. 5.

2.2. The Propagation equations of Radar Waves in Coal

2.2.1. Dielectric properties of tested coal samples

The relative dielectric constant values of the five tested coals are shown in Fig. 6, and resistivity values are shown in Fig. 7.

It can be seen from Fig. 6 that the relative dielectric constants of five different coals followed similar regularities. They decreased first and then increased with increasing test frequency. The minimum values of dielectric constant appeared around 300 MHz, while the largest one were near 1000 MHz. At the same test frequency, lean

coal had the highest relative dielectric constant, followed by lignite, gas coal, long flame coal, and finally anthracite, as shown in Fig. 6(F). The lignite contained a large number of hydrophilic polar functional groups, which could adsorb a large amount of intrinsic moisture. Polar functional groups and moisture had a high relative dielectric constant. Therefore, the dielectric constant of lignite was higher. The order of relative dielectric constants of the other four ranks of coal should be determined by the carbon content in coal. The relative dielectric constant of lean coal was larger than that of lignite because it was influenced by other factors, including porosity [22]. The relative dielectric constants and frequencies were fitted and were found to satisfy the cubic polynomial relationship. The results are shown in Eq. (1), and correlation coefficients are shown in Table 2.

$$\varepsilon = C_3 f^3 + C_2 f^2 + C_1 f + C_0, \left(f \in \left[100MHz, 1000MHz \right] \right) \quad (1)$$

Where ε is the relative dielectric permittivity, f is frequency, and C_i is a coefficient (i=0, 1, 2, 3).

It could be seen from Fig. 7 that the resistivity of coal with a different degree of metamorphism had an overall decreasing trend with an increase in test frequency. There was a convex transformation point near 500 MHz, which could divide the trend of resistivity with frequency into two stages. It was found, through fitting, that part Ⅰ conformed to a cubic polynomial and part Ⅱ conformed to a quadratic polynomial. The results were shown in Eq. (2) and Table 3. After 700 MHz, the resistivity of tested coals tended to be flat and the differences between them are small. As the test frequency increased, electrons a in bundled state of the coal structure group might be

converted into freely excited electrons. This caused a significant increase in free radicals (ie, unpaired electrons) of coals, so the resistivity would decrease. Previous studies have shown that anthracite was a resistive absorbing material [39], and the resistivity of mineral impurities in it was higher than that of organic matter [40]. These factors might make the anthracite resistivity relatively large.

$$\rho=\begin{cases} C_{13} f^{3}+C_{12} f^{2}+C_{11} f+C_{10}, & (f \in[100MHz,500MHz]) \\ C_{22} f^{2}+C_{21} f+C_{20}, & (f \in(500MHz,1000MHz]) \end{cases} \tag{2}$$

2.2.2. The equation of radar wave propagation in coal

To simplify the model, it was assumed that the coal body was a homogeneous medium. Since radar waves area type of electromagnetic wave, their propagation velocity in coal could also be expressed by Eq. (3) [41]. From Eq. (3) and Fig. 8, the propagation velocity of radar waves in different coals could be obtained, as shown in Table 4.

$$v=\left[\frac{\varepsilon}{2}\left(\sqrt{1+(1/2\pi f \varepsilon \rho)^{2}}+1\right)\right]^{-1/2} \tag{3}$$

Where v is the propagation speed of radar waves in coal, in m/s. ε is the specific relative dielectric permittivity of coal.

According to the literature [42], the attenuation coefficient of electromagnetic wave propagation in coal could be characterized by Eq. (4). Combined with the dielectric constant and electrical conductivity of coal, the variation of attenuation coefficient with frequency could be obtained, as shown in Fig. 8

$$a=2\pi f\left[\frac{\varepsilon\mu}{2}\left(\sqrt{1+(1/2\pi f \rho\varepsilon)^{2}}-1\right)\right]^{1/2} \tag{4}$$

where α is the attenuation coefficient of radar wave in coal, f is the frequency, ε is the relative dielectric permittivity of coal, and ρ is the specific resistivity of coal.

It could be seen from Fig. 8 that the propagation attenuation coefficient of electromagnetic waves in five types of coal followed a similar rule in relation to frequency. It could be divided into two parts, according to the boundary line near 500 MHz. In part I, the attenuation coefficient was a cubic function of frequency. It increased first and then decreased with increasing frequency. At the same frequency, the attenuation coefficients of coals, from large to small, were lean coal, gas coal, long flame coal, anthracite, and lignite. In part II, the attenuation coefficient was a quadratic function of frequency. It increases with increasing frequency. In ①of part Ⅱ, at the same frequency, the attenuation coefficient from large to small was anthracite, long flame coal (gas coal, lean coal), and brown coal. Among them, the attenuation coefficient of long flame coal, gas coal and lean coal had little difference. In ②of part Ⅱ, at the same frequency, the attenuation coefficient from large to small was anthracite (long flame coal, gas coal), lean coal, and brown coal. Among them, the attenuation coefficient of anthracite, long flame coal, and gas coal were similar.

The attenuation coefficient and frequency were fitted, and results were shown in Eq. (5) and Table 5.

$$a=\begin{cases} C'_{13}f^3+C'_{12}f^2+C'_{11}f+C'_{10}, & (f\in[100MHz,500MHz]) \\ C'_{22}f^2+C'_{21}f+C'_{20}, & (f\in(500MHz,1000MHz]) \end{cases} \tag{5}$$

According to literature [43], reflection and refraction coefficients of radar waves propagating in coal could be characterized by Eqs. (6) and (7).

$$\text{Reflection coefficient: } R_{ij} = \frac{\sqrt{\varepsilon_i} - \sqrt{\varepsilon_j}}{\sqrt{\varepsilon_i} + \sqrt{\varepsilon_j}} \quad (6)$$

$$\text{Refraction coefficient: } T_{ij} = \frac{2\sqrt{\varepsilon_i}}{\sqrt{\varepsilon_i} + \sqrt{\varepsilon_j}} \quad (7)$$

where R_{ij} is the reflection coefficient of radar waves at the interface between medium *i* and medium *j*. T_{ij} is the refraction coefficient of radar waves at the interface between medium *i* and medium *j*. ε_i is the relative dielectric constant of medium *i*. ε_j is the relative dielectric constant of medium *j*.

From eqs. (6) and (7), respectively, the reflection and refraction coefficients on various interfaces (as shown in Fig. 9) during radar wave propagation could be obtained. The reflection and refraction coefficients of the interface were calculated by taking the relative dielectric constant of coal at a frequency of 500 MHz as an example. The results are shown in Table 6. It was not difficult to find that a negative number appears in the reflection coefficient because the radar wave from the side with a small relatively dielectric constant moves to the side with high relatively dielectric constant, indicating that the reflected wave was opposite in phase to the incident wave.

The propagation distance of radar waves in coal was determined by the attenuation coefficient. It could be seen from Eq. (4) that the independent variables determining variable α (attenuation coefficient) are f (frequency), ε (relatively dielectric constant), the ρ (resistivity), i.e., $\alpha = F(f, \varepsilon, \rho)$. As can be seen from equation (1), ε (relatively dielectric constant) is a function of f (frequency), i.e., $f = F^{-1}(\varepsilon)$. From Eq. (5), it is found that f (frequency) is a function of α (attenuation

coefficient), i.e., $\alpha = F^{-1}(f)$. Thus, the propagation attenuation law of radar waves in coal is given by

$$\begin{cases} a = 2\pi f\left[\frac{\varepsilon}{2}\left(\sqrt{1+\left(1/2\pi f\rho\varepsilon\right)^{2}}-1\right)\right]^{1/2} \\ a = \begin{cases} C'_{13}f^{3}+C'_{12}f^{2}+C'_{11}f+C'_{10}, & (f\in[100MHz,500MHz]) \\ C'_{22}f^{2}+C'_{21}f+C'_{20}, & (f\in(500MHz,1.0GHz]) \end{cases} \\ \varepsilon = C_{3}f^{3}+C_{2}f^{2}+C_{1}f+C_{0}, \left(f\in[100MHz,1.0GHz]\right) \\ \rho = \begin{cases} C_{13}f^{3}+C_{12}f^{2}+C_{11}f+C_{10}, & (f\in[100MHz,500MHz]) \\ C_{22}f^{2}+C_{21}f+C_{20}, & \left(f\in(500MHz,1.0GHz]\right) \end{cases} \end{cases} \tag{8}$$

where α is the attenuation coefficient of radar wave propagation in coal. f is the frequency. ε is a relative dielectric constant. ρ is the resistivity. C'_{ij}, C_i, $C_{ij}, \left(i, j\in[0,1,2,3]\right)$ are the coefficients of the corresponding function, respectively.

It was necessary to analyze the characteristics of waves to determine whether there was anyone behind the coal when using radar for rescue. The characteristics of waves were determined by laws of refraction and reflection, that is, Eq. (9), consisting of Eqs. (6) and (7).

$$\begin{cases} R_{ij} = \frac{\sqrt{\varepsilon_i}-\sqrt{\varepsilon_j}}{\sqrt{\varepsilon_i}+\sqrt{\varepsilon_j}} \\ T_{ij} = \frac{2\sqrt{\varepsilon_i}}{\sqrt{\varepsilon_i}+\sqrt{\varepsilon_j}} \end{cases} \tag{9}$$

where ε_i and ε_j are the relative dielectric constants of substance i and j, respectively. R_{ij} and T_{ij} are the reflection and refraction coefficients, respectively.

According to the relationship between distance, speed and time, the distance between the radar and person in distress can be calculated according to the following formula.

$$\begin{cases} L = v \cdot t \\ v = \left[\frac{\varepsilon}{2} \left(\sqrt{1 + (1/2\pi f \varepsilon \rho)^2} + 1 \right) \right]^{-1/2} \end{cases} \tag{10}$$

where v is the propagation speed of radar waves in coal, in m/s.

The equations formed by the combination of Eqs. (8), (9), and (10) were the control equations for the propagation of radar waves in coal.

$$\begin{cases} a = 2\pi f \left[\frac{\varepsilon}{2} \left(\sqrt{1 + (1/2\pi f \rho \varepsilon)^2} - 1 \right) \right]^{1/2} \\ a = \begin{cases} C'_{13} f^3 + C'_{12} f^2 + C'_{11} f + C'_{10}, (f \in [100MHz, 500MHz]) \\ C'_{22} f^2 + C'_{21} f + C'_{20}, (f \in (500MHz, 1.0GHz]) \end{cases} \\ \varepsilon = C_3 f^3 + C_2 f^2 + C_1 f + C_0, (f \in [100MHz, 1.0GHz]) \\ \rho = \begin{cases} C_{13} f^3 + C_{12} f^2 + C_{11} f + C_{10}, (f \in [100MHz, 500MHz]) \\ C_{22} f^2 + C_{21} f + C_{20}, (f \in (500MHz, 1.0GHz]) \end{cases} \\ R_{ij} = \frac{\sqrt{\varepsilon_i} - \sqrt{\varepsilon_j}}{\sqrt{\varepsilon_i} + \sqrt{\varepsilon_j}} \\ T_{ij} = \frac{2\sqrt{\varepsilon_i}}{\sqrt{\varepsilon_i} + \sqrt{\varepsilon_j}} \\ v = \left[\frac{\varepsilon}{2} \left(\sqrt{1 + (1/2\pi f \varepsilon \rho)^2} + 1 \right) \right]^{-1/2} \\ L = v \cdot t \end{cases} \tag{11}$$

3. Results and discussion

The equations (11) could be solved using GprMax code. GprMax is a simulation software based on FDTD theory, which was developed by Dr. Antonis Giannopoulos of the University of Edinburgh [44]. The GprMax has been widely used for simulation and modelling of radar waves propagation in such GPR applications as underground pipeline surveys, roadbed inspections, ancient building surveys, military surveys, quality inspections, and hidden grave surveys [45-49]. The simulation calculation process is shown in Fig. 10. The simulated physical model is shown in Fig. 11(I).

As shown in Fig. 11(II), the thickness of a coal body between a trapped person and radar was L_1, the distance between a person and coal wall was L_2, the width of the

model was L_3, the length of model was L_4, and the size of a human body is 1.6 m ×0.4 m. The specific settings of different models are shown in Table 7. Then, the simulated data was post-processed using Matlab, and the result is shown in Fig. 11(Ⅲ).

It was assumed that coal was a semi-infinite continuous space with homogeneous isotropy. The dielectric constant of a human was 50 [50]. The dielectric constant of air was 1, the antenna stepping distance was 0.06 m, and the transmitting and receiving antenna spacing was 0.065 m. The number of measuring lines was 115, the spatial grid step was 0.005 m×0.005 m, the permeability of all media in the model was 1.0, the boundary condition was a perfect matching layer (PML), the excitation function of the radar was Ricker, and the center frequency was 500 MHz. The partial electrical parameters of the model were set as shown in Table 8. Due to the large number of graphs of simulation results, only lignite was taken as an example for analysis here. The results are shown in Fig. 12.

From Fig. 12, we found that the amplitudes of return wave from the interface between the coal pillar and roadway in seven models were 12.51, 5.48, 2.56, 1.23, 0.63, 0.30, and 0.15 mV/m, and the return amplitude of the human body is -20.63. -10.24, -5.30, -2.72, -1.37, -0.69, and -0.35 mV/m. It was not difficult to find that the intensity of amplitude gradually decreased with the increase of L_1 (thickness of the coal pillar). The larger the L_1, the longer the electromagnetic wave travels in coal, and the loss of electromagnetic wave energy also increased, so the intensity of the target return wave amplitude was gradually smaller. By fitting, it was found that the amplitude of the human body wave decreases with the increase of L_1 as an

exponential function (as shown in Fig. 13). The effective propagation distance of radar waves in coal was between 6 and 8 m for transducer with central frequency 500 MHz.

4. Conclusions

Dielectric properties of five different coals in from Chinese deposits were studied using the alternating current impedance method and the Concept-80 broadband dielectric Spectrum test system for electromagnetic waves with frequencies ranging from 100 MHz to 1000 MHz, for the first time, to the best of our knowledge. The propagation process of radar waves in coal was analyzed.

The experimental results showed that the dielectric constants of five different coals followed similar rules. The values of relative dielectric constant of coals decreased first and then increased with frequency. At the same frequency, the order of values of the relative permittivity of coal from high to low was lean coal, lignite, gas coal, long flame coal, and anthracite. The values of specific resistivity of coal decreased considerably with increasing frequency, but there was observed an upward bump around frequency 500 MHz.

The key parameters of radar waves propagating in coal were analyzed. The propagation velocity values of radar waves in different rank coals and the reflection and refraction coefficients values at different types of interfaces were calculated. The attenuation coefficient of radar waves in five kinds of coals followed a similar rule.

With 500 MHz as the dividing point, the relation between attenuation coefficient of radar waves and frequency was divided into two parts. In part Ⅰ, the frequency was

a cubic function of the attenuation coefficient. The attenuation coefficient initially increases to a certain point and then decreases with increasing of frequency values. In part Ⅱ, the frequency was a quadratic function of the attenuation coefficient, and the values of attenuation coefficient increased with frequency increase. A set of equations for the propagation of radar waves in coal were established, based on the above researches. By analyzing the governing equations, it was found that the effective penetration distance of radar wave with dominant frequency 500MHz in coal ranges is between 6 and 8 m.

Acknowledgements

Funding: The project was supported by the National Natural Science Foundation of China (grant numbers 51904234 and 51974240) and the Key R & D Program of Shaanxi Provincial (grant number 2017ZDCXL-GY-01-02-03).

References

1. Guo L, Zhai M, Wang Z, Zhang Y, Dong P. Comprehensive coal quality index for evaluation of coal agglomeration characteristics, Fuel 2018; 231(12): 379-386.

2. Song H, Liu J, Xue F, Cheng F. The application of ultra-fine fly ash in the seal coating for the wall of underground coal mine, Advanced Powder Technology 2016; 27: 1645-1650.

3. Diniz DA, Prasad P, Pagan RJ. Modern environmental improvement pathways for the coal power generation industry in Australia. Process Safety and Environmental Protection 2004; 82: 191-199.

4. Warmuzinski K. Harnessing methane emissions from coal mining. Process Safety and Environmental Protection 2008; 86: 315-320.

5. British Petroleum (BP), Statistical Review of World Energy, 2015. http://www.bp. ucom/statistical review.

6. Wang D. Thermodynamic disaster in coal mine and its characteristics. Journal of China Coal Society 2018; 43(1): 137-142.

7. Wang K, Jiang S, Ma X, Wu Z, Shao H, Zhang W, et al. Information fusion of plume control and personnel escape during the emergency rescue of external-caused fire in a coal mine. Process Safety and Environmental Protection 2016; 103: 46-59.

8. Ken B. Buried alive, but carbide drills at core of Chile miners' rescue. Metal Powder Report 2010; 65(7): 7-10.

9. Yang T, Du B. Construction Technology of Large Diameter rescue borehole in Pingyi gypsum mine disaster of Shandong, Exploration Engineering (Rock & Soil Drilling and Tunneling) 2017, 44(5): 19-23. (In Chinese)

10. Wen H, Zhang D, Zheng X-Z. Research Progress and Trend of Life Detection Technology of Drilling and Rescue in Mine. Safety in Coal Mines 2017, 48(9): 85-88. (In Chinese)

11. Li Z, Li W, Lv H, Zhang Y, Jing X, Wang J. A novel method for respiration-like clutter cancellation in life detection by dual-frequency IR-UWB radar, IEEE Transactions on Microwave Theory and Techniques 2013; 61 (5): 2086-2092.

12. Hu W, Zhao Z, Wang Y, Zhang H, Lin F. Noncontact accurate measurement of cardiopulmonary activity using a compact quadrature Doppler radar sensor, I IEEE Transactions on Biomedical Engineering 2014; 61 (3): 725-735.

13. Chen K. Huang Y, Zhang J, Norman A. Microwave life-detection systems for searching human subjects under earthquake rubble or behind barrier, IEEE Transactions on Biomedical Engineering 2000; 47 (1): 105-114.

14. JalaliBidgoli F, Moghadami S, Ardalan S. A compact portable microwave lifedetection device for finding survivors. IEEE Embedded Systems Letters 2016; 8(1): 10-13.

15. Liang X, Zhang H, Fang G, Ye S, Gulliver T. An improved algorithm for throughwall target detection using ultra-wideband impulse radar. IEEE Access 2017; (99): 22101-22118.

16. Guan S, Rice JA, Li C, Gu C. Automated dc offset calibration strategy for structural health monitoring based on portable cw radar sensor. IEEE Transactions on Instrumentation and Measurement 2014; 63(12): 3111-3118.

17. Wang F, Horng TS, Peng K, Jau J, Li J, Chen C. Single-antenna Doppler radars using self and mutual injection locking for vital sign detection with random body movement cancellation. IEEE Transactinos on Microwave Theory and Techniques 2011; 59 (12): 3577-3587.

18. Liang X, Zhang H, Lyu T, Xu L, Cao C, Gulliver TA. Ultra-wide band impulse radar for life

detection using wavelet packet decomposition. Physical Communication 2018; 29:31-47.

19. Fan W, Jia C-Y, Hu W, Yang C, Liu L, Zhang X, et al. Dielectric properties of coals in the low-terahertz frequency region. Fuel 2015; 162: 294-304.

20. Fan W, Chang T, Liu L, Cui H. Dielectric properties of coal in the terahertz frequency region of 100–500 GHz. Fuel 2017; 188: 246-253.

21. Qin F, Wei G. Measurement with Sweeping Frequency for Complex Permittivity Based on VNA. Journal of Astronautic Metrology and Measurement 2011; 31(3): 27-30.

22. Brach I, Giuntini JC, Zanchetta JC. Real part of the permittivity of coals and their rank. Fuel 1994; 73(5): 738-741.

23. Marland S, Merchant A., Rowson N. Dielectric properties of coal. Fuel 2001; 80: 1839-1849.

24. Valentina VZ. Influence of polyethylene terephthalate on the carbonisation of bituminous coals and on the modification of their electric and dielectric properties. Fuel; 2006, 85:1652-1665.

25. Wang Q, Zhang X, Gu F. Investigation on interior moisture distribution inducing dielectric anisotropy of coals. Fuel processing technology 2005; 89:633-641.

26. Peng Z, Lin X, Li Z, Hwang J, Kim B, Zhang Y, et al. Dielectric characterization of Indonesian low-rank coal for microwave processing. Fuel processing technology 2017; 156: 171-177.

27. Tao X, Tang L, Xie M, He H, Xu N, Feng L, et al. Dielectric properties analysis of sulfur-containing models in coal and energy evaluation of their sulfur-containing bond dissociation in microwave field. Fuel 2016; 181: 1027-1033.

28. Xu L, Liu H, Jin Y, Fan B, Qiao X, Jing B. Structural order and dielectric properties of coal chars. Fuel 2014; 137: 164-171.

29. Liu H, Xu L, Jin Y, Fan B, Qiao X, Yang Y. Effect of coal rank on structure and dielectric

properties of chars. Fuel 2015; 153: 249-256.

30. Emslie AG, Lagace RL. Propagation of low and medium frequency radio waves in a coal seam. Radio Science 1976; (11): 253-261.

31. Hill DA. Radio propagation in a coal seam and the inverse problem. Journal of Research of the National Bureau of Standards 1984; (89): 385-394.

32. Hill DA. Electromagnetic detection of long conductors in tunnels. IEEE Trans 1985; 33(4).

33. Greenfield RJ,Wu ST. Electromagnetic wave propagation in disrupted coal seams. Geophysics 1991; 56(10): 1571-1577.

34. Luo Y, Stolarczyk LG, Peng SD. Demonstration of the advanced radio imaging method (rim): ground truth results for three-dimensional imaging of coal seams. Sme Meeting 2004.

35. Stolarczyk LG, Fry RC. Radio imaging method (RIM) or diagnostic imaging of anomalous geologic structures in coal seam waveguides. Transactions of Society for Mining, Metallurgy, and Exploration, Inc. 1990; (288): 1806-1814.

36. Stolarczyk LG. Electromagnetic seam wave mapping of roof rock conditions across a longwall panel. 18th International Conference on Ground Control in Mining, Aug 3-5.1999.

37. Stolarczyk LG, Peng SS, Luo Y. Imaging ahead of Mining with Radio Imaging Method (RIM-IV) Instrumentation and Three-dimensional Tomography. Software Proceedings of 22nd International Conference on Ground Control in Mining 2003, Morgantown, WV, 136-143.

38. Zhu H, Xin M, Chang M, Liu D. Research progress on measuring technology of coal dielectric constant. Coal Science Technology 2016; 44(9): 6-12.

39. Singclair DC, West AR. Impedance and modulus spectroscopy of semiconducting $BaTiO_3$

showing positive temperature coefficient of resistance. Journal of Applied. Physics 1989; 66(8): 3850-3856.

40. Feng X, Chen J, Li N, Cui H, Liu J. Study on electromagnetic properties of anthracite and soft coal in microwave field. Study on electromagnetic properties of anthracite 2007; 38(5): 405-411. (In Chinses)

41. Zhou, B. and Fullagar, P.K., 2001, Delineation of sulphide ore-zones by borehole radar tomography at Hellyer Mine, Australia: Journal of Applied Geophysics, 47, No 3-4, pp.261-269.

42. Qi C. Ground penetrating radar signal processing in advanced detection in coal seam, Xi'an university of science and technology 2014. (In Chinese)

43. Liu X. Research the propagation rules of middle-high frequency electromagnetic wave in coal medium. China University of Mining and Technology 2012.

44. Giannopoulos A. Modelling ground penetrating radar by GprMax. Construction and Building Materials 2005; 19: 755-762.

45. Núñez-Nieto X, Solla M, Novob A, Lorenzo H. Three-dimensional ground-penetrating radar methodologies for thecharacterization and volumetric reconstruction of underground tunneling. Construction and Building Materials 2014; 71: 551-560.

46. Adam DB, Jamie K, Pringle. Semblance analysis to assess GPR data from a five-year forensic study of simulated clandestine graves. Journal of Applied Geophysics 2016; 125: 17-44.

47. Lopera O, Milisavljevic N. Prediction of the effects of soil and target properties on the antipersonnel landmine detection performance of ground- penetrating radar: A Colombian case study. Journal of Applied Geophysics 2007; 63: 13-23.

48. Shangguan P, Imad L. AQ, Lahouar S. Pattern recognition algorithms for density estimation of asphalt pavement during compaction: a simulation study. Journal of Applied Geophysics 2014; 107: 8-15.

49. Jaw SW, Hashim M. Locational accuracy of underground utility mapping using ground penetrating radar. Tunnelling and Underground Space Technology Incorporating Trenchless Technology Research 2013; 35: 20-29.

50. Dogaru T, Nguyen L, Le C. Computer models of the human body signature for sensing through the wall radar applications. DTIC Document Adelphi MD 2007.

51. Lee JH, Wang W. Characterization of snow cover using ground penetrating radar for vehicle trafficability–Experiments and modeling. Journal of Terramechanics 2009; 46: 189-202.

Table captions

Table 1 Proximate and elemental analysis of coal.

Table 2 Coefficients of fitting equation.

Table 3 Coefficients of fitting equation.

Table 4 Propagation speed of radar waves in coal (m/s).

Table 5 Coefficients of fitting equation.

Table 6 Reflection and refraction coefficients.

Table 7 Size of model (m).

Table 8 Dielectric characterization of Coal.

Table 1 Proximate and elemental analysis of coal.

Coal	Proximate analysis (%)				Elemental analysis (%)					Porosity
	M_{ad}	A_{ad}	V_{ad}	FC_{ad}	C	H	O	N	S	(%)
1#	8.80	36.62	24.68	29.90	38.94	4.01	22.00	1.15	0.27	10.11
2#	5.77	11.93	32.32	49.98	64.86	5.63	19.20	1.73	0.58	13.34
3#	2.33	15.92	35.45	46.30	63.92	4.85	17.40	2.37	0.51	6.16
4#	0.53	24.49	12.95	62.03	62.51	3.76	17.00	1.61	3.41	9.19
5#	1.20	8.84	7.85	82.11	81.82	3.79	4.90	0.85	1.49	16.20

Table 2 Coefficients of fitting equation.

Coals	C_3	C_2	C_1	C_0	R^2（%）
1#	-9.24×10^{-10}	1.84×10^{-6}	-9.14×10^{-4}	3.62	98.98
2#	-5.82×10^{-10}	1.17×10^{-6}	-5.43×10^{-4}	3.07	98.77
3#	-6.34×10^{-10}	1.28×10^{-6}	-5.96×10^{-4}	3.35	99.21
4#	-7.90×10^{-10}	1.52×10^{-6}	-6.58×10^{-4}	3.78	99.40
5#	-6.42×10^{-10}	1.34×10^{-6}	-6.86×10^{-4}	3.05	98.49

Table 3 Coefficients of fitting equation.

Partion	Coefficient	1#	2#	3#	4#	5#
I	C_{13}	-8.00×10^{-5}	-5.45×10^{-5}	-4.96×10^{-5}	-4.08×10^{-5}	-7.48×10^{-5}
	C_{12}	0.10	0.07	0.06	0.05	0.10
	C_{11}	-43.06	-30.19	-27.52	-23.03	-40.16
	C_{10}	6.13×10^{3}	4.45×10^{3}	4.06×10^{3}	3.43×10^{3}	5.78×10^{3}
	R^2	98.40 %	99.25 %	99.25 %	99.24 %	98.95 %
II	C_{22}	1.28×10^{-3}	9.50×10^{-4}	8.74×10^{-4}	7.99×10^{-4}	1.12×10^{-3}
	C_{21}	-2.36	-1.81	-1.66	-1.48	-2.22
	C_{20}	1.21×10^{3}	9.60×10^{3}	8.80×10^{2}	7.86×10^{2}	1.10×10^{3}
	R^2	97.94 %	99.11 %	98.84 %	98.58 %	98.91 %

Table 4 Propagation speed of radar waves in coal (m/s).

Frequency (MHz)	1#	2#	3#	4#	5#
100	15.70×10^{7}	17.23×10^{7}	16.14×10^{7}	15.53×10^{7}	17.32×10^{7}
200	16.01×10^{7}	17.29×10^{7}	16.64×10^{7}	15.57×10^{7}	17.43×10^{7}
300	16.05×10^{7}	17.32×10^{7}	16.90×10^{7}	15.59×10^{7}	17.46×10^{7}
400	16.03×10^{7}	17.29×10^{7}	16.64×10^{7}	15.55×10^{7}	17.46×10^{7}
500	16.01×10^{7}	17.23×10^{7}	16.14×10^{7}	15.53×10^{7}	17.39×10^{7}
600	15.94×10^{7}	17.20×10^{7}	16.64×10^{7}	15.45×10^{7}	17.37×10^{7}
700	15.87×10^{7}	17.12×10^{7}	16.15×10^{7}	15.38×10^{7}	17.29×10^{7}
800	15.81×10^{7}	17.66×10^{7}	16.42×10^{7}	15.32×10^{7}	17.26×10^{7}
900	15.76×10^{7}	17.14×10^{7}	16.29×10^{7}	15.30×10^{7}	17.17×10^{7}
1000	15.74×10^{7}	16.84×10^{7}	16.26×10^{7}	15.26×10^{7}	17.14×10^{7}

Table 5 Coefficients of fitting equation.

Part	Coefficient	1#	2#	3#	4#	5#
I	C'_{13}	-8.31×10^{-14}	-6.70×10^{-14}	-7.01×10^{-14}	-7.58×10^{-14}	-6.91×10^{-14}
	C'_{12}	6.36×10^{-11}	4.95×10^{-11}	5.17×10^{-11}	5.55×10^{-11}	5.21×10^{-11}
	C'_{11}	-1.03×10^{-8}	-6.20×10^{-9}	-6.46×10^{-9}	-6.90×10^{-9}	-7.25×10^{-9}
	C'_{10}	$4.71\times10^{--7}$	1.97×10^{-7}	2.05×10^{-7}	2.25×10^{-7}	2.52×10^{-7}
	R^2	99.36%	99.72%	99.72%	99.71%	99.69%
II	C'_{22}	-3.31×10^{-12}	-1.31×10^{-12}	-2.22×10^{-12}	-4.63×10^{-12}	-6.46×10^{-11}
	C'_{21}	8.44×10^{-9}	6.78×10^{-9}	7.90×10^{-9}	1.04×10^{-8}	6.12×10^{-9}
	C'_{20}	-2.61×10^{-6}	2.04×10^{-6}	-2.34×10^{-6}	-2.94×10^{-6}	-1.97×10^{-6}
	R^2	98.88%	99.52%	99.37%	98.66%	99.58%

Table 6 Reflection and refraction coefficients.

Coal	Type	(1) air→coal	(2) coal→air	(3) air→person	(4) person→air	(5) coal→person	(6) person→coal
1#	R_{ij}	-0.31	0.31	-0.75	0.75	-0.58	0.58
	T_{ij}	0.698	1.31	0.25	1.75	0.42	1.58
2#	R_{ij}	-0.27	0.27	-0.75	0.75	-0.60	0.60
	T_{ij}	0.28	1.27	0.25	1.75	0.40	1.60
3#	R_{ij}	-0.29	0.29	-0.75	0.75	-0.59	0.59
	T_{ij}	0.71	1.29	0.25	1.75	0.41	1.59
4#	R_{ij}	-0.32	0.32	-0.75	0.75	-0.57	0.57
	T_{ij}	0.68	1.32	0.25	1.75	0.43	1.57
5#	R_{ij}	-0.27	0.27	-0.75	0.75	-0.61	0.67
	T_{ij}	0.737	1.27	0.25	1.75	0.39	1.67

Table 7 Size of model (m).

Model	M1	M2	M3	M4	M5	M6	M7
L_1	2	3	4	5	6	7	8
L_2	1.3	1.3	1.3	1.3	1.3	1.3	1.3
L_3	3.3	4.3	5.3	6.3	7.3	8.3	9.3
L_4	10	10	10	10	10	10	10

Table 8 Dielectric characterization of Coal.

Coal	1#	2#	3#	4#	5#
ε	3.51	3.03	3.30	3.73	2.99
ρ (Ω·m)	328.95	290.07	267.78	242.41	318.53

Figure captions

Fig. 1. Schematic concept of and application of borehole GPR method for location of miner trapped in underground tunnel in coal mine.

Fig. 2. Flow chart of processing test of coal samples.

Fig. 3. Concept-80 Broadband Dielectric Spectrum Test System.

Fig. 4. Changes of the relative dielectric permittivity with frequency for various coals.

Fig. 5. Variations of various coals resistivity with frequency.

Fig. 6. Changes of attenuation coefficient with frequency.

Fig. 7. Schematic diagram of types of reflection and refraction.

Fig. 8. Simulation calculation flow.

Fig. 9. Geometrical model of rock strata applied in numerical simulation.

Fig. 10. A plot of signal amplitudes versus travel time.

Fig. 11. Relationship (or a plot) between amplitude of a radar signal reflected from a person and a coal seam thickness L_1.

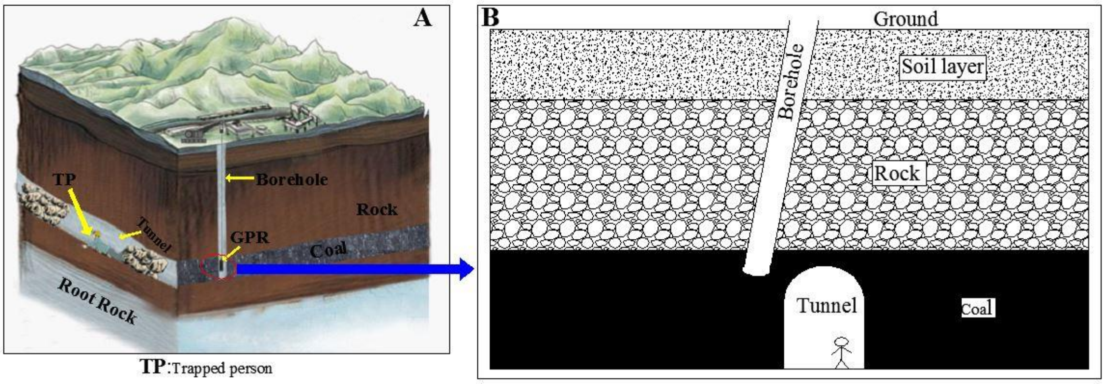

Fig. 1. Schematic concept of and application of borehole GPR method for location of miner trapped in underground tunnel in coal mine.

A is a three-dimensional diagram of rescue drilling during the tunnel collapse； B is a schematic diagram of the drilling offset.

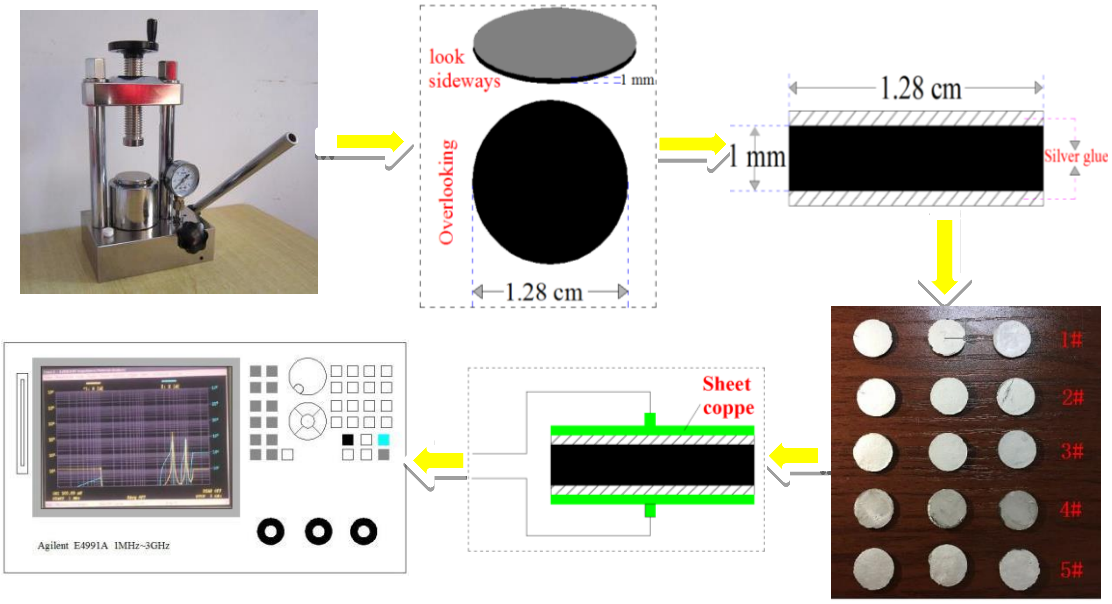

Fig. 2. Flow chart of processing test of coal samples.

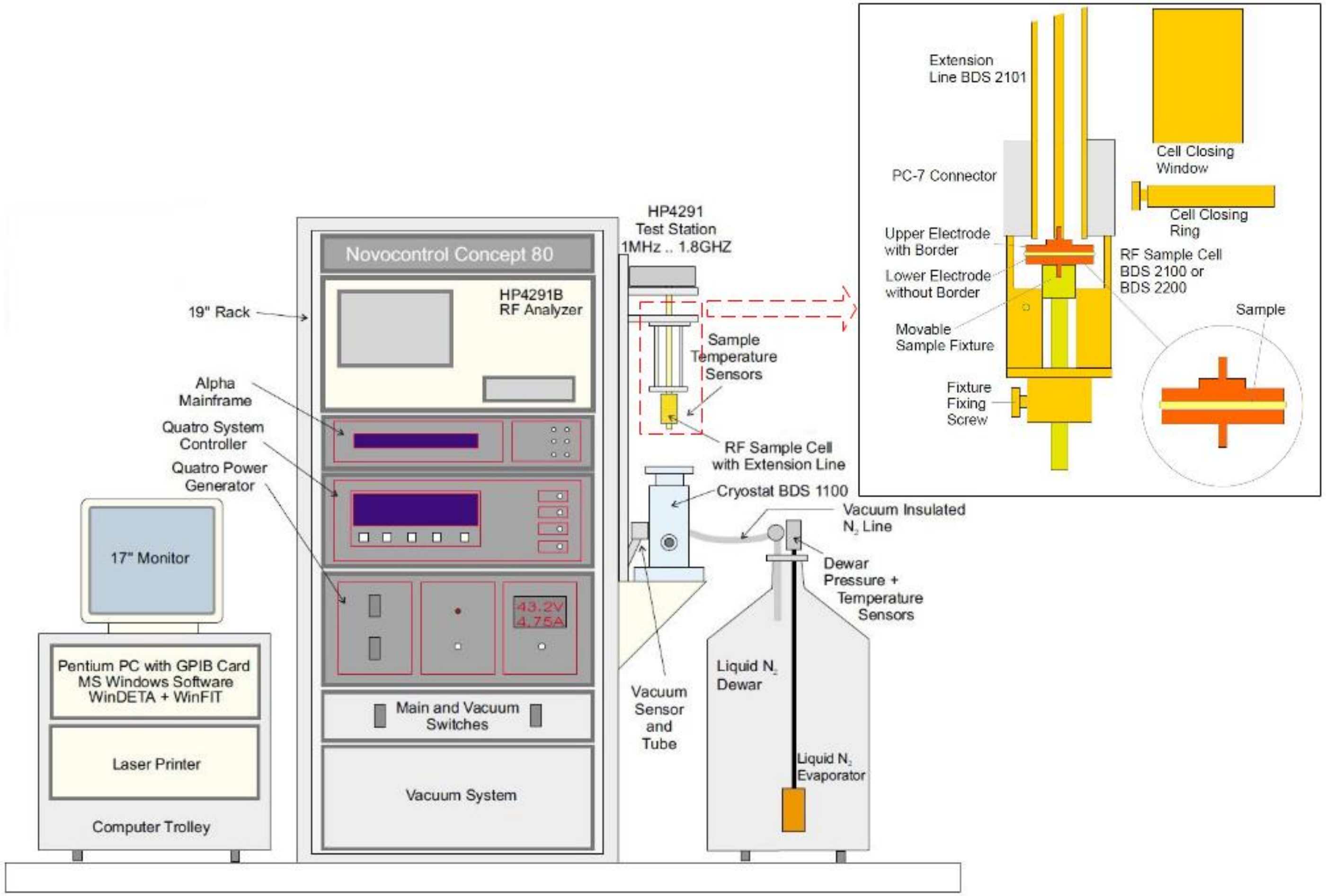

Fig. 3. Concept-80 Broadband Dielectric Spectrum Test System.

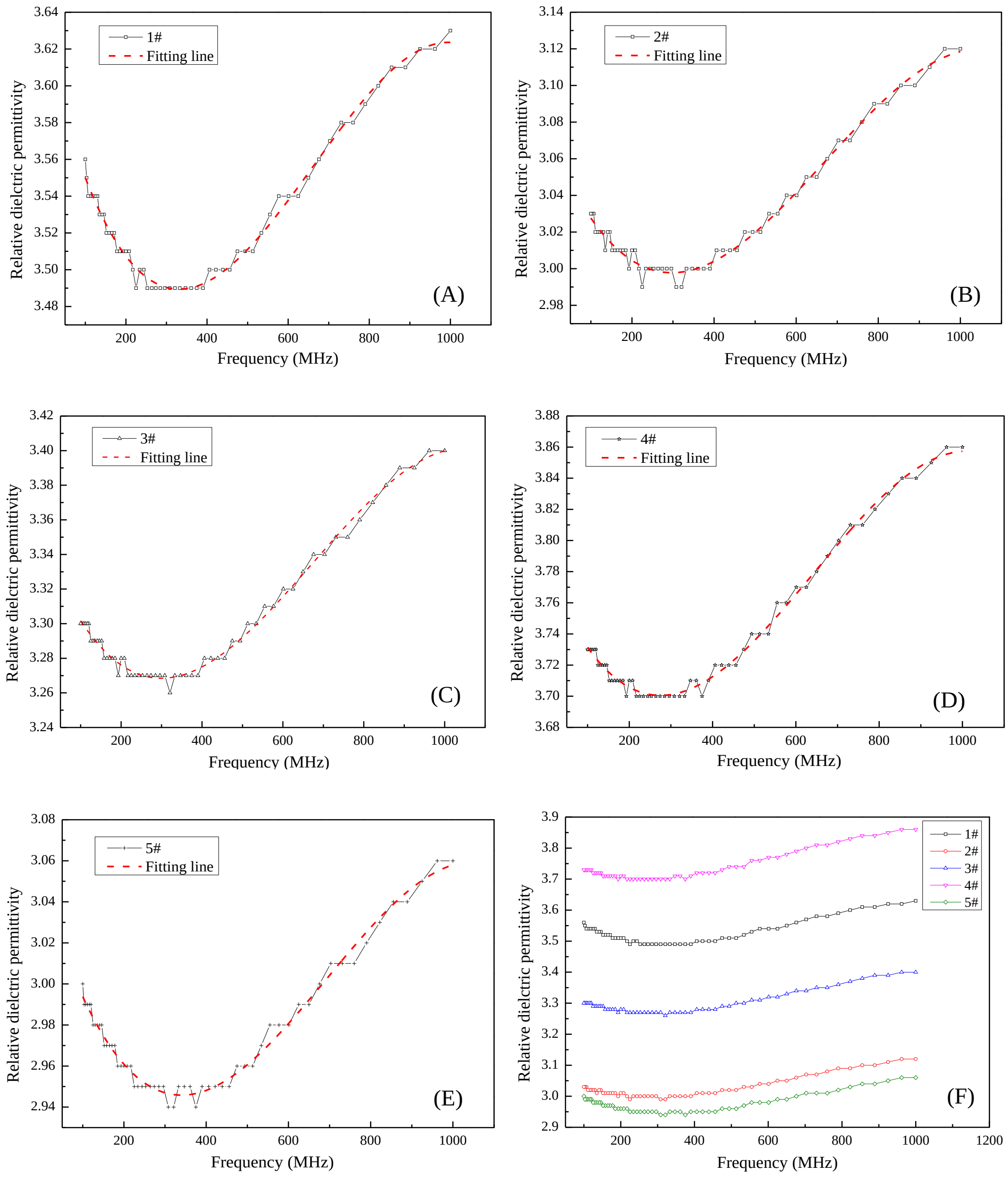

Fig. 4. Changes of the relative dielectric permittivity with frequency for various coals

A: lignite; B: long flame coal; C: gas coal; D: lean coal; E: anthracite;

F: various coals.

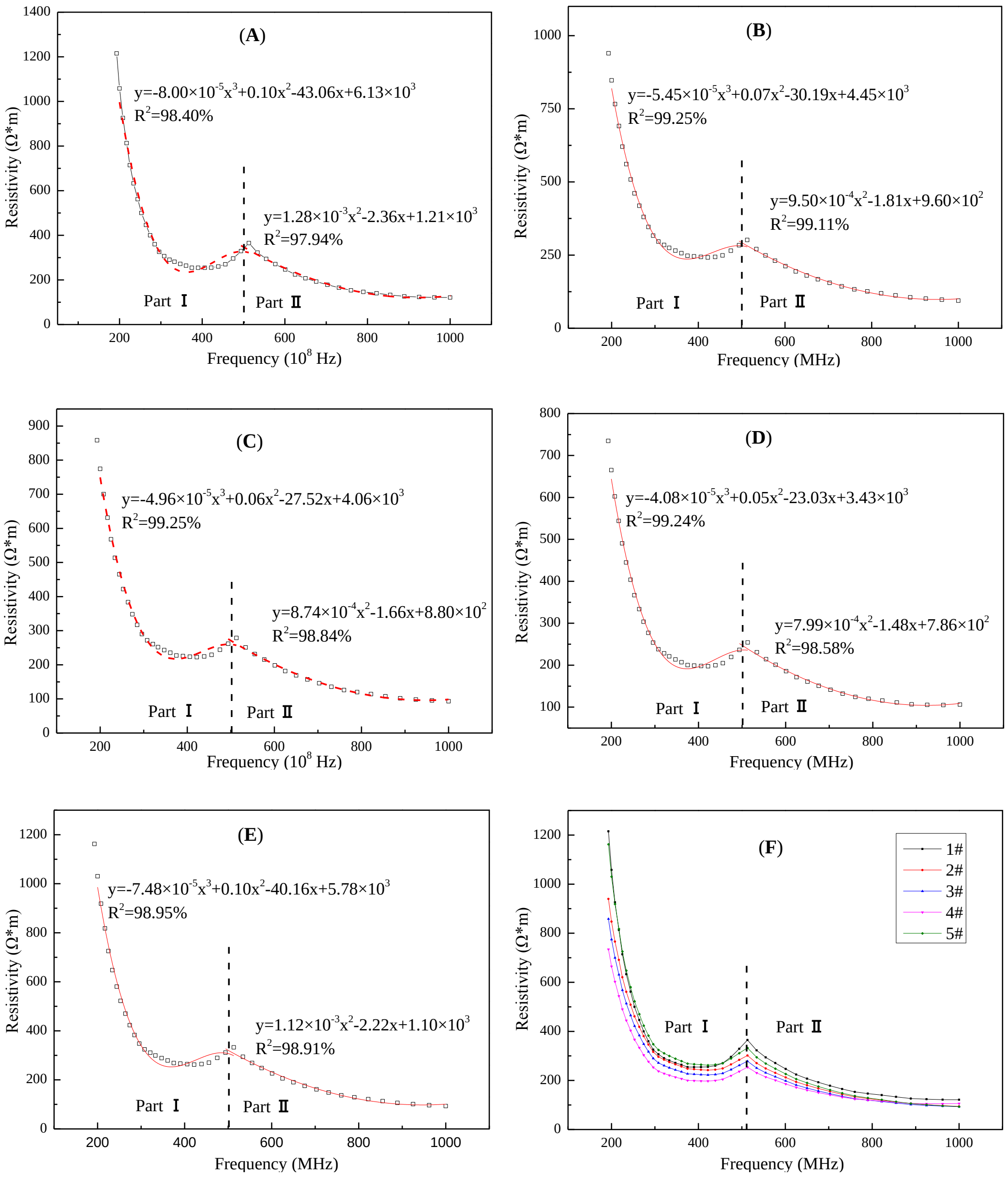

Fig. 5. Variations of various coals resistivity with frequency.

A: lignite; B: long flame coal; C: gas coal; D: lean coal; E: anthracite; F: various coals.

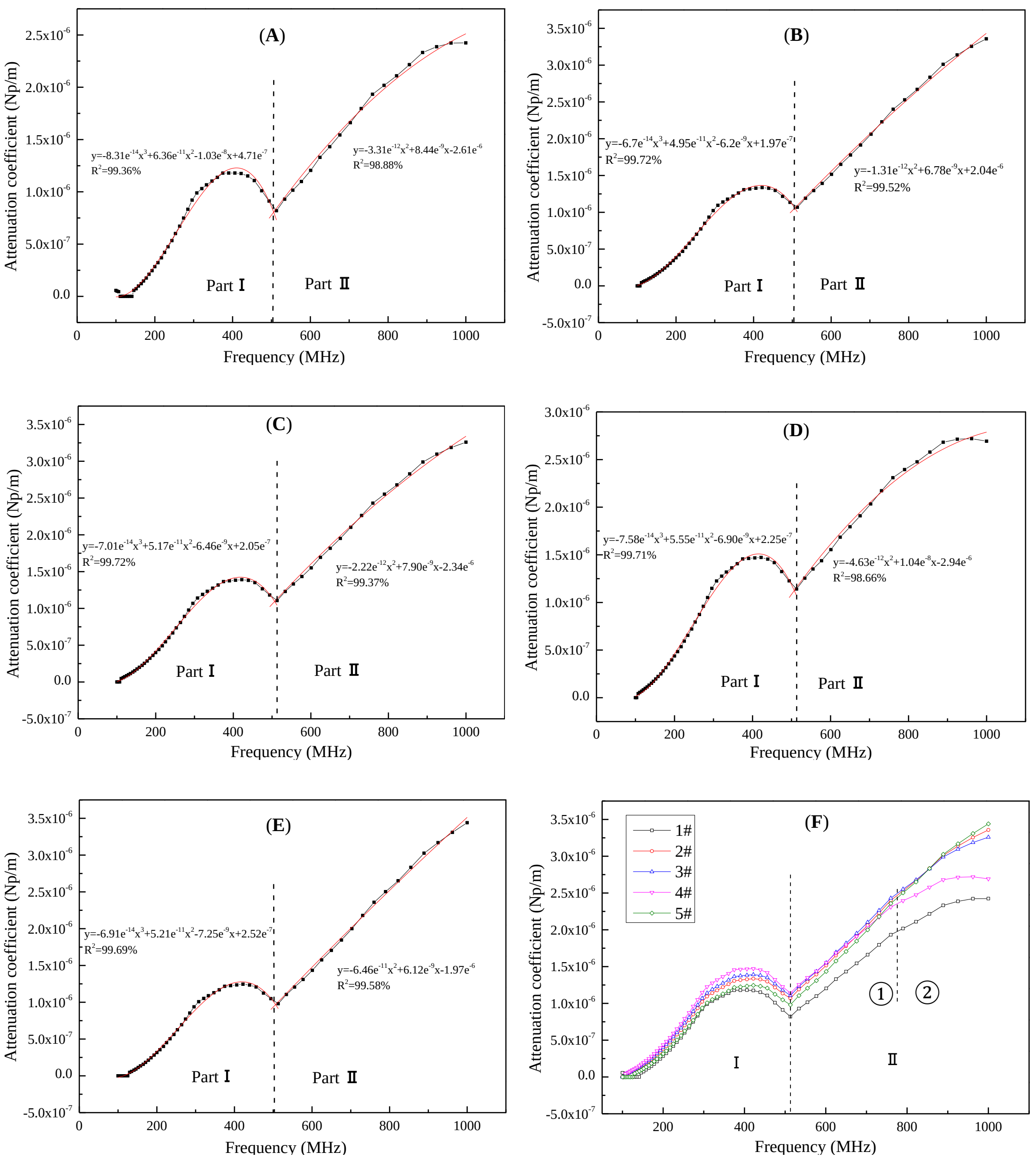

Fig. 6. Changes of attenuation coefficient with frequency.

A: lignite; B: long flame coal; C: gas coal; D: lean coal; E: anthracite; F: various coals.

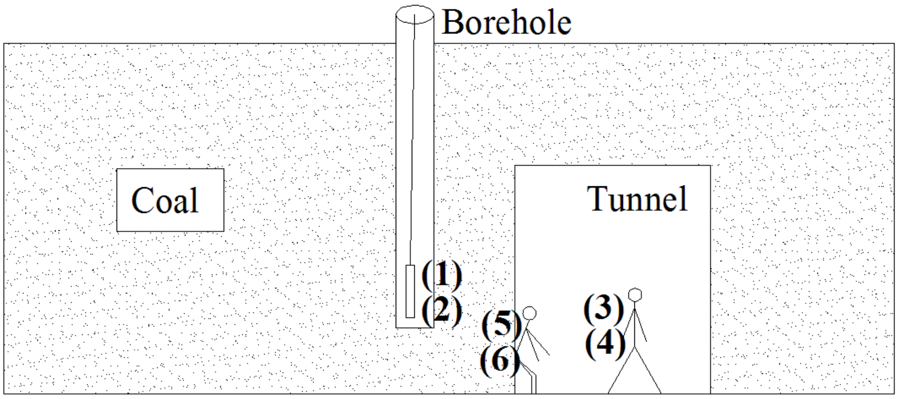

Fig. 7. Schematic diagram of types of reflection and refraction.

Radar waves travel from one object to another. (1): air→coal; (2): coal→air; (3): air→person; (4): person→air; (5): coal→person; (6): person→coal.

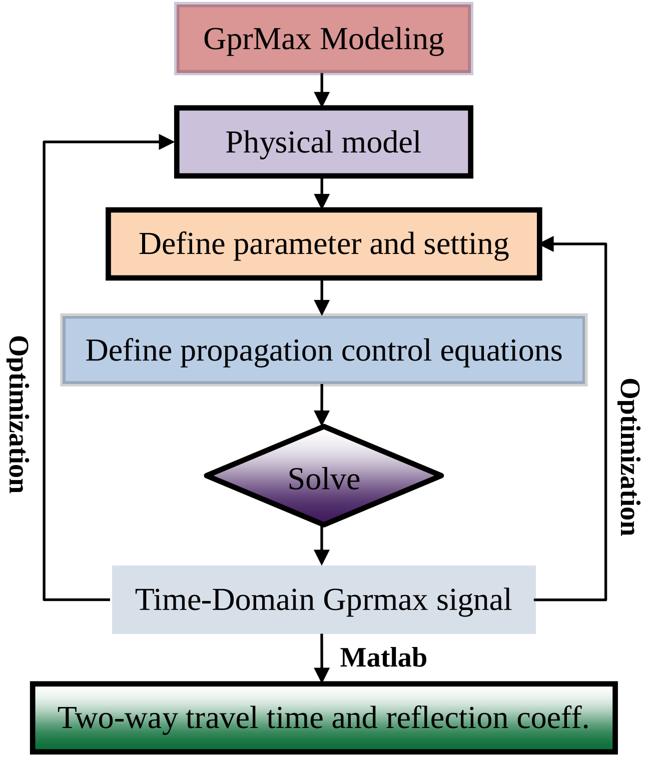

Fig. 8. Simulation calculation flow.

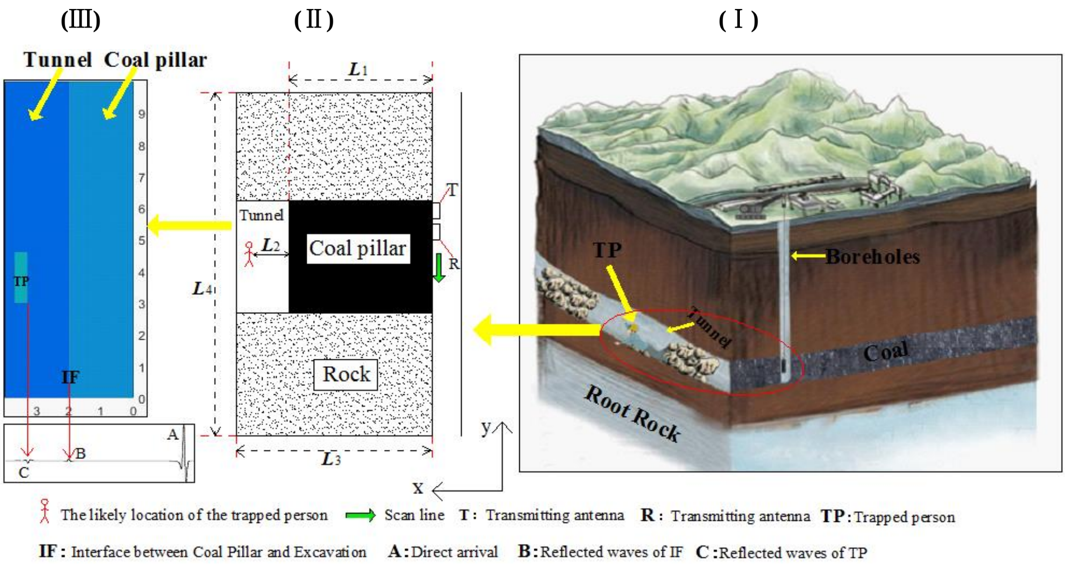

Fig. 9. Geometrical model of rock strata applied in numerical simulation.

Ⅰ: Schematic of illustration of a disaster; Ⅱ: Schematic of simplified of physical model; Ⅲ: Display of physical model in GPR software.

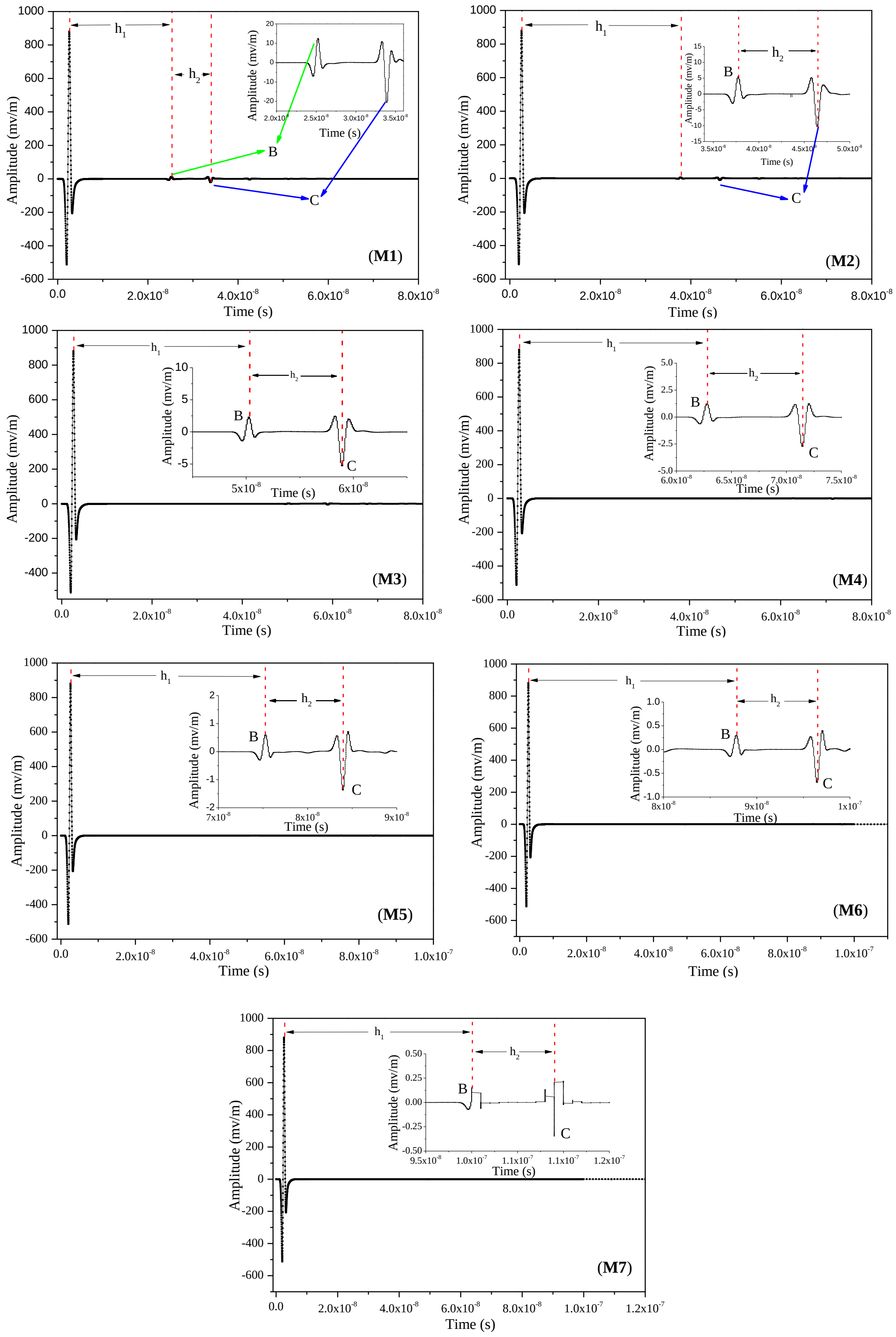

Fig. 10. A plot of signal amplitudes versus travel time.

Mx m are the thickness of the coal body between the radar and the trapped person.

M1: 2 m; M2: 3 m; M3: 4 m; M4: 5 m; M5: 6 m; M6: 7 m; M7: 8 m.

h_i is two-way travel time of radar wave between two objects [52]. The meaning of B and C is given in Fig. 9

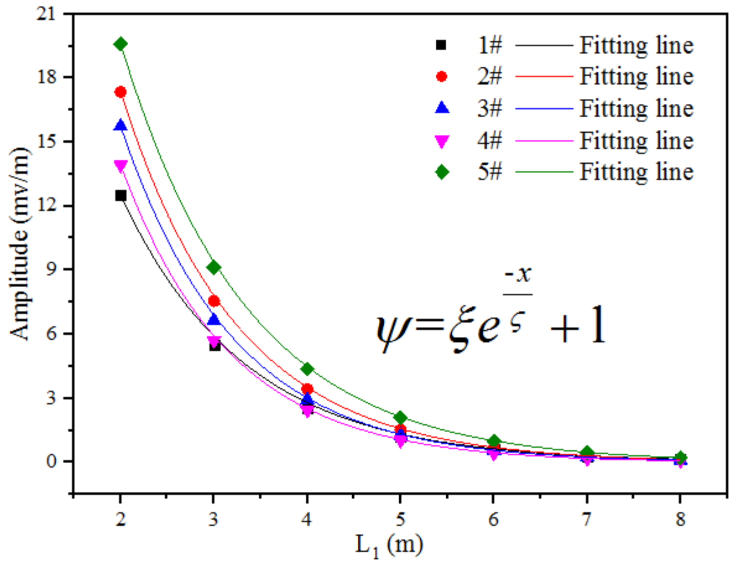

Fig. 11. Relationship (or a plot) between amplitude of a radar signal reflected from a person and a coal seam thickness L1.

Reply to Reviewers' Comments

Journal: PLOS ONE

Manuscript ID: PONE-D-19-29325

Title: "Propagation of Ground Penetrating Radar Waves in Chinese Coals"

Authors: Duo Zhang, Rui Tang, Hu Wen, Shixing Fan

**Editor**	
**Comments to the Author**	**Response**
Thank you for submitting your manuscript to PLOS ONE. After careful consideration, we feel that it has merit but does not fully meet PLOS ONE's publication criteria as it currently stands. Therefore, we invite you to submit a revised version of the manuscript that addresses the points raised during the review process. We would appreciate receiving your revised manuscript by Feb 04 2020 11:59PM. Two independent reports have been collected for your submission. Both the reports are generally encouraging, but recommend major improvements.	We thank the anonymous reviewers for taking precious time to review our manuscript. We appreciate the reviewers giving us valuable comments that are helpful to improve our manuscript. Here we attempt to reply where and how we revised our manuscript in the light of reviewers' comments. Revised contents of our manuscript and our replies are marked in red font.

Reviewer #1

**Comments to the Author**	**Response**
The key contribution of this paper is that it reports the effects of EM wave frequencies on two fundamental parameters or properties: resistivity and dielectric permitivity for different rank coal samples. I am not quite sure one can claim the outcome/results of the lab testing of these parameter can be declared as "Law". The claim of the Ultra-wideband radar wave is also arguable as the radar wave used is not necessarily ultra-wideband but the frequency was varied from 100 Mhz – 1000 MHz. Otherwise they would not be able to obtain the parameter variations with frequencies. Based on these measurements, it also derived the EM wave attenuation and speed variations with frequencies for different rank coal samples.<br>The speed is computed by using the simplified version of the wave speed formula (3). I suggest you may also compute with a more accurate formula such as (Zhou and Fullagar, 2001):<br><br>So that it may get different values to the simplified one. Surely the results may do not change much as the frequencies used are	Thanks for the reviewer's comments. In the revised manuscript, we have modified the title as well as removed the word of "Law". The controversial term UWB to GPR. As shown in Figure 1, we have developed a prototype radar device that can penetrate the coal seam to detect life information. During the research, we tested the dielectric constant of coal and analyzed the propagation of radar waves in the coal. At the initial stage of research, we have assumed some basic physical models. Experts from the China National Key R & D Project team tested our radar prototype at Jining No. 2 Coal Mine. The results show that the radar can penetrate 5m coal seam.<br>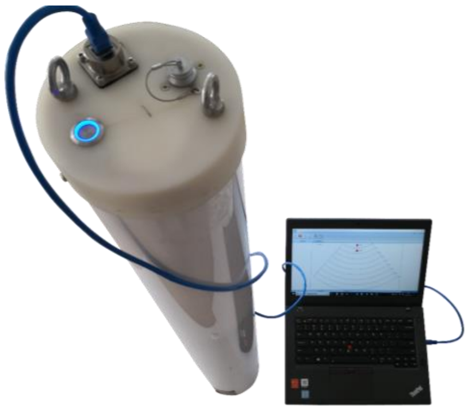<br>Fig. 1 Radar prototype for penetration detection

relatively high and the approximated version could provide sufficient accuracy.<br>One of the objectives of the paper is to use GPR for detection of mine trapped personnel after a coal mine disaster. With a frequency ranging from 100 MHz to 1000 MHz, I wonder what kind of penetration capability it will be although the paper claims, in Introduction, the GPR has "strong penetration". The detection examples in Figures 11 & 12 are simplified too much with no practical use at all. At most, you only illustrate you can estimate the distances of reflection objects without knowing which direction they come from. Such ability is well illustrated in seismic and GPR textbooks. GPR is relatively matured technique. Is there any successful examples such as from controlled experiments in coal mine environment? I guess not. Even if you can receive the reflections from trapped personnel, you need to address the issue such as how to differentiate the human reflections from many other reflections from imhomogeinities of the strata, underground equipment and objects. If you cannot resolve this issue, your GPR measurements have no use to this particular applications.<br>Based on my above comments, I suggest the author concentrated on the property measurement report rather than try also to address	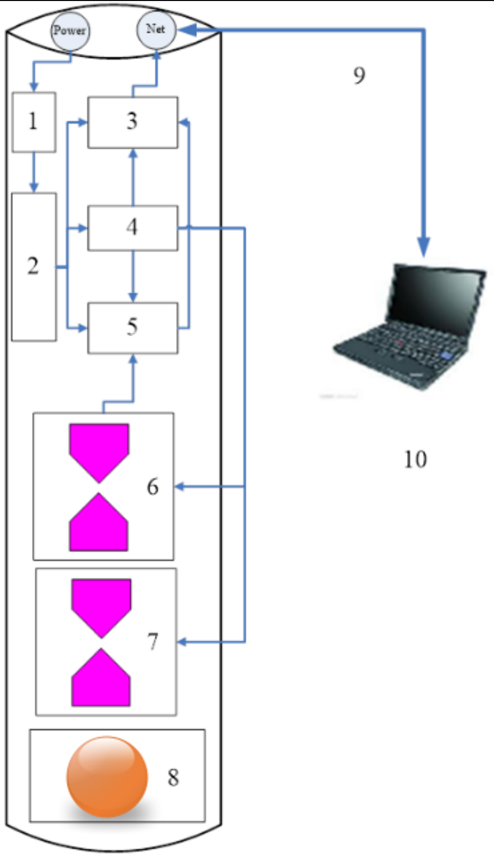<br>Fig. 2 Schematic of radar structure<br>1: battery; 2: Control module of power; 3: Data transmission module;<br>4: CPU; 5: Data acquisition module; 6: Receiving antenna;<br>7: Transmitting antenna; 8: Gyro; 9: Transmission line; 10: Computer<br>We use a combination of the delay band intersection algorithm and the

the trapped human detection. Properly understanding the property of coal samples for different frequencies is a good contribution to the literature.<br><br>In addition, please improve your English expression of the paper to ensure communicate your finding to your reader effectively. This can be achieved by asking a native English speaker or English Editing services to help you before submit your paper.	gyroscope at the bottom of the radar to determine the direction of the target. The electrical parameters of strata and human beings are different. Also the directions of reflected waves are different. Therefore, it is possible to distinguish between reflected waves of people and strata.<br>Underground equipments and objects bring enormous interference waves during the detection process. We use the following method to screen effective waves. FIR low-pass filter is made use of eliminating direct waves and some background noises. The adaptive minimum mean square algorithm is used to eliminate periodic interference waves and narrowband waves in order to improve the signal-to-noise ratio.<br>The speed formula was simplified from the accurate formula suggested by the reviewer. We find that the difference between the simplified formula and theaccurate formula was very small. To the certain accuracy, the difference between the results of the two formulas can no longer be seen. For the rigor of the manuscript, we decided to accept the reviewer's opinion that the exact formula was chosen.<br>The article has been edited by academic editing with the native language.

Reviewer #2	
**Comments to the Author**	**Response**
General remarks: The paper presents data from laboratory testing of electrical properties of various coal samples taken from Chinese mines. Test were performed on a commercial Broadband Dielectric Spectrum Test System -Concept-80. It allows to determine the values of dielectric permittivity and specific resistivity of grinded coal samples subjected to the electromagnetic fields in wide frequency spectrum. On the base of laboratory data a set of approximating equations showing the change of electric properties in frequency domain ranging from 100-1000 MHz was obtained. These equations together with known from EM theory formulas for EM waves speed and attenuation were used for modeling and studying the process of radar waves propagation in a coal seam and reflection from the miners entrapped in collapsed tunnels. The modeling concept assumes that a radar transducer with dominating frequency MHz will be lowered through a vertical or inclined rescue borehole to the depth of coal seam with mining opening. It is assumed that in not collapsed part of tunnel only miner is present. Thus on the path of radar wave from transducer to a human we have only one geological interface reflecting the radar	1. We thank the reviewer carefully checked on this part. The title has been modified to Thank you very much for your detailed suggestions and comments on the manuscript. In particular, the reviewers' general remarks can be directly used as a new abstract of the paper. We have carefully revised the manuscript based on the comments. For example, Figures 1 and 2 have been removed from the paper. We have checked and corrected the words, descriptions, spelling and grammar in the draft in detail.

signals (coal-air) and one connected with human body. The human reflector can be located in various distances from the tunnel face. This factor was included in analyzed geometrical model. On the base of modeling results the authors of the paper conclude that a reliable human origin reflected radar signal can be measured when a distance between human and transducer do not exceed 5 m.<br><br>Although the concept and research data presented in the manuscript are very interesting and valuable scientifically it needs a thorough revision. There are a lot of deficiencies. Starting from the title, the next parts of manuscript (abstract, introduction and conclusions) in my opinion should be completely re written (my general remarks can be used as a new abstract in the paper). The rest of them need to be corrected. I suggest to remove figures 1 and 2 from the paper. They do not bring nothing and are detached from the content of the paper. Some other figures also need corrections. I recommend to send the manuscript back to authors for major revision. My detailed remarks are included in a word processor file, attached to the revision. I hope that they help the authors to correct the manuscript	
