# Supplementary material for: Propagation of ground penetrating radar waves in Chinese coals
Source: PLoS One. 2020 May 21;15(5):e0233434. doi: 10.1371/journal.pone.0233434 (PMC7241710; doi:10.1371/journal.pone.0233434)
Supplement: Attachment — Submitted filename: R2 Comments.pdf [file pone.0233434.s009.pdf]

Review Comments PONE-D-19-29325-R2: “Propagation of Ground Penetrating Radar Waves in Chinese Coals” by Duo Zhang, Rui Tang, Hu Wen and Shixing Fan

I don’t think the authors have addressed my previous comments properly.

The authors replied

1> *We can distinguish the return wave is reflected by the human or the formation water. There are two reasons for this. The first is that the electrical parameters of water are significantly different from the electrical parameters of the human body. The second is that the human's reflected waves are loaded with regular heartbeat signals.*

Theoretically what you said is correct. However, in practice, how do you differentiate the human reflection from the formation water body reflection from recorded radargrams? Please provide your method in the paper. Can the radar record show or detect human’s heartbeats? What is the resolution of the GPR system? Can you please provide any evidence or references on how your GPR can detect heartbeating? Please write your paper more scientifically, not with statements without support.

2> *As the reviewer said, our radar system is a directional system. As shown in Figure 1, the radar structure includes the transmit and receive window. The size of the window is 70 mm × 50 mm. Experts from the China National Key R & D Project team tested our radar prototype at Jining No. 2 Coal Mine. One of the test scenarios is shown in Figure 2, and the test results are shown in Figure 3.*

You only provide numerical examples which prove nothing as any different objects can cause radar reflections theoretically. Your Figure 3 in your reply also shows nothing – why are there no actual radar data presented to support your conclusion? How good is the directionality of your radar system? What is the radiation pattern? Why don’t you include a real data example in this paper for human detection or event to see the heartbeating?

3> *The article has been edited by academic editing with the native language. We have provided the certificate of english editing in the previous reply. If required, we can invite academic editors to make it more standard. After reading the reviewers' comments, we all think that it is very perfect. It is more suitable as an abstract for a dissertation. Therefore, we have completely used the reviewer's comments as a new abstract.*

I have made some suggestions in the last review but you totally ignored my suggestions and believe that your English is fine! You may pay some attention to the following comments/suggestions (I am sure there are more in the paper) :

1. Line 29: Prefer to change “the authors of the paper conclude” to “, it is concluded”. The reviewer’s writing is ok, but I don’t think the authors of the paper should use similar expression.
2. Line 31: Not quite sure what exact you mean by “do not exceed 5-8 m”! Do you mean it should not exceed 5m or you really mean it should not exceed 8m. Or you mean the distances between 5m and 8m are ok. How did you derive these number? Or is this because you only modelled the data up to 8m?

3. Line 43: I am not sure you have used "particularly significant" and "major" correctly for these events – please check your original Chinese meaning with the corresponding English.
4. Line 67/8: "Fan and Jia et al." -> "Fan et al."
5. Line 69: "Fan and Chang et al." -> "Fan et al."
6. Line 82: "Peng" -> "Peng et al."
7. Line 85: "Xu" -> "Xu et al."
8. Line 88: "Emslie et al." -> "Emslie and Lagace"
9. Line 94: "Luo and Stolarczyk et al." -> "Luo et al."
10. Line 106: "*Dielectric properties measurement" ->"Dielectric property measurement"*
11. Line 108-110: Change "in" to "from".
12. Line 130: "followed similar regularities" -> "follow a similar trend". Using "regularity" is very Chinese!
13. Line 139: "larger" -> "higher". The constant is "higher" not "larger".
14. Line 160: "large" -> "high". We normally say the attenuation is high or low not large or small in English!
15. Line 184, 188, 191: "large"->"high"; "small" ->"low".
