# Supplementary material for: Propagation of ground penetrating radar waves in Chinese coals
Source: PLoS One. 2020 May 21;15(5):e0233434. doi: 10.1371/journal.pone.0233434 (PMC7241710; doi:10.1371/journal.pone.0233434)
Supplement: Attachment — Submitted filename: PONE-D-19-29325_reviewer-ak7.docx [file pone.0233434.s004.docx]

PLOS ONE

**Propagation Law of Ultra-wideband Radar Waves in Coal**

--Manuscript Draft--

| **Manuscript Number:** | PONE-D-19-29325 |
| --- | --- |
| **Article Type:** | Research Article |
| **Full Title:** | Propagation Law of Ultra-wideband Radar Waves in Coal |
| **Short Title:** | Propagation Law of Ultra-wideband Radar Waves in Coal |
| **Corresponding Author:** | Duo Zhang, Ph.D.  Xi'an University of Science and Technology Xi'an, Shaanxi CHINA |
| **Keywords:** | Coal; Dielectric characterization; UWB; Relative dielectric constant; Specific resistivity; Propagation law |
| **Abstract:** | In mine disasters, once the escape way is blocked, it is hard to locate trapped miners ~~trapped~~. Ultra-wideband (UWB) borehole radar could be one of the effective techniques ~~to solve this problem of penetrating coal seam detection~~ for remote location of miners. . Therefore, the dielectric ~~characterization~~ properties of coal and the propagation ~~law~~ of UWB radar waves in coal were studied in this paper. The dielectric properties of five different ~~metamorphic~~ types of coal~~s~~ ~~in~~ from China are investigated in ~~UWB waves~~ frequency range from 100 MHz to 1000 MHz for the first time, ~~,~~ to the best of our knowledge. In this frequency range, the relative dielectric constant and specific resistivity of coal samples were obtained ~~by~~ using the alternating current impedance method. It was found that the relative dielectric constant of coals decreased first and then increased with increasing frequency from 100 MHz to 1000 MHz. At the same frequency, the order of values of relative permittivity of coal from high to low was lean coal, lignite, gas coal, long flame coal and anthracite. The specific resistivity of coal decreases considerably with increasing frequency, but ~~there~~ ~~was~~ an upward bump ~~around~~ at frequency 500 MHz is observed. Key parameters, such as velocity of the UWB radar wave propagation in coal, were analyzed. A set of equations describing the propagation of UWB radar waves in coal was established. The above equations were analyzed ~~by~~ using GprMax software, which is based on the Finite-Difference Time-Domain (FDTD) algorithm. It was found that the effective penetration distance of UWB radar wave with dominant frequency 500 MHz in coal is between 6.0 m and 8.0 m. |
| **Order of Authors:** | Duo Zhang, Ph.D. |
|  | Rui Tang |
|  | Hu Wen |
|  | Shixing Fan |
| **Additional Information:** |  |
| **Question** | **Response** |
| **Financial Disclosure**  Enter a financial disclosure statement that describes the sources of funding for the work included in this submission. Review the [submission guidelines](http://journals.plos.org/plosone/s/submission-guidelines#loc-financial-disclosure-statement) for detailed requirements. View published research articles from [*PLOS ONE*](http://journals.plos.org/plosone/) for specific examples.  This statement is required for submission and **will appear in the published article** if the submission is accepted. Please make sure it is accurate. | Funding: The project was supported by the National Natural Science Foundation of China (grant numbers 51904234 and 51974240) and the Key R & D Program of Shaanxi Provincial (grant number 2017ZDCXL-GY-01-02-03). |

*Powered by Editorial Manager® and ProduXion Manager® from Aries Systems Corporation*

| **Unfunded studies**  Enter: *The author(s) received no specific funding for this work.*  **Funded studies**  Enter a statement with the following details:   - Initials of the authors who received each award - Grant numbers awarded to each author - The full name of each funder - URL of each funder website - Did the sponsors or funders play any role in the study design, data collection and analysis, decision to publish, or preparation of the manuscript? - **NO** - Include this sentence at the end of your statement: *The funders had no role in study design, data collection and analysis, decision to publish, or preparation of the manuscript.* - **YES** - Specify the role(s) played. |  |
| --- | --- |
| * typeset |  |
| **Competing Interests**  Use the instructions below to enter a competing interest statement for this submission. On behalf of all authors, disclose any [competing interests](http://journals.plos.org/plosone/s/competing-interests) that could be perceived to bias this work—acknowledging all financial support and any other relevant financial or non- financial competing interests.  This statement **will appear in the published article** if the submission is accepted. Please make sure it is accurate. View published research articles from [*PLOS ONE*](http://journals.plos.org/plosone/) for specific examples. | The authors have declared that no competing interests exist. |

*Powered by Editorial Manager® and ProduXion Manager® from Aries Systems Corporation*

| **NO authors have competing interests**  Enter: *The authors have declared that no competing interests exist.*  **Authors with competing interests**  Enter competing interest details beginning with this statement:  *I have read the journal's policy and the authors of this manuscript have the following competing interests: [insert competing interests here]* |  |
| --- | --- |
| * typeset |  |
| **Ethics Statement**  Enter an ethics statement for this submission. This statement is required if the study involved:   - Human participants - Human specimens or tissue - Vertebrate animals or cephalopods - Vertebrate embryos or tissues - Field research   Write "N/A" if the submission does not require an ethics statement.  General guidance is provided below. Consult the [submission guidelines](http://journals.plos.org/plosone/s/submission-guidelines#loc-guidelines-for-specific-study-types) for detailed instructions. **Make sure that all information entered here is included in the Methods section of the manuscript.** | N / A |

*Powered by Editorial Manager® and ProduXion Manager® from Aries Systems Corporation*

| **Format for specific study types**  **Human Subject Research (involving human participants and/or tissue)**   - Give the name of the institutional review board or ethics committee that approved the study - Include the approval number and/or a statement indicating approval of this research - Indicate the form of consent obtained (written/oral) or the reason that consent was not obtained (e.g. the data were analyzed anonymously)   **Animal Research (involving vertebrate animals, embryos or tissues)**   - Provide the name of the Institutional Animal Care and Use Committee (IACUC) or other relevant ethics board that reviewed the study protocol, and indicate whether they approved this research or granted a formal waiver of ethical approval - Include an approval number if one was obtained - If the study involved *non-human primates*, add *additional details* about animal welfare and steps taken to ameliorate suffering - If anesthesia, euthanasia, or any kind of animal sacrifice is part of the study, include briefly which substances and/or methods were applied   **Field Research**  Include the following details if this study involves the collection of plant, animal, or other materials from a natural setting:   - Field permit number - Name of the institution or relevant body that granted permission |  |
| --- | --- |
|  |  |
| **Data Availability**  Authors are required to make all data underlying the findings described fully available, without restriction, and from the time of publication. PLOS allows rare exceptions to address legal and ethical concerns. See the [PLOS Data Policy](http://journals.plos.org/plosone/s/data-availability) and [FAQ](http://journals.plos.org/plosone/s/data-availability#loc-faqs-for-data-policy) for detailed information. | Yes - all data are fully available without restriction |

*Powered by Editorial Manager® and ProduXion Manager® from Aries Systems Corporation*

| A Data Availability Statement describing where the data can be found is required at submission. Your answers to this question constitute the Data Availability Statement and **will be published in the article**, if accepted. |  |
| --- | --- |
| **Important:** Stating ‘data available on request from the author’ is not sufficient. If your data are only available upon request, select ‘No’ for the first question and explain your exceptional situation in the text box. |  |
| Do the authors confirm that all data underlying the findings described in their manuscript are fully available without restriction? |  |
| **Describe where the data may be found in full sentences. If you are copying our sample text, replace any instances of XXX with the appropriate details.**   - If the data are **held or will be held in a public repository**, include URLs, accession numbers or DOIs. If this information will only be available after acceptance, indicate this by ticking the box below. For example: *All XXX files are available from the XXX database (accession number(s) XXX, XXX.*). - If the data are all contained **within the manuscript and/or Supporting Information files**, enter the following: *All relevant data are within the manuscript and its Supporting Information files.* - If neither of these applies but you are able to provide **details of access elsewhere**, with or without limitations, please do so. For example:   *Data cannot be shared publicly because of [XXX]. Data are available from the XXX Institutional Data Access / Ethics Committee (contact via XXX) for researchers who meet the criteria for access to confidential data.*  *The data underlying the results presented in the study are available from (include the name of the third party* | All relevant data are within the manuscript and its Supporting Information files |

*Powered by Editorial Manager® and ProduXion Manager® from Aries Systems Corporation*

| *and contact information or URL).*   - This text is appropriate if the data are owned by a third party and authors do not have permission to share the data.   * typeset |  |
| --- | --- |
| Additional data availability information: | Tick here if the URLs/accession numbers/DOIs will be available only after acceptance of the manuscript for publication so that we can ensure their inclusion before publication. |

*Powered by Editorial Manager® and ProduXion Manager® from Aries Systems Corporation*

Manuscript	[Click here to download Manuscript Manuscript.pdf](https://www.editorialmanager.com/pone/download.aspx?id=25369578&amp;guid=de106e56-bb4d-4aab-982b-548f9e237cbf&amp;scheme=1)

1 Propagation ~~Law~~ of ~~Ultra-wideband~~ Ground Penetrating Radar Waves in Chinese Coals
2 Duo Zhang ^a,^ ^b,^ ^c,^ ^*^, Rui Tang ^a,^ ^b^, Hu Wen ^a,^ ^b^, Shixing Fan ^a,^ ^b^
3 a School of Safety Science and Engineering, Xi’an University of Science and Technology, Xi'an
4 Shaanxi 710054, China
5 b Key Laboratory of Mine and Disaster Prevention and Control of Ministry of Education, Xi'an
6 University of Science and Technology, Xi'an Shaanxi 710054, China
7 c Postdoctoral Mobile station of Mining Engineering, Xi'an University of Science and Technology,
8 Xi’an Shaanxi 710054, China
9 ^*^Corresponding author: Duo Zhang. E-mail addresses: [zhangd@xust.edu.cn](mailto:zhangd@xust.edu.cn)

10

11 Abstract: In coal mine disasters, once the escape way is blocked, it is hard to locate
12 trapped miners ~~trapped~~. The Ultra-wideband (UWB) radar could be one of the effective
13 techniques to solve this problem, ~~of penetrating coal seam detection~~. Therefore, the
14 dielectric characterization of coal and the propagation ~~law~~ of UWB radio~~ar~~ waves in coal
15 ~~were~~ are studied in this paper. The dielectric properties of five different ~~metamorphic~~
16 coals ~~in~~ from China are investigated ~~in~~ using UWB radio waves , with frequencies from 100 MHz to
17 1000 MHz for the first time, to the best of our knowledge. In these frequency range,
18 the values of relative dielectric constant and specific resistivity of coal samples were ~~obtained~~
19 measured ~~by~~ using the alternating current impedance method. It was found that the values of the relative
20 relative dielectric constant of coal~~s~~ samples ~~decreased first and then increased with increasing~~
21 ~~frequency from 100 MHz to 1000 MHz.~~ At ~~the same frequency~~ these range, the values of relative
22 dielectric permittivity depend on coal rank and decrease in following order: lean coal, lignite, gas coal, long flame coal
23 and anthracite. The specific resistivity of various coals decreases considerably with increasing
24 values of frequency, but in our tests ~~there was~~ we have observed an ~~upward~~ irregular bump ~~around~~ at frequency 500 MHz. Key parameters, such as
25 velocity of the ~~UWB~~ radar wave propagation in coal, were analyzed. A	set of
26 analytical equations describing the propagation of ~~UWB~~ radar waves in coal was established. The above
27 equations	were	analyzed	by using GprMax	software, which	is	based	on the
28 Finite-Difference Time-Domain (FDTD) algorithm. It was found that the effective
29 penetration distance of ~~UWB~~ radar waves in coal is between 6.0 m and 8.0 m.
30 Keywords: coal, dielectric permittivity specific resistivity, radar wave, propagation ~~UWB~~, ~~Relative dielectric constant,~~
31 1. Introduction
32 In China, coal is still main energy source for the foreseeable future (as shown in
33 Fig. 1) [1, 2]. In addition, it also accounts for a large proportion of the world's primary
34 energy consumption (as shown in Fig. 2) [3-5]. Therefore, safety issues in coal mining
35 processes have always been the focus and hotspot of research. Coal mine emergency
36 rescue is the last barrier to safety and security.
37 In recent years, with the ~~improvement~~ in science, technology, management, and
38 quality of workers, the safety of coal mines has obviously improved. The total number
39 of accidents and death tolls have dropped significantly. However, major accidents in
40 coal mines still occur from time to time. For example, in 2016, there were two
41 particularly significant coal mine accidents and eight major accidents [6]. Coal mine
42 accidents cause serious casualties, as along with economic losses and adverse social
43 impacts [7]. Obviously, the sooner we knew the location and condition of the people
44 in distress, the greater the possibility that they would be rescued. ~~It~~ should consider
45 the ground rescue method when we encountered the following two situations. First
46 was that the roadway could not be used due to coal or rock fall, high temperature,
47 water, etc. Second was that the lives of rescue workers are threatened uncontrollably.
48 As of now, there have been some successful drilling rescue cases. For example, a
49 water permeable accident occurred in the Kuixi Coal Mine in USA in July 2002. A
50 mine collapse accident occurred in San Jose copper mine in Chile in August 2010 [8].
51 A mine collapse accident occurred in Yurong gypsum mine in China in December
52 2015 [9]. Long-distance drilling might be deflected during construction under the
53 current technical conditions (as shown in Fig. 3). Under these circumstances, life
54 information detection technologies, such as video or audio, infrared, and gas, would
55 not be available because they cannot penetrate the coal seam [10]. Among numerous
56 non-contact life information detection technologies, UWB radar was considered to be
57 the most effective technology due to its high resolution, ~~strong~~ penetration, and low
58 power radiation, which is suitable for the life ~~detection~~ rescue systems [11, 12]. It has been used
59 in life detection and rescue [13]. Many works have been conducted on life detection
60 based on radar [14-18]. Therefore, we believed that UWB radar technology might be
61 the future development direction of life information detection through coal seams.
62 This study showed that the propagation ~~law~~ of UWB radar waves in coal, and the
63 related influencing factors have very important scientific and practical significance.
64 Numerous scholars have conducted extensive research on the electromagnetic
65 properties of coal and the propagation of electromagnetic waves in coal. Fan and Jia
66 et al., studied dielectric properties of coals in the low-terahertz frequency region [19].
67 Fan and Chang et al. studied the dielectric properties of coal in the terahertz frequency
68 region of 100–500 GHz [20]. Qin and Wei researched the relative dielectric constant
69 sweep measurement based on a vector network analyzer [21]. Brach studied the real
70 part of the permittivity of a series of coals as a function of frequency (100
71 Hz–1.3×10^7^ Hz) and temperature (200–400 K), and the results showed that certain
72 parameters are related to the microstructure of the carbonaceous phase [22]. Marland
73 et al., tested the dielectric properties of coal using the microwave cavity method in the
74 laboratory, and qualitatively discussed the effects of temperature, frequency, coal rank,
75 minerals and water content on the coal dielectric properties [23]. Valentina studied the influence
76 of polyethylene terephthalate on the carbonization of bituminous coals and on the
77 modification of their electric and dielectric properties [24]. The relationship between
78 interior moisture distribution and coal dielectric properties has been constructed by
79 Wang. The characteristics of permittivity variation with moisture content has also been
80 studied [25]. Peng studied the dielectric characteristics of an Indonesian low-rank coal
81 based on determination of its permittivity from 21 ^o^C to approximately 1000 ^o^C at 915
82 and 2450 MHz in argon [26]. Tao et al., analyzed the effect of sulfur content on the
83 dielectric properties of coal [27]. Xu studied the influence of the crystallite structure
84 on the dielectric properties of coal chars [28]. Liu studied the effect of coal rank on
85 the dielectric properties through structural differences in various coal chars [29]. Emslie
86 et al., put forward the model of a coal seam waveguide, for the first time, when
87 studying radio wave communication in coal mines [30]. Hill derived the theoretical
88 formula of electromagnetic wave propagation under the layered model and studied the
89 propagation of electromagnetic waves in uneven coal seams [31, 32]. Greenfield used
90 finite	difference	simulations	to	analyze	the	attenuation	characteristics	of
91 electromagnetic waves in discontinuous coal seams [33]. Luo and Stolarczyk et al.
92 analyzed ~~the theory on~~ the effect of electrical ~~changes~~ properties of coal seams on the attenuation
93 characteristics of electromagnetic waves [34-37]. Zhu et al. reviewed the research
94 progress on the measurement technique of coal dielectric constants and analyzed the
95 dielectric models of several coals [38]. All of the above studies have achieved fruitful
96 results, but rarely investigated the electrical parameters of different ranks of coal in
97 the frequency range of 100 MHz–1000 MHz.
98 In this paper, we report an experimental study on the dielectric properties of five
99 coal samples of different ranks, with frequencies ranging from 100 MHz to 1000 MHz.
100 The propagation control equation of ~~UWB~~ radar waves in coal is constructed, and ~~its~~
101 the propagation process ~~was~~ is simulated and solved. The effective ~~propagation~~ distance
102 range of ~~UWB~~ radar waves penetration in coal was given.
103 2. Experiment and methods
104 *2.1. Dielectric properties measurement*
105 In this study, five kinds of coal samples were used, according to the rank of coal,
106 namely, lignite (1#), in the Dananhu Coal Mine of Xinjiang; long flame coal (2#), in
107 the Tangjiahui Coal Mine of Inner Mongolia; gas coal (3#), in the Baodian Coal Mine of
108 Shandong; lean coal (4#), in the Sangshuping Coal Mine of Shaanxi; anthracite (5#), in
109 the Huangyanhui Coal Mine of Shanxi. The proximate analysis and elemental analysis
110 results are shown in Table 1. The fresh coal samples that were not subjected to water
111 injection, spraying, and other treatments were sealed and packed with multilayer
112 plastic and nylon bags, and then transported to the laboratory. Each experiment was
113 performed four times to ensure test reproducibility. The samples were prepared as
114 follows. A fresh coal sample was crushed, and pulverized coal whose diameter was
115 less than 0.074 mm was screened out. A certain amount of pulverized coal was
116 weighed into a tableting machine to prepare a circular coal sample piece having a
117 diameter of 1.28 cm and a thickness of 1.0 mm. The conductive silver colloid was
118 evenly coated on both sides of the coal sample, and could be tested after the silver gel
119 was dried. The preparation process of the sample is shown in Fig. 4.
120 The experimental equipment was a Concept-80 broadband dielectric spectrum
121 test system, which mainly included control software, an E4991A impedance analyzer
122 and test fixture, as shown in Fig. 5.
123 *2.2. The Propagation ~~Law of Ultra-wideband~~ of Radar Waves in Coal*
124 *2.2.1. Dielectric properties of tested coal samples*
125 The relative dielectric constant values of the five ~~metamorphic~~ tested coals are shown
126 in Fig. 6, and resistivity values are shown in Fig. 7.
127 It can be seen from Fig. 6 that the relative dielectric constants of five different
128 ~~metamorphic~~ coals followed similar ~~laws~~ regularities. They decreased first and then increased
129 with increasing test frequency. The minimum values of dielectric constant appeared around 300 MHz, while
130 the largest one were near 1000 MHz. At the same test frequency, lean coal had the
131 highest relative dielectric constant, followed by lignite, gas coal, long flame coal, and
132 finally anthracite, as shown in Fig. 6(F). The lignite contained a large number of
133 hydrophilic polar functional groups, which could adsorb a large amount of intrinsic
134 moisture, while polar functional groups and moisture had a high relative dielectric
135 constant. Therefore, the dielectric constant of lignite was higher. The order of relative
136 dielectric constants of the other four ranks of coal should be determined by the carbon
137 content ~~of~~ in coal. The relative dielectric constant of lean coal was larger than that of
138 lignite because it was influenced by other factors, including porosity [22]. The
139 relative dielectric constants and frequencies were fitted and were found to satisfy the
140 cubic polynomial relationship. The results are shown in Eq. (1), and correlation

coefficients are shown in Table 2.

*ε* =*C*3 *f* 3 + *C*2 *f* 2 + *C*1 *f* + *C*0 ,( *f* ∈[100*MHz*,1000*MHz*])

(1)

143 Where ε is the relative dielectric permittivity, *f* is frequency, and *C_i_* is a coefficient

145	(*i*=0, 1, 2, 3).

146 It could be seen from Fig. 7 that the resistivity of coal with a different degree of
147 metamorphism had an overall decreasing trend with an increase in test frequency.
148 There was a convex transformation point near 500 MHz, which could divide the trend
149 of resistivity with frequency into two stages. It was found, through fitting, that part Ⅰ
150 conformed to a cubic polynomial and part Ⅱ conformed to a quadratic polynomial.
151 The results were shown in Eq. (2) and Table 3. After 700 MHz, the resistivity of
152 ~~Various~~ tested coals tended to be flat and the differences ~~is~~ between them are small. As the test frequency
153 increased, electrons a in bundled state of the coal structure group might be converted
154 into freely excited electrons. This caused a significant increase in free radicals (i.e.,
155 unpaired electrons) of coals, so the resistivity would decrease. Previous studies have
156 shown that anthracite was a resistive absorbing material [39], and the resistivity of
157 mineral impurities in it was higher than that of organic matter [40]. These factors
158 might make the anthracite resistivity relatively large.

ρ ⎧*C*13 *f* 3 + *C*12 *f* 2 + *C*11 *f* + *C*10，(*f* ∈[100*MHz,*500*MHz*])

= ⎨	*C	f* 2 + *C	f* + *C* ，(*f* ∈ (500*MHz,*1000*MHz*])	(2)

⎩	22	21	20

160 *2.2.2. The equation of UWB radar wave propagation in coal*
161 To simplify the model, it was assumed that the coal body was a homogeneous
162 medium. Since	UWB	radar	waves	area	type	of	electromagnetic	wave, their
163 propagation velocity in coal could also be expressed by Eq. (3) [41]. From Eq. (3) and
164 Fig. 8, the propagation velocity of UWB radar waves in different ~~metamorphic~~ coals

could be obtained, as shown in Table 4.

*v* = *c*

*ε*

(3)

167 Where *v* is the propagation speed of UWB radar waves in coal, in m/s. *c*	is the
168 speed of light propagation in a vacuum, which is 3×10 ^8^ m/s. *ε* is the relative
169 dielectric permittivity of coal.
170 According to the literature [42], the attenuation coefficient of electromagnetic
171 wave propagation in coal could be characterized by Eq. (4). Combined with the
172 dielectric constant and electrical conductivity of coal, the variation of attenuation
173 coefficient with frequency could be obtained, as shown in Fig. 8

*a*=2*π f* ⎢

(	)^-1^⎥

1 + (1 2*π* *f ρε* )^2^

(4)

⎡*εμ*

⎣ 2

⎤1 2

⎦

175 where *α* is the attenuation coefficient of ~~UWB~~ radar wave ~~propagation~~ in coal, *f* is
176 the frequency, *ε*	is the relative dielectric permittivity of coal, and *ρ* is the
177 specific resistivity of coal.
178 It could be seen from Fig. 8 that the propagation attenuation coefficient of ~~UWB~~
179 electromagnetic waves in five ~~kinds~~ types of coal~~s~~ followed a similar ~~law~~ rule in relation to frequency. ~~It was not~~
180 ~~difficult to determine that~~ It could be divided into two parts, according to the
181 boundary line near 500 MHz. In part I, the attenuation coefficient was a cubic
182 function of frequency. It increased first and then decreased with increasing frequency.
183 At the same frequency, the attenuation coefficients of coals, from large to small, were
184 lean coal, gas coal, long flame coal, anthracite, and lignite. In part II, the attenuation
185 coefficient was a quadratic function of frequency. It increases with increasing
186 frequency. In ① of part Ⅱ, at the same frequency, the attenuation coefficient from
187 large to small was anthracite, long flame coal (gas coal, lean coal), and brown coal.
188 Among them, the attenuation coefficient of long flame coal, gas coal and lean coal
189 had little difference. In	②	of part Ⅱ , at the same frequency, the	attenuation
190 coefficient from large to small was anthracite (long flame coal, gas coal), lean coal,
191 and brown coal. Among them, the attenuation coefficient of anthracite, long flame
192 coal, and gas coal were similar.
193 The attenuation coefficient and frequency were fitted, and results were shown in

Eq. (5) and Table 5.

⎧*C'*

*f* 3 + *C' f* 2 + *C' f* + *C'* ，(*f* ∈[100*MHz,*500*MHz*])

*a*= ⎨

13	12	11	10

(5)

⎩	*C'*

22	21	20

*f* 2 + *C'*

*f* + *C'* ，(*f* ∈ (500*MHz,*1000*MHz*])

196 According to literature [43], reflection and refraction coefficients of UWB radar
197 waves propagating in coal could be characterized by Eqs. (6) and (7).

Reflection coefficient： *Rij* =

Refraction coefficient： *Tij* =

(6)

(7)

*εi* −	*ε j*

*εi* +	*ε j*

2 *εi*

*εi* + *ε j*

where

*Rij*

is the reflection coefficient of UWB radar waves at the interface between

medium *i* and medium *j*.

*Tij*

is the refraction coefficient of UWB radar waves at the

interface between medium *i* and medium *j*. *εi*

is the relative dielectric constant of

medium *i*. *ε j*

is the relative dielectric constant of medium *j*.

204 From eqs. (6) and (7), respectively, the reflection and refraction coefficients on
205 various interfaces (as shown in Fig. 9) during UWB radar wave propagation could be
206 obtained. The reflection and refraction coefficients of the interface were calculated by
207 taking the relative dielectric constant of coal at a frequency of 500 MHz as an
208 example. The results are shown in Table 6. It was not difficult to find that a negative
209 number appears in the reflection coefficient because the radar wave from the side with
210 a small relatively dielectric constant moves to the side with high relatively dielectric
211 constant, indicating that the reflected wave was opposite in phase to the incident
212 wave.
213 The propagation distance of ~~UWB~~ radar waves in coal was determined by the
214 attenuation coefficient. It could be seen from Eq. (4) that the independent variables
215 determining variable	*α*	(attenuation coefficient) are ƒ	(frequency), *ε* (relatively

dielectric constant), the *ρ* (resistivity), i.e.,

*α* =*F* ( *f* ,*ε* , *ρ* ) . As can be seen from

217 equation (1), *ε* (relatively dielectric constant) is a function of ƒ (frequency), i.e.,
218 *f* =*F* -1 (*ε* ) . From Eq. (5), it is found that ƒ (frequency) is a function of *α* (attenuation

coefficient), i.e.,

*α* =*F* -1 ( *f* ) . Thus, the propagation attenuation law of ~~UWB~~ radar

waves in coal is given by

⎧

⎪

⎪

*a*=2π*f* ⎡ ε

⎢⎣ 2

(	)

⎤1 2

-1⎥

1 + (1 2π*f* ρε)^2^

⎦

⎪	⎧*C'*

*f* 3 + *C' f* 2 + *C'*

*f* + *C'* ，(*f* ∈[100*MHz,*500*MHz*])

⎪*a*= ⎨

13	12	11	10

*C'	f* 2 + *C'	f* + *C'*	，(*f* ∈(500*MHz,*1*.*0*GHz*])

(8)

⎨	⎩	22	21	20

⎪	ε=*C*3 *f* 3 + *C*2 *f* 2 + *C*1 *f* + *C*0 *,*( *f* ∈[100*MHz,*1*.*0*GHz*])

⎪

⎪	⎧*C*13 *f* 3 + *C*12 *f* 2 + *C*11 *f* + *C*10，(*f* ∈[100*MHz,*500*MHz*])

⎪ρ= ⎨

*C f* 2 + *C f* + *C*

，(*f* ∈(500*MHz,*1*.*0*GHz*])

⎩	⎩	22	21	20

222 where *α* is the attenuation coefficient of UWB radar wave propagation in coal. *f* is

*C* ,

*ij*

the frequency. *ε* is a relative dielectric constant. *ρ* is the resistivity.

'	*Ci* ,

*Cij* ,(*i*, *j* ∈[0,1, 2,3])

are the coefficients of the corresponding function, respectively.

225 It was necessary to analyze the characteristics of waves to determine whether
226 there	was	anyone	behind	the	coal	when	using UWB	radar	for	rescue. The
227 characteristics of waves were determined by laws of refraction and reflection, that is,

Eq. (9), consisting of Eqs. (6) and (7).

⎧

⎪*Rij* =

⎪

⎨

⎪*T* =

(9)

⎪ *ij*

⎩

230

*εi* −	*ε j*

*εi* +	*ε j*

2 *εi*

*εi* + *ε j*

where *εi*

and *ε j*

are the relative dielectric constants of substance *i* and *j*,

respectively.

*Rij*

and

*Tij*

are the reflection and refraction coefficients, respectively.

232 According to the relationship between distance, speed and time, the distance
233 between the radar and person in distress can be calculated according to the following
234 formula.

⎧⎪ *L*=*v* ⋅ *t*

⎨*v* = *c*

⎩

*ε*

⎪

(10)

236 where *v* is the propagation speed of ~~UWB~~ radar waves in coal, in m/s. *c* is the
237 velocity of light propagating in vacuum, which is 3×10 ^8^ m/s.
238 The equations formed by the combination of Eqs. (8), (9), and (10) were the
239 control equations for the propagation of ~~UWB~~ radar waves in coal.

⎧	*a*=2π*f* ⎡ ε

⎪	⎢

⎪	⎣ 2

⎤1 2

-1⎥

1 + (1 2π*f* ρε)^2^

⎦

(	)

⎪	⎧*C' f* 3 + *C' f* 2 + *C'*

*f* + *C'* ，(*f* ∈[100*MHz,*500*MHz*])

⎪*a*= ⎨

13	12	11	10

⎪	⎩	*C'*

*f* 2 + *C'*

*f* + *C'*	，(*f* ∈ (500*MHz,*1*.*0*GHz*])

22	21	20

⎪	ε=*C*3 *f* 3 + *C*2 *f* 2 + *C*1 *f* + *C*0 *,*( *f* ∈[100*MHz,*1*.*0*GHz*])

⎪	⎧*C	f* 3 + *C	f* 2 + *C	f* + *C* ，(*f* ∈[100*MHz,*500*MHz*])

⎪ρ= ⎨

⎪

⎩

⎨

⎪

⎪

⎪

⎪

⎪

⎪

⎪

⎪

⎪

⎪⎩

13	12	11	10

*C*22 *f* 2 + *C*21 *f* + *C*20，(*f* ∈ (500*MHz,*1*.*0*GHz*])	(11)

ε*i*	−	ε *j*

ε*i*	+	ε *j*

2 ε*i*

ε*i* + ε *j*

*Rij* =

*Tij* =

*v* = *c L*=*v* ⋅ *t*

ε

241 3. Results and discussion
242 The equations. (11) could be solved using GprMax code. The GprMax is a simulation software based
243 on FDTD theory, which was developed by Dr. Antonis Giannopoulos of the
244 University of Edinburgh [44]. The GprMax has been widely used for simulation and modelling of radar waves propagation in such GPR applications as underground pipeline
245 surveys, roadbed inspections, ancient building surveys, military surveys, quality
246 inspections, and hidden grave surveys [45-49]. The simulation calculation process is
247 shown in Fig. 10. The simulated physical model is shown in Fig. 11(Ⅰ).
248 As shown in Fig. 11(Ⅱ), the thickness of a coal body between a trapped person
249 and radar was L_1_, the distance between a person and coal wall was L_2_, the width of the
250 model was L_3_, the length of model was L_4_, and the size of a human body is 1.6 m ×0.4
251 m. The specific settings of different models are shown in Table 7. Then, the simulated
252 data was post-processed using Matlab, and the result is shown in Fig. 11(Ⅲ).
253 It was assumed that coal was a semi-infinite continuous space with homogeneous
254 isotropy. The dielectric constant of a human was 50 [50]. The dielectric constant of air
255 was 1, the antenna stepping distance was 0.06 m, and the transmitting and receiving
256 antenna spacing was 0.065 m. The number of measuring lines was 115, the spatial
257 grid step was 0.005 m×0.005 m, the permeability of all media in the model was 1.0,
258 the boundary condition was a perfect matching layer (PML), the excitation function of
259 the radar was Ricker, and the center frequency was 500 MHz. The partial electrical
260 parameters of the model were set as shown in Table 8. Due to the large number of
261 graphs of simulation results, only lignite was taken as an example for analysis here.
262 The results are shown in Fig. 12.
263 From Fig. 12, we found that the ~~return~~ amplitudes of return wave from the interface between the
264 coal pillar and roadway in seven models were 12.51, 5.48, 2.56, 1.23, 0.63, 0.30, and
265 0.15 mV/m, and the return amplitude of the human body is -20.63. -10.24, -5.30, -2.72,
266 -1.37, -0.69, and -0.35 mV/m. It was not difficult to find that the intensity of amplitude
267 gradually decreased with the increase of L_1_ (thickness of the coal pillar). The larger
268 the L_1_, the longer the electromagnetic wave travels in coal, and the loss of
269 electromagnetic wave energy also increased, so the intensity of the target return wave
270 amplitude was gradually smaller. By fitting, it was found that the amplitude of the
271 human body wave decreases with the increase of L_1_ as an exponential function (as
272 shown in Fig. 13). The effective propagation distance of ~~UWB~~ radar waves in coal
273 was between 6 and 8 m for transducer with central frequency 500 MHz. .
274 4. Conclusions
275 Dielectric ~~characterization~~ properties of five different ~~metamorphic~~ coals from ~~in~~ Chinese deposits ~~was~~ were
276 studied	using	the	alternating	current	impedance	method	and	the Concept-80
277 broadband dielectric Spectrum test system for ~~UWB~~ electromagnetic waves with frequencies ranging
278 from 100 MHz to 1000 MHz, for the first time, to the best of our knowledge. The
279 propagation ~~law~~ of ~~UWB~~ radar waves in coal was analyzed …………..
280 The experimental results showed that the dielectric constants of five different
281 ~~metamorphic~~ coals followed similar laws. The values of relative dielectric constant of coals
282 decreased first and then ~~an~~ increased in frequency. At the same frequency, the order of
283 values of the relative permittivity of coal from high to low was lean coal, lignite, gas coal, long
284 flame coal, and anthracite. The values of specific resistivity of coal decreased considerably with
285 increasing frequency, but there was observed an upward bump around frequency 500 MHz.
286 The key parameters of ~~UWB~~ radar waves propagating in coal were analyzed. It
287 was found that the velocity, reflection and refraction coefficients were only related to
288 the relative dielectric constant of the substance. The propagation velocity values of
289 ~~UWB~~ radar waves in different rank coals and the reflection and refraction coefficients
290 values at different types of interfaces were calculated. The ~~propagation~~ attenuation
291 coefficient of ~~UWB~~ radar waves in five kinds of coals followed a similar ~~law~~.
292 With 500 MHz as the dividing point, the relation between attenuation coefficient of ~~propagation~~
293 ~~with~~ and frequency ~~variation~~ was divided into two parts. In part I, the frequency was a
294 cubic function of the attenuation coefficient. The attenuation coefficient initially increases to a certain point
295 and then decreases with increasing of frequency values. In part II, the frequency was a
296 quadratic function of the attenuation coefficient, and the values of attenuation coefficient
297 increased with frequency increase. A set of ~~Eqs.~~ equations for the propagation of ~~UWB~~ radar
298 waves in coal were established, based on the above researches. By analyzing the
299 governing equations, it was found that the effective ~~propagation~~ penetration distance of ~~UWB~~

radar wave with dominant frequency 500 MHz in coal ranges between 6 and 8 m.

302 Acknowledgements
303 Funding: The project was supported by the National Natural Science Foundation of
304 China (grant numbers 51904234 and 51974240) and the Key R & D Program of
305 Shaanxi Provincial (grant number 2017ZDCXL-GY-01-02-03).

References

1. Guo L, Zhai M, Wang Z, Zhang Y, Dong P. Comprehensive coal quality index for evaluation of coal agglomeration characteristics, Fuel 2018; 231(12): 379-386.
2. Song H, Liu J, Xue F, Cheng F. The application of ultra-fine fly ash in the seal coating for the wall of underground coal mine, Advanced Powder Technology 2016; 27: 1645-1650.
3. Diniz DA, Prasad P, Pagan RJ. Modern environmental improvement pathways for the coal power generation industry in Australia. Process Safety and Environmental Protection 2004; 82: 191-199.
4. Warmuzinski K. Harnessing methane emissions from coal mining. Process Safety and Environmental Protection 2008; 86: 315-320.
5. British Petroleum (BP), Statistical Review of World Energy, 2015. http://www.bp.

ucom/statistical review.

6. Wang D. Thermodynamic disaster in coal mine and its characteristics. Journal of China Coal Society 2018; 43(1): 137-142.
7. Wang K, Jiang S, Ma X, Wu Z, Shao H, Zhang W, et al. Information fusion of plume control and personnel escape during the emergency rescue of external-caused fire in a coal mine.

Process Safety and Environmental Protection 2016; 103: 46-59.

8. Ken B. Buried alive, but carbide drills at core of Chile miners' rescue. Metal Powder Report 2010; 65(7): 7-10.
9. Yang T, Du B. Construction Technology of Large Diameter rescue borehole in Pingyi gypsum mine disaster of Shandong, Exploration Engineering ( Rock & Soil Drilling and Tunneling) 2017, 44(5): 19-23. (In Chinese)
10. Wen H, Zhang D, Zheng X-Z. Research Progress and Trend of Life Detection Technology of Drilling and Rescue in Mine. Safety in Coal Mines 2017, 48(9): 85-88. (In Chinese)
11. Li Z, Li W, Lv H, Zhang Y, Jing X, Wang J. A novel method for respiration-like clutter cancellation in life detection by dual-frequency IR-UWB radar, IEEE Transactions on Microwave Theory and Techniques 2013; 61 (5): 2086-2092.
12. Hu W, Zhao Z, Wang Y, Zhang H, Lin F. Noncontact accurate measurement of cardiopulmonary activity using a compact quadrature Doppler radar sensor, I IEEE Transactions on Biomedical Engineering 2014; 61 (3): 725-735.
13. Chen K. Huang Y, Zhang J, Norman A. Microwave life-detection systems for searching human subjects under earthquake rubble or behind barrier, IEEE Transactions on Biomedical Engineering 2000; 47 (1): 105-114.
14. Jalali Bidgoli F, Moghadami S, Ardalan S. A compact portable microwave life detection device for finding survivors. IEEE Embedded Systems Letters 2016; 8(1): 10-13.
15. Liang X, Zhang H, Fang G, Ye S, Gulliver T. An improved algorithm for through wall target detection using ultra-wideband impulse radar. IEEE Access 2017; (99): 22101-22118.
16. Guan S, Rice JA, Li C, Gu C. Automated dc offset calibration strategy for structural health monitoring based on portable cw radar sensor. IEEE Transactions on Instrumentation and Measurement 2014; 63(12): 3111-3118.
17. Wang F, Horng TS, Peng K, Jau J, Li J, Chen C. Single-antenna Doppler radars using self and mutual injection locking for vital sign detection with random body movement cancellation. IEEE Transactinos on Microwave Theory and Techniques 2011; 59 (12): 3577-3587.
18. Liang X, Zhang H, Lyu T, Xu L, Cao C, Gulliver TA. Ultra-wide band impulse radar for life

detection using wavelet packet decomposition. Physical Communication 2018; 29:31-47.

19. Fan W, Jia C-Y, Hu W, Yang C, Liu L, Zhang X, et al. Dielectric properties of coals in the low-terahertz frequency region. Fuel 2015; 162: 294-304.
20. Fan W, Chang T, Liu L, Cui H. Dielectric properties of coal in the terahertz frequency region of 100–500 GHz. Fuel 2017; 188: 246-253.
21. Qin F, Wei G. Measurement with Sweeping Frequency for Complex Permittivity Based on VNA. Journal of Astronautic Metrology and Measurement 2011; 31(3): 27-30.
22. Brach I, Giuntini JC, Zanchetta JC. Real part of the permittivity of coals and their rank. Fuel 1994; 73(5): 738-741.
23. Marland S, Merchant A., Rowson N. Dielectric properties of coal. Fuel 2001; 80: 1839-1849.
24. Valentina VZ. Influence of polyethylene terephthalate on the carbonisation of bituminous coals and on the modification of their electric and dielectric properties. Fuel; 2006, 85:1652-1665.
25. Wang Q, Zhang X, Gu F. Investigation on interior moisture distribution inducing dielectric anisotropy of coals. Fuel processing technology 2005; 89:633-641.
26. Peng Z, Lin X, Li Z, Hwang J, Kim B, Zhang Y, et al. Dielectric characterization of Indonesian low-rank coal for microwave processing. Fuel processing technology 2017; 156: 171-177.
27. Tao X, Tang L, Xie M, He H, Xu N, Feng L, et al. Dielectric properties analysis of sulfur-containing models in coal and energy evaluation of their sulfur-containing bond dissociation in microwave field. Fuel 2016; 181: 1027-1033.
28. Xu L, Liu H, Jin Y, Fan B, Qiao X, Jing B. Structural order and dielectric properties of coal chars. Fuel 2014; 137: 164-171.
29. Liu H, Xu L, Jin Y, Fan B, Qiao X, Yang Y. Effect of coal rank on structure and dielectric

properties of chars. Fuel 2015; 153: 249-256.

30. Emslie AG, Lagace RL. Propagation of low and medium frequency radio waves in a coal seam.

Radio Science 1976; (11): 253-261.

31. Hill DA. Radio propagation in a coal seam and the inverse problem. Journal of Research of the National Bureau of Standards 1984; (89): 385-394.
32. Hill DA. Electromagnetic detection of long conductors in tunnels. IEEE Trans 1985; 33(4).
33. Greenfield RJ,Wu ST. Electromagnetic wave propagation in disrupted coal seams. Geophysics 1991; 56(10): 1571-1577.
34. Luo Y, Stolarczyk LG, Peng SD. Demonstration of the advanced radio imaging method (rim): ground truth results for three-dimensional imaging of coal seams. Sme Meeting 2004.
35. Stolarczyk LG, Fry RC. Radio imaging method (RIM) or diagnostic imaging of anomalous geologic structures in coal seam waveguides. Transactions of Society for Mining, Metallurgy, and Exploration, Inc. 1990; (288): 1806-1814.
36. Stolarczyk LG. Electromagnetic seam wave mapping of roof rock conditions across a longwall panel. 18th International Conference on Ground Control in Mining, Aug 3-5.1999.
37. Stolarczyk LG, Peng SS, Luo Y. Imaging ahead of Mining with Radio Imaging Method (RIM-IV) Instrumentation and Three-dimensional Tomography. Software Proceedings of 22nd International Conference on Ground Control in Mining 2003, Morgantown, WV, 136-143.
38. Zhu H, Xin M, Chang M, Liu D. Research progress on measuring technology of coal dielectric

constant. Coal Science Technology 2016; 44(9): 6-12．

39. Singclair DC, West AR. Impedance and modulus spectroscopy of semiconducting BaTiO_3_

showing positive temperature coefficient of resistance. Journal of Applied. Physics 1989; 66(8): 3850-3856.

40. Feng X, Chen J, Li N, Cui H, Liu J. Study on electromagnetic properties of anthracite and soft coal in microwave field. Study on electromagnetic properties of anthracite 2007; 38(5): 405-411. (In Chinese~~s~~)
41. Zhang D. Fundamental research on propagation law of UWB radar wave in coal and localiztion. Xi’an university of science and technology 2018. (In Chinses)
42. Qi C. Ground penetrating radar signal processing in advanced detection in coal seam, Xi’an university of science and technology 2014. (In Chinese~~s~~)
43. Liu X. Research the propagation rules of middle-high frequency electromagnetic wave in coal medium. China University of Mining and Technology 2012.
44. Giannopoulos A. Modelling ground penetrating radar by GprMax. Construction and Building Materials 2005; 19: 755-762.
45. Núñez -Nieto X, Solla M, Novob A, Lorenzo H. Three-dimensional ground-penetrating radar

methodologies for the characterization and volumetric reconstruction of underground tunneling. Construction and Building Materials 2014; 71: 551-560.

46. Adam DB, Jamie K, Pringle. Semblance analysis to assess GPR data from a five-year forensic study of simulated clandestine graves. Journal of Applied Geophysics 2016; 125: 17-44.
47. Lopera O, Milisavljevic N. Prediction of the effects of soil and target properties on the antipersonnel landmine detection performance of ground- penetrating radar: A Colombian case study. Journal of Applied Geophysics 2007; 63: 13-23.
48. Shangguan P, Imad L. AQ, Lahouar S. Pattern recognition algorithms for density estimation of

asphalt pavement during compaction: a simulation study. Journal of Applied Geophysics 2014; 107: 8-15.

49. Jaw SW, Hashim M. Locational accuracy of underground utility mapping using ground penetrating radar. Tunnelling and Underground Space Technology Incorporating Trenchless Technology Research 2013; 35: 20-29.
50. Dogaru T, Nguyen L, Le C. Computer models of the human body signature for sensing through the wall radar applications. DTIC Document Adelphi MD 2007.
51. Lee JH, Wang W. Characterization of snow cover using ground penetrating radar for vehicle trafficability–Experiments and modeling. Journal of Terramechanics 2009; 46: 189-202.

Table captions

**Table 1** Proximate and elemental analysis of coal.

**Table 2** Coefficients of fitting equation.

**Table 3** Coefficients of fitting equation.

**Table 4** Propagation speed of UWB radar waves in coal (m/s).

**Table 5** Coefficients of fitting equation. **Table 6** Reflection and refraction coefficients. **Table 7** Size of model (m).

**Table 8** Dielectric characterization of Coal.

1	**Table 1** Proximate and elemental analysis of coal.

Coal

| Proximate analysis (%) Elemental analysis (%) Porosity | | | | | | | | | | | | | | |
| --- | --- | --- | --- | --- | --- | --- | --- | --- | --- | --- | --- | --- | --- | --- |
|  |  | | Mad | Aad | Vad | FC_ad_ | C | H | O | N | S | (%) | |  |
|  | 1# | | 8.80 | 36.62 | 24.68 | 29.90 | 38.94 | 4.01 | 22.00 | 1.15 | 0.27 | 10.11 | |  |
|  | 2# | | 5.77 | 11.93 | 32.32 | 49.98 | 64.86 | 5.63 | 19.20 | 1.73 | 0.58 | 13.34 | |  |
|  | 3# | | 2.33 | 15.92 | 35.45 | 46.30 | 63.92 | 4.85 | 17.40 | 2.37 | 0.51 | 6.16 | |  |
|  | 4# | | 0.53 | 24.49 | 12.95 | 62.03 | 62.51 | 3.76 | 17.00 | 1.61 | 3.41 | 9.19 | |  |
|  | 5# | | 1.20 | 8.84 | 7.85 | 82.11 | 81.82 | 3.79 | 4.90 | 0.85 | 1.49 | 16.20 | |  |
| 2 |  | |  |  |  |  |  |  |  |  |  |  | |  |
| 3 **Table 2** Coefficients of fitting equation. | | | | | | | | | | | | | |  |
|  | | Coals | | C_3_ | | C_2_ | | C_1_ | C_0_ | | R^2^（%） | |  |  |
|  | | 1# | | -9.24×10 ^-10^ | | 1.84×10 ^-6^ | | -9.14×10 ^-4^ | 3.62 | | 98.98 | |  |  |
|  | | 2# | | -5.82×10 ^-10^ | | 1.17×10 ^-6^ | | -5.43×10 ^-4^ | 3.07 | | 98.77 | |  |  |
|  | | 3# | | -6.34×10 ^-10^ | | 1.28×10 ^-6^ | | -5.96×10 ^-4^ | 3.35 | | 99.21 | |  |  |
|  | | 4# | | -7.90×10 ^-10^ | | 1.52×10 ^-6^ | | -6.58×10 ^-4^ | 3.78 | | 99.40 | |  |  |
|  | | 5# | | -6.42×10 ^-10^ | | 1.34×10 ^-6^ | | -6.86×10 ^-4^ | 3.05 | | 98.49 | |  |  |
| 4 | |  | |  | |  | |  |  | |  | |  |  |
| 5 **Table 3** Coefficients of fitting equation. | | | | | | | | | | | | | |  |

| Partion | Coefficient | 1# | 2# | 3# | 4# | 5# |
| --- | --- | --- | --- | --- | --- | --- |
|  | C13 | -8.00×10^-5^ | -5.45×10^-5^ | -4.96×10^-5^ | -4.08×10^-5^ | -7.48×10^-5^ |
|  | C12 | 0.10 | 0.07 | 0.06 | 0.05 | 0.10 |
| Ⅰ | C11 | -43.06 | -30.19 | -27.52 | -23.03 | -40.16 |
|  | C10 | 6.13×10 ^3^ | 4.45×10 ^3^ | 4.06×10 ^3^ | 3.43×10 ^3^ | 5.78×10 ^3^ |
|  | R2 | 98.40 % | 99.25 % | 99.25 % | 99.24 % | 98.95 % |
| C22 | | 1.28×10 ^-3^ | 9.50×10 ^-4^ | 8.74×10^-4^ | 7.99×10 ^-4^ | 1.12×10^-3^ |
| C21  Ⅱ | | -2.36 | -1.81 | -1.66 | -1.48 | -2.22 |
| C20 | | 1.21×10 ^3^ | 9.60×10 ^3^ | 8.80×10^2^ | 7.86×10 ^2^ | 1.10×10 ^3^ |

R^2^	97.94 %	99.11 %	98.84 %	98.58 %	98.91 %

6

7 **Table 4** Propagation speed of UWB radar waves in coal (m/s).

| Frequency (MHz) | 1# | 2# | 3# | 4# | 5# |
| --- | --- | --- | --- | --- | --- |
| 100 | 15.70×10 ^7^ | 17.23×10 ^7^ | 16.14×10 ^7^ | 15.53×10 ^7^ | 17.32×10 ^7^ |
| 200 | 16.01×10 ^7^ | 17.29×10 ^7^ | 16.64×10 ^7^ | 15.57×10 ^7^ | 17.43×10 ^7^ |
| 300 | 16.05×10 ^7^ | 17.32×10 ^7^ | 16.90×10 ^7^ | 15.59×10 ^7^ | 17.46×10 ^7^ |
| 400 | 16.03×10 ^7^ | 17.29×10 ^7^ | 16.64×10 ^7^ | 15.55×10 ^7^ | 17.46×10 ^7^ |
| 500 | 16.01×10 ^7^ | 17.23×10 ^7^ | 16.14×10 ^7^ | 15.53×10 ^7^ | 17.39×10 ^7^ |
| 600 | 15.94×10 ^7^ | 17.20×10 ^7^ | 16.64×10 ^7^ | 15.45×10 ^7^ | 17.37×10 ^7^ |
| 700 | 15.87×10 ^7^ | 17.12×10 ^7^ | 16.15×10 ^7^ | 15.38×10 ^7^ | 17.29×10 ^7^ |
| 800 | 15.81×10 ^7^ | 17.66×10 ^7^ | 16.42×10 ^7^ | 15.32×10 ^7^ | 17.26×10 ^7^ |
| 900 | 15.76×10 ^7^ | 17.14×10 ^7^ | 16.29×10 ^7^ | 15.30×10 ^7^ | 17.17×10 ^7^ |
| 1000 | 15.74×10 ^7^ | 16.84×10 ^7^ | 16.26×10 ^7^ | 15.26×10 ^7^ | 17.14×10 ^7^ |

8 **Table 5** Coefficients of fitting equation.

| Part | Coefficient | 1# | 2# | 3# | 4# | 5# |
| --- | --- | --- | --- | --- | --- | --- |
|  | C'_13_ | -8.31×10 ^-14^ | -6.70×10 ^-14^ | -7.01×10 ^-14^ | -7.58×10 ^-14^ | -6.91×10 ^-14^ |
|  | C'_12_ | 6.36×10 ^-11^ | 4.95×10 ^-11^ | 5.17×10 ^-11^ | 5.55×10 ^-11^ | 5.21×10 ^-11^ |
| Ⅰ | C'_11_ | -1.03×10 ^-8^ | -6.20×10 ^-9^ | -6.46×10 ^-9^ | -6.90×10 ^-9^ | -7.25×10 ^-9^ |
|  | C'_10_ | 4.71×10 ^--7^ | 1.97×10 ^-7^ | 2.05×10 ^-7^ | 2.25×10 ^-7^ | 2.52×10 ^-7^ |

R^2^	99.36%	99.72%	99.72%	99.71%	99.69%

C'_22_	-3.31×10 ^-12^	-1.31×10 ^-12^	-2.22×10 ^-12^	-4.63×10 ^-12^	-6.46×10 ^-11^

C'_21_	8.44×10 ^-9^	6.78×10 ^-9^	7.90×10 ^-9^	1.04×10 ^-8^	6.12×10 ^-9^

Ⅱ

C'_20_

-2.61×10 ^-6^	2.04×10 ^-6^	-2.34×10 ^-6^	-2.94×10 ^-6^	-1.97×10 ^-6^

R^2^	98.88%	99.52%	99.37%	98.66%	99.58%

9

10	**Table 6** Reflection and refraction coefficients.

Coal	Type

(1)

air→coal

(2)

coal→air

(3)

air→person

(4)

person→air

(5)

coal→person

(6)

person→coal

| *R_ij_* -0.31 0.31 -0.75 0.75  1# | | | | | | | | -0.58 | | 0.58 | | |
| --- | --- | --- | --- | --- | --- | --- | --- | --- | --- | --- | --- | --- |
| *Tij* | | | 0.698 | 1.31 | 0.25 | | 1.75 | 0.42 | | 1.58 | | |
| *Rij*  2# | | | -0.27 | 0.27 | -0.75 | | 0.75 | -0.60 | | 0.60 | | |
| *Tij* | | | 0.28 | 1.27 | 0.25 | | 1.75 | 0.40 | | 1.60 | | |
| *Rij*  3# | | | -0.29 | 0.29 | -0.75 | | 0.75 | -0.59 | | 0.59 | | |
| *Tij* | | | 0.71 | 1.29 | 0.25 | | 1.75 | 0.41 | | 1.59 | | |
| *Rij*  4# | | | -0.32 | 0.32 | -0.75 | | 0.75 | -0.57 | | 0.57 | | |
| *Tij* | | | 0.68 | 1.32 | 0.25 | | 1.75 | 0.43 | | 1.57 | | |
| *Rij*  5# | | | -0.27 | 0.27 | -0.75 | | 0.75 | -0.61 | | 0.67 | | |
| *Tij* | | | 0.737 | 1.27 | 0.25 | | 1.75 | 0.39 | | 1.67 | | |
| 11  12 **Table 7** Size of model (m). | | | | | | | | | | | | |
|  | Model | | M1 | M2 | M3 | M4 | M5 | M6 | M7 | | |  |
|  | L_1_ | | 2 | 1.3 | 4 | 10 | 6 | 7 | 8 | | |  |
|  | L_2_ | | 3 | 1.3 | 5 | 10 | 1.3 | 1.3 | 1.3 | | |  |
|  | L_3_ | | 4 | 1.3 | 6 | 10 | 8 | 9 | 10 | | |  |
|  | L_4_ | | 5 | 1.3 | 7 | 10 | 10 | 10 | 10 | | |  |
| 13 |  | |  |  |  |  |  |  |  | | |  |
| 14 **Table 8** Dielectric characterization of Coal**.** | | | | | | | | | | | | |
|  | | Coal | | 1# | 2# | | 3# | 4# | 5# | |  | |
|  | | ε | | 3.51 | 3.03 | | 3.30 | 3.73 | 2.99 | |  | |
|  | | ρ (Ω·m) | | 328.95 | 290.07 | | 267.78 | 242.41 | 318.53 | |  | |
| 15 | |  | |  |  | |  |  |  | |  | |

Figure	[Click here to download Figure Figure captions.pdf](https://www.editorialmanager.com/pone/download.aspx?id=25369570&amp;guid=7c9366a2-feb3-4081-90e0-a14caa870d18&amp;scheme=1)

Figure captions

**Fig. 1.** Schematic diagram of China's primary energy consumption structure from 1978 to 2015.

**Fig. 2.** The composition of the top five countries' energy consumptions in 2015.

**Fig. 3.** Diagram of location migration of bottom position of drilling.

**Fig. 4.** Flow chart of processing test of coal samples.

**Fig. 5.** Concept-80 Broadband Dielectric Spectrum Test System.

**Fig. 6.** Variations of the relative dielectric permittivity with frequency for coals.

**Fig. 7.** Variations of the resistivity with frequency for coals. **Fig. 8.** Variations of attenuation coefficient with frequency. **Fig. 9.** Schematic diagram of types of reflection and refraction. **Fig. 10.** Simulation calculation process.

**Fig. 11.** Simulation model with GprMax modeling.

**Fig. 12.** Propagation law of UWB radar waves in coal.

**Fig. 13.** ~~Variations~~ Changes of amplitude of a person echo signal with thickness of coal (L_1_).

100

76.2

Coal Oil Gas

Water/Nuclear/Wind

70.7

69.2 70.8 71.1 71.1 70.3 70.4

68 68.7

66.7	66	66

64

22.7

22.2

16.6

19.8 19.3 18.8 18.3 17.9

19

18.1 18.7 18.4 17.5 18.1

3.34.4 2.1

5.1

6.4 6.8

2.2 2.6

6.7

2.9

6.8

3.3

7.7 7.8

3.7 3.9

8.6

4.4

8

5.2

9.1 9.8

5.5 5.8

10.9

5.6

12

5.9

90

80

70

Proportion (%)

60

50

40

30

20

10

0

**Fig. 1.** Schematic diagram of China's primary energy consumption structure from 1978 to 2015.

|  |  | Oil |  | Gas |  | Coal |  | Nuclear |  | water |  | Renewable energy |
| --- | --- | --- | --- | --- | --- | --- | --- | --- | --- | --- | --- | --- |

100

2.8

6.8

4.4

2.1

8.5

1.3

3.1

2.5

8.3

2.2

4

1.2

0.1

5.8

6.6

3.2

4.9

13.3

17.4

26.6

29.2

58.1

63.7

31.3

22.8

52.8

23.8

6.5

5.9

90

0.2

80

70

Proportion (%)

60

50

40

30

20

10

0

World Total	PRC	U.S.A	India	Russia	Japan

**Fig. 2.** The composition of the top five countries' energy consumptions in 2015.

**Fig. 3** Diagram of location migration of bottom position of drilling.

A is a three-dimensional diagram of drilling rescue during disaster；B is a schematic diagram of

the drilling offset.

**Tableting**

**machine**

**Slice of coal**

**Fig. 4** Flow chart of processing test of coal samples.

**Fig. 5.** Concept-80 Broadband Dielectric Spectrum Test System.

3.64	3.14

2#

Fitting line

1#

Fitting line

3.62	3.12

3.60	3.10

Relative dielctric permittivity

Relative dielctric permittivity

3.58	3.08

3.56	3.06

3.54	3.04

3.52	3.02

3.50

3.48

(A)

200	400	600	800	1000

Frequency (MHz)

3.00

2.98

(B)

200	400	600	800	1000

Frequency (MHz)

3.42

3.40

3.38

Relative dielctric permittivity

3.36

3.34

3.32

3.30

3.28

3.26

3.24

(C)

3#

Fitting line

3.88

3.86

3.84

Relative dielctric permittivity

3.82

3.80

3.78

3.76

3.74

3.72

3.70

3.68

4#

Fitting line

(D)

200	400	600	800	1000

Frequency (MHz)

200	400	600	800	1000

Frequency (MHz)

3.08

5#

Fitting line

3.06

3.04

Relative dielctric permittivity

3.02

3.00

2.98

3.9

3.8

3#

1#

2#

3.7

4#

5#

Relative dielctric permittivity

3.6

3.5

3.4

3.3

3.2

2.96

2.94

(E)

200	400	600	800	1000

Frequency (MHz)

3.1

3.0

2.9

(F)

200	400	600	800	1000	1200

Frequency (MHz)

**Fig. 6.** ~~Variations~~ Changes of the relative dielectric permittivity with frequency for various coals

1400

1200

1000

Resistivity (Ω*m)

800

600

400

200

(**A**)

y=-8.00×10 ^-5^x^3^+0.10x^2^-43.06x+6.13×10 3

R^2^=98.40%

y=1.28×10 ^-3^x^2^-2.36x+1.21×10 3

R^2^=97.94%

1000

750

Resistivity (Ω*m)

500

250

(**B**)

y=-5.45×10 ^-5^x^3^+0.07x^2^-30.19x+4.45×10 ^3^

R^2^=99.25%

y=9.50×10 ^-4^x^2^-1.81x+9.60×10 ^2^

R^2^=99.11%

Part Ⅰ

0

Part Ⅱ

Part Ⅰ

0

Part Ⅱ

200	400	600	800	1000

Frequency (108 Hz)

200	400	600	800	1000

Frequency (MHz)

900

800

700

600

Resistivity (Ω*m)

500

400

300

200

(**C**)

y=-4.96×10 ^-5^x^3^+0.06x^2^-27.52x+4.06×10 ^3^

R^2^=99.25%

y=8.74×10 ^-4^x^2^-1.66x+8.80×10 ^2^

R^2^=98.84%

800

700

600

500

Resistivity (Ω*m)

400

300

200

(**D**)

y=-4.08×10 ^-5^x^3^+0.05x^2^-23.03x+3.43×10 ^3^

R^2^=99.24%

y=7.99×10 ^-4^x^2^-1.48x+7.86×10 ^2^

R^2^=98.58%

100

Part Ⅰ

Part Ⅱ

100

Part Ⅰ

Part Ⅱ

0

200	400	600	800	1000

Frequency (10^8^ Hz)

200	400	600	800	1000

Frequency (MHz)

1200

1000

Resistivity (Ω*m)

800

(**E**)

y=-7.48×10 ^-5^x^3^+0.10x^2^-40.16x+5.78×10 ^3^

R^2^=98.95%

1200

1000

Resistivity (Ω*m)

800

(**F**)	1#

2#

3#

4#

5#

600

400

200

0

Part Ⅰ

y=1.12×10 ^-3^x^2^-2.22x+1.10×10 ^3^

R^2^=98.91%

Part Ⅱ

600

400

200

0

Part Ⅰ

Part Ⅱ

200	400	600	800	1000

Frequency (MHz)

200	400	600	800	1000

Frequency (MHz)

**Fig. 7.** ~~Variations~~ Changes of various coals resistivity with frequency. ~~for.~~

2.5x10^-6^

Attenuation coefficient (Np/m)

2.0x10^-6^

(**A**)

3.5x10^-6^	(**B**)

3.0x10^-6^

Attenuation coefficient (Np/m)

2.5x10^-6^

1.5x10^-6^

-14 3

-11	2	-8	-7

y=-3.31e^-12^x^2^+8.44e^-9^x-2.61e^-6^

2.0x10^-6^

y=-6.7e^-14^x^3^+4.95e^-11^x^2^-6.2e^-9^x+1.97e^-7^

y=-8.31e x +6.36e x -1.03e x+4.71e

2

R^2^=98.88%

R^2^=99.72%

-12	2	-9	-6

1.0x10^-6^

5.0x10^-7^

0.0

R =99.36%

PartⅠ

Part Ⅱ

1.5x10^-6^

1.0x10^-6^

5.0x10^-7^

0.0

-5.0x10^-7^

PartⅠ

y=-1.31e x +6.78e x+2.04e R^2^=99.52%

Part Ⅱ

0	200	400	600	800	1000

Frequency (MHz)

3.5x10^-6^	(**C**)

3.0x10^-6^

Attenuation coefficient (Np/m)

0	200	400	600	800	1000

Frequency (MHz)

3.0x10^-6^

(**D**)

2.5x10^-6^

Attenuation coefficient (Np/m)

2.5x10^-6^

2.0x10^-6^

y=-7.01e^-14^x^3^+5.17e^-11^x^2^-6.46e^-9^x+2.05e^-7^

2

2.0x10^-6^

1.5x10^-6^

y=-7.58e^-14^x^3^+5.55e^-11^x^2^-6.90e^-9^x+2.25e-7 R^2^=99.71%

-12	2	-8	-6

1.5x10

-6 R =99.72%

y=-2.22e^-12^x^2^+7.90e^-9^x-2.34e^-6^

R^2^=99.37%

y=-4.63e	x +1.04e x-2.94e

R^2^=98.66%

1.0x10^-6^

5.0x10^-7^

0.0

-5.0x10^-7^

PartⅠ

Part Ⅱ

1.0x10^-6^

5.0x10^-7^

0.0

PartⅠ

Part Ⅱ

0	200	400	600	800	1000

Frequency (MHz)

0	200	400	600	800	1000

Frequency (MHz)

3.5x10^-6^	(**E**)

3.0x10^-6^

Attenuation coefficient (Np/m)

2.5x10^-6^

2.0x10 y=-6.91e^-14^x^3^+5.21e^-11^x^2^-7.25e^-9^x+2.52e-7 R^2^=99.69%

-6

1.5x10^-6^

1.0x10^-6^

y=-6.46e^-11^x^2^+6.12e^-9^x-1.97e-6 R^2^=99.58%

3.5x10^-6^

1#

3.0x10^-6^	2#

Attenuation coefficient (Np/m)

3#

2.5x10^-6^	4#

5#

2.0x10^-6^

1.5x10^-6^

1.0x10^-6^

(**F**)

① ②

5.0x10^-7^

0.0

-5.0x10^-7^

PartⅠ

Part Ⅱ

5.0x10^-7^

Ⅰ	Ⅱ

0.0

-5.0x10^-7^

0	200	400	600	800	1000

Frequency (MHz)

200	400	600	800	1000

Frequency (MHz)

**Fig. 8.** ~~Variations~~ Changes of attenuation coefficient with frequency.

**Fig. 9.** Schematic diagram of radar measurement array (1-transmitter, 2-receiver) and sources of reflectedi~~on~~ (3,4) and refractedi~~on~~ signals (5,6).

**Optimization**

**Fig. 10.** Simulation - calculation process flow.

Solve

Time-Domain Gprmax signal

**Matlab**

Two-way travel time and reflection coeff.

Define propagation control equations

Define parameter and setting

Physical model

GprMax Modeling

**Optimization**

**(**Ⅲ**)**

**(**Ⅱ**)**

**(**Ⅰ**)**

**Fig. 11.** Simulation model with GprMax modeling.

1000

h_1_

800

1000

20

Amplitude (mv/m)

10	800

0

h

h_1_

15	h

Amplitude (mv/m)

10	2

600

Amplitude (mv/m)

2	-10

600	5 B

400

Amplitude (mv/m)

-20

2.0x10-8 2.5x10-8 3.0x10-8 3.5x10^-8^

Time (s)

400

0	B

-5

-10

-15

200

0

-200

-400

-600

B

C

(**M1**)

200

0

-200

-400

-600

3.5x10-8	4.0x10-8	4.5x10-8	5.0x10^-8^

Time (s)

C

(**M2**)

0.0	2.0x10^-8^	4.0x10^-8^	6.0x10^-8^	8.0x10^-8^

Time (s)

0.0	2.0x10^-8^	4.0x10^-8^	6.0x10^-8^	8.0x10^-8^

Time (s)

1000

800

h_1_

10

Amplitude (mv/m)

h2

1000

800

h_1_

5.0

Amplitude (mv/m)

h_2_

600

Amplitude (mv/m)

400

200

5

B

0

-5	C

600

400

Amplitude (mv/m)

200

2.5

B

0.0

-2.5

C

-5.0

0

-200

-400

5x10-8 Time (s)

6x10^-8^

(**M3**)

0

-200

-400

-600

6.0x10^-8^	6.5x10^-8^	7.0x10^-8^	7.5x10^-8^

Time (s)

(**M4**)

0.0	2.0x10^-8^	4.0x10^-8^	6.0x10^-8^	8.0x10^-8^

Time (s)

0.0	2.0x10^-8^	4.0x10^-8^	6.0x10^-8^	8.0x10^-8^

Time (s)

1000

800

h_1_

2	h_2_

1000

800

h_1_

1.0

Amplitude (mv/m)

h_2_

600

400

Amplitude (mv/m)

200

1	B	600

0

Amplitude (mv/m)

Amplitude (mv/m)

400

-1

C	200

0.5

B

0.0

-0.5

C

-1.0

0

-200

-400

-600

-2

7x10^-8^	8x10^-8^	9x10^-8^

Time (s)

(**M5**)

0.0	2.0x10^-8^	4.0x10^-8^	6.0x10^-8^	8.0x10^-8^	1.0x10^-7^

Time (s)

0

-200

-400

-600

8x10^-8^	9x10^-8^	1x10^-7^

Time (s)

(**M6**)

0.0	2.0x10^-8^	4.0x10^-8^	6.0x10^-8^	8.0x10^-8^	1.0x10^-7^

Time (s)

1000

800

600

400

Amplitude (mv/m)

200

h_1_

0.50	h2

Amplitude (mv/m)

0.25

B

0.00

-0.25	C

-0.50

9.5x10^-8^	1.0x10^-7^	1.1x10^-7^	1.1x10^-7^	1.2x10^-7^

_0_	Time (s)

-200

-400

-600

(**M7**)

0.0	2.0x10^-8^	4.0x10^-8^	6.0x10^-8^	8.0x10^-8^	1.0x10^-7^	1.2x10^-7^

Time (s)

**Fig. 12.** Propagation law of UWB radar waves in coal.

h*_i_* is two-way travel time of radar wave between two objects [52]. The meaning of B and C is

given in Fig. 11

**Fig. 13.** Variations of echo signal of person with thickness of coal (L_1_).
