# Supplementary material for: Propagation of ground penetrating radar waves in Chinese coals
Source: PLoS One. 2020 May 21;15(5):e0233434. doi: 10.1371/journal.pone.0233434 (PMC7241710; doi:10.1371/journal.pone.0233434)
Supplement: Attachment — Submitted filename: Response to reviewers.docx [file pone.0233434.s005.docx]

**Reply to Reviewers’ Comments**

**Journal:** PLOS ONE

**Manuscript ID:** PONE-D-19-29325

Title: “Propagation of Ground Penetrating Radar Waves in Chinese Coals”

**Authors:** Duo Zhang, Rui Tang, Hu Wen, Shixing Fan

| **Editor** | |
| --- | --- |
| **Comments to the Author** | **Response** |
| Thank you for submitting your manuscript to PLOS ONE. After careful consideration, we feel that it has merit but does not fully meet PLOS ONE’s publication criteria as it currently stands. Therefore, we invite you to submit a revised version of the manuscript that addresses the points raised during the review process. We would appreciate receiving your revised manuscript by Feb 04 2020 11:59PM. Two independent reports have been collected for your submission. Both the reports are generally encouraging, but recommend major improvements. | We thank the anonymous reviewers for taking precious time to review our manuscript. We appreciate the reviewers giving us valuable comments that are helpful to improve our manuscript. Here we attempt to reply where and how we revised our manuscript in the light of reviewers’ comments. Revised contents of our manuscript and our replies are marked in red font. |

| **Reviewer #1** | |
| --- | --- |
| **Comments to the Author** | **Response** |
| The key contribution of this paper is that it reports the effects of EM wave frequencies on two fundamental parameters or properties: resistivity and dielectric permitivity for different rank coal samples. I am not quite sure one can claim the outcome/results of the lab testing of these parameter can be declared as “Law”. The claim of the Ultra-wideband radar wave is also arguable as the radar wave used is not necessarily ultra-wideband but the frequency was varied from 100 Mhz – 1000 MHz. Otherwise they would not be able to obtain the parameter variations with frequencies. Based on these measurements, it also derived the EM wave attenuation and speed variations with frequencies for different rank coal samples.  The speed is computed by using the simplified version of the wave speed formula (3). I suggest you may also compute with a more accurate formula such as (Zhou and Fullagar, 2001):  So that it may get different values to the simplified one. Surely the results may do not change much as the frequencies used are relatively high and the approximated version could provide sufficient accuracy.  One of the objectives of the paper is to use GPR for detection of mine trapped personnel after a coal mine disaster. With a frequency ranging from 100 MHz to 1000 MHz, I wonder what kind of penetration capability it will be although the paper claims, in Introduction, the GPR has “strong penetration”. The detection examples in Figures 11 & 12 are simplified too much with no practical use at all. At most, you only illustrate you can estimate the distances of reflection objects without knowing which direction they come from. Such ability is well illustrated in seismic and GPR textbooks. GPR is relatively matured technique. Is there any successful examples such as from controlled experiments in coal mine environment? I guess not. Even if you can receive the reflections from trapped personnel, you need to address the issue such as how to differentiate the human reflections from many other reflections from imhomogeinities of the strata, underground equipment and objects. If you cannot resolve this issue, your GPR measurements have no use to this particular applications.  Based on my above comments, I suggest the author concentrated on the property measurement report rather than try also to address the trapped human detection. Properly understanding the property of coal samples for different frequencies is a good contribution to the literature.  In addition, please improve your English expression of the paper to ensure communicate your finding to your reader effectively. This can be achieved by asking a native English speaker or English Editing services to help you before submit your paper. | Thanks for the reviewer’s comments. In the revised manuscript, we have modified the title as well as removed the word of “Law”. The controversial term UWB to GPR. As shown in Figure 1, we have developed a prototype radar device that can penetrate the coal seam to detect life information. During the research, we tested the dielectric constant of coal and analyzed the propagation of radar waves in the coal. At the initial stage of research, we have assumed some basic physical models. Experts from the China National Key R & D Project team tested our radar prototype at Jining No. 2 Coal Mine. The results show that the radar can penetrate 5m coal seam.    Fig. 1 Radar prototype for penetration detection    Fig. 2 Schematic of radar structure  1: battery; 2: Control module of power; 3: Data transmission module;  4: CPU; 5: Data acquisition module; 6: Receiving antenna;  7: Transmitting antenna; 8: Gyro; 9: Transmission line; 10: Computer  We use a combination of the delay band intersection algorithm and the gyroscope at the bottom of the radar to determine the direction of the target. The electrical parameters of strata and human beings are different. Also the directions of reflected waves are different. Therefore, it is possible to distinguish between reflected waves of people and strata.  Underground equipments and objects bring enormous interference waves during the detection process. We use the following method to screen effective waves. FIR low-pass filter is made use of eliminating direct waves and some background noises. The adaptive minimum mean square algorithm is used to eliminate periodic interference waves and narrowband waves in order to improve the signal-to-noise ratio.  The speed formula was simplified from the accurate formula suggested by the reviewer. We find that the difference between the simplified formula and theaccurate formula was very small. To the certain accuracy, the difference between the results of the two formulas can no longer be seen. For the rigor of the manuscript, we decided to accept the reviewer's opinion that the exact formula was chosen.  The article has been edited by academic editing with the native language. |

| **Reviewer #2** | |
| --- | --- |
| **Comments to the Author** | **Response** |
| General remarks: The paper presents data from laboratory testing of electrical properties of various coal samples taken from Chinese mines. Test were performed on a commercial Broadband Dielectric Spectrum Test System -Concept-80. It allows to determine the values of dielectric permittivity and specific resistivity of grinded coal samples subjected to the electromagnetic fields in wide frequency spectrum. On the base of laboratory data a set of approximating equations showing the change of electric properties in frequency domain ranging from 100-1000 MHz was obtained. These equations together with known from EM theory formulas for EM waves speed and attenuation were used for modeling and studying the process of radar waves propagation in a coal seam and reflection from the miners entrapped in collapsed tunnels. The modeling concept assumes that a radar transducer with dominating frequency MHz will be lowered through a vertical or inclined rescue borehole to the depth of coal seam with mining opening. It is assumed that in not collapsed part of tunnel only miner is present. Thus on the path of radar wave from transducer to a human we have only one geological interface reflecting the radar signals (coal-air) and one connected with human body. The human reflector can be located in various distances from the tunnel face. This factor was included in analyzed geometrical model. On the base of modeling results the authors of the paper conclude that a reliable human origin reflected radar signal can be measured when a distance between human and transducer do not exceed 5 m.  Although the concept and research data presented in the manuscript are very interesting and valuable scientifically it needs a thorough revision. There are a lot of deficiencies. Starting from the title, the next parts of manuscript (abstract, introduction and conclusions) in my opinion should be completely re written (my general remarks can be used as a new abstract in the paper). The rest of them need to be corrected. I suggest to remove figures 1 and 2 from the paper. They do not bring nothing and are detached from the content of the paper. Some other figures also need corrections. I recommend to send the manuscript back to authors for major revision. My detailed remarks are included in a word processor file, attached to the revision. I hope that they help the authors to correct the manuscript | 1. We thank the reviewer carefully checked on this part. The title has been modified to Thank you very much for your detailed suggestions and comments on the manuscript. In particular, the reviewers' general remarks can be directly used as a new abstract of the paper. We have carefully revised the manuscript based on the comments. For example, Figures 1 and 2 have been removed from the paper. We have checked and corrected the words, descriptions, spelling and grammar in the draft in detail. |
