# Supplementary material for: Propagation of ground penetrating radar waves in Chinese coals
Source: PLoS One. 2020 May 21;15(5):e0233434. doi: 10.1371/journal.pone.0233434 (PMC7241710; doi:10.1371/journal.pone.0233434)
Supplement: Attachment — Submitted filename: PONE-D-19-29325_R2_reviewer2.docx [file pone.0233434.s006.docx]

PLOS ONE

Propagation of Ground Penetrating Radar Waves in Chinese Coals

--Manuscript Draft--

| **Manuscript Number:** | PONE-D-19-29325R1 |
| --- | --- |
| **Article Type:** | Research Article |
| **Full Title:** | Propagation of Ground Penetrating Radar Waves in Chinese Coals |
| **Short Title:** | Propagation of Ground Penetrating Radar Waves in Chinese Coals |
| **Corresponding Author:** | Duo Zhang, Ph.D.  Xi'an University of Science and Technology Xi'an, Shaanxi CHINA |
| **Keywords:** | Coal; Dielectric characterization; UWB; Relative dielectric constant; Specific resistivity; Propagation law |
| **Abstract:** | The paper presents data from laboratory testing of electrical properties of various coal samples taken from Chinese mines. Test were performed on a commercial Broadband Dielectric Spectrum Test System -Concept-80. It allows to determine the values of dielectric permittivity and specific resistivity of grinded coal samples subjected to the electromagnetic fields in wide frequency spectrum. On the base of laboratory data a set of approximating equations showing the change of electric properties in frequency domain ranging from 100-1000 MHz was obtained. These equations together with known from EM theory formulas for EM waves speed and attenuation were used for modeling and studying the process of radar waves propagation in a coal seam and reflection from the miners entrapped in collapsed tunnels. The modeling concept assumes that a radar transducer with dominating frequency MHz will be lowered through a vertical or inclined rescue borehole to the depth of coal seam with mining opening. It is assumed that in not collapsed part of tunnel only miner is present. Thus on the path of radar wave from transducer to a human we have only one geological interface reflecting the radar signals (coal-air) and one connected with human body.  The human reflector can be located in various distances from the tunnel face. This factor was included in analyzed geometrical model. On the base of modeling results the authors of the paper conclude that a reliable human origin reflected radar signal can be measured when a distance between human and transducer do not exceed 5 m. |
| **Order of Authors:** | Duo Zhang, Ph.D. |
|  | Rui Tang |
|  | Hu Wen |
|  | Shixing Fan |
| **Response to Reviewers:** | We thank the anonymous reviewers for taking precious time to review our manuscript. We appreciate the reviewers giving us valuable comments that are helpful to improve our manuscript. Here we attempt to reply where and how we revised our manuscript in the light of reviewers’ comments. Revised contents of our manuscript and our replies are marked in red font.  Reviewer #1  Thanks for the reviewer’s comments. In the revised manuscript, we have modified the title as well as removed the word of “Law”. The controversial term UWB to GPR. As shown in Figure 1, we have developed a prototype radar device that can penetrate the coal seam to detect life information. During the research, we tested the dielectric constant of coal and analyzed the propagation of radar waves in the coal. At the initial stage of research, we have assumed some basic physical models. Experts from the China National Key R & D Project team tested our radar prototype at Jining No. 2 Coal Mine. The results show that the radar can penetrate 5m coal seam. We use a combination of the delay band intersection algorithm and the gyroscope at the bottom of the radar to determine the direction of the target. The electrical parameters of strata and human beings are different. Also the directions of reflected waves are different.  Therefore, it is possible to distinguish between reflected waves of people and strata. Underground equipments and objects bring enormous interference waves during the |

*Powered by Editorial Manager® and ProduXion Manager® from Aries Systems Corporation*

|  | detection process. We use the following method to screen effective waves. FIR low- pass filter is made use of eliminating direct waves and some background noises. The adaptive minimum mean square algorithm is used to eliminate periodic interference waves and narrowband waves in order to improve the signal-to-noise ratio.  The speed formula was simplified from the accurate formula suggested by the reviewer. We find that the difference between the simplified formula and theaccurate formula was very small. To the certain accuracy, the difference between the results of the two formulas can no longer be seen. For the rigor of the manuscript, we decided to accept the reviewer's opinion that the exact formula was chosen.  The article has been edited by academic editing with the native language.  Reviewer #2  We thank the reviewer carefully checked on this part. The title has been modified to Thank you very much for your detailed suggestions and comments on the manuscript. In particular, the reviewers' general remarks can be directly used as a new abstract of the paper. We have carefully revised the manuscript based on the comments. For example, Figures 1 and 2 have been removed from the paper. We have checked and corrected the words, descriptions, spelling and grammar in the draft in detail. |
| --- | --- |
| **Additional Information:** |  |
| **Question** | **Response** |
| **Financial Disclosure**  Enter a financial disclosure statement that describes the sources of funding for the work included in this submission. Review the [submission guidelines](http://journals.plos.org/plosone/s/submission-guidelines#loc-financial-disclosure-statement) for detailed requirements. View published research articles from [*PLOS ONE*](http://journals.plos.org/plosone/) for specific examples.  This statement is required for submission and **will appear in the published article** if the submission is accepted. Please make sure it is accurate. | Funding: The project was supported by the National Natural Science Foundation of China (grant numbers 51904234 and 51974240) and the Key R & D Program of Shaanxi Provincial (grant number 2017ZDCXL-GY-01-02-03). |

*Powered by Editorial Manager® and ProduXion Manager® from Aries Systems Corporation*

| **Unfunded studies**  Enter: *The author(s) received no specific funding for this work.*  **Funded studies**  Enter a statement with the following details:   - Initials of the authors who received each award - Grant numbers awarded to each author - The full name of each funder - URL of each funder website - Did the sponsors or funders play any role in the study design, data collection and analysis, decision to publish, or preparation of the manuscript? - **NO** - Include this sentence at the end of your statement: *The funders had no role in study design, data collection and analysis, decision to publish, or preparation of the manuscript.* - **YES** - Specify the role(s) played. |  |
| --- | --- |
| * typeset |  |
| **Competing Interests**  Use the instructions below to enter a competing interest statement for this submission. On behalf of all authors, disclose any [competing interests](http://journals.plos.org/plosone/s/competing-interests) that could be perceived to bias this work—acknowledging all financial support and any other relevant financial or non- financial competing interests.  This statement **will appear in the published article** if the submission is accepted. Please make sure it is accurate. View published research articles from [*PLOS ONE*](http://journals.plos.org/plosone/) for specific examples. | The authors have declared that no competing interests exist. |

*Powered by Editorial Manager® and ProduXion Manager® from Aries Systems Corporation*

| **NO authors have competing interests**  Enter: *The authors have declared that no competing interests exist.*  **Authors with competing interests**  Enter competing interest details beginning with this statement:  *I have read the journal's policy and the authors of this manuscript have the following competing interests: [insert competing interests here]* |  |
| --- | --- |
| * typeset |  |
| **Ethics Statement**  Enter an ethics statement for this submission. This statement is required if the study involved:   - Human participants - Human specimens or tissue - Vertebrate animals or cephalopods - Vertebrate embryos or tissues - Field research   Write "N/A" if the submission does not require an ethics statement.  General guidance is provided below. Consult the [submission guidelines](http://journals.plos.org/plosone/s/submission-guidelines#loc-guidelines-for-specific-study-types) for detailed instructions. **Make sure that all information entered here is included in the Methods section of the manuscript.** | N / A |

*Powered by Editorial Manager® and ProduXion Manager® from Aries Systems Corporation*

| **Format for specific study types**  **Human Subject Research (involving human participants and/or tissue)**   - Give the name of the institutional review board or ethics committee that approved the study - Include the approval number and/or a statement indicating approval of this research - Indicate the form of consent obtained (written/oral) or the reason that consent was not obtained (e.g. the data were analyzed anonymously)   **Animal Research (involving vertebrate animals, embryos or tissues)**   - Provide the name of the Institutional Animal Care and Use Committee (IACUC) or other relevant ethics board that reviewed the study protocol, and indicate whether they approved this research or granted a formal waiver of ethical approval - Include an approval number if one was obtained - If the study involved *non-human primates*, add *additional details* about animal welfare and steps taken to ameliorate suffering - If anesthesia, euthanasia, or any kind of animal sacrifice is part of the study, include briefly which substances and/or methods were applied   **Field Research**  Include the following details if this study involves the collection of plant, animal, or other materials from a natural setting:   - Field permit number - Name of the institution or relevant body that granted permission |  |
| --- | --- |
|  |  |
| **Data Availability**  Authors are required to make all data underlying the findings described fully available, without restriction, and from the time of publication. PLOS allows rare exceptions to address legal and ethical concerns. See the [PLOS Data Policy](http://journals.plos.org/plosone/s/data-availability) and [FAQ](http://journals.plos.org/plosone/s/data-availability#loc-faqs-for-data-policy) for detailed information. | Yes - all data are fully available without restriction |

*Powered by Editorial Manager® and ProduXion Manager® from Aries Systems Corporation*

| A Data Availability Statement describing where the data can be found is required at submission. Your answers to this question constitute the Data Availability Statement and **will be published in the article**, if accepted. |  |
| --- | --- |
| **Important:** Stating ‘data available on request from the author’ is not sufficient. If your data are only available upon request, select ‘No’ for the first question and explain your exceptional situation in the text box. |  |
| Do the authors confirm that all data underlying the findings described in their manuscript are fully available without restriction? |  |
| **Describe where the data may be found in full sentences. If you are copying our sample text, replace any instances of XXX with the appropriate details.**   - If the data are **held or will be held in a public repository**, include URLs, accession numbers or DOIs. If this information will only be available after acceptance, indicate this by ticking the box below. For example: *All XXX files are available from the XXX database (accession number(s) XXX, XXX.*). - If the data are all contained **within the manuscript and/or Supporting Information files**, enter the following: *All relevant data are within the manuscript and its Supporting Information files.* - If neither of these applies but you are able to provide **details of access elsewhere**, with or without limitations, please do so. For example:   *Data cannot be shared publicly because of [XXX]. Data are available from the XXX Institutional Data Access / Ethics Committee (contact via XXX) for researchers who meet the criteria for access to confidential data.*  *The data underlying the results presented in the study are available from (include the name of the third party* | All relevant data are within the manuscript and its Supporting Information files |

*Powered by Editorial Manager® and ProduXion Manager® from Aries Systems Corporation*

| *and contact information or URL).*   - This text is appropriate if the data are owned by a third party and authors do not have permission to share the data.   * typeset |  |
| --- | --- |
| Additional data availability information: | Tick here if the URLs/accession numbers/DOIs will be available only after acceptance of the manuscript for publication so that we can ensure their inclusion before publication. |

*Powered by Editorial Manager® and ProduXion Manager® from Aries Systems Corporation*

Manuscript	[Click here to access/download;Manuscript;Manuscript.doc](https://www.editorialmanager.com/pone/download.aspx?id=25814830&guid=a12779b3-3dbb-45c5-9b38-72da5d784c09&scheme=1)

1 Propagation of Ground Penetrating Radar Waves in Chinese
2 **Coals**
3 Duo Zhang ^a,^ ^b,^ ^c,^ ^*^, Rui Tang ^a,^ ^b^, Hu Wen ^a,^ ^b,^ ^c^, Shixing Fan ^a,^ ^b^
4 a School of Safety Science and Engineering, Xi’an University of Science and Technology, Xi'an
5 Shaanxi 710054, China
6 b Key Laboratory of Mine and Disaster Prevention and Control of Ministry of Education, Xi'an
7 University of Science and Technology, Xi'an Shaanxi 710054, China
8 c Postdoctoral Mobile station of Mining Engineering, Xi'an University of Science and Technology,
9 Xi’an Shaanxi 710054, China
10 ^*^Corresponding author: Duo Zhang. E-mail addresses: [zhangd@xust.edu.cn](mailto:zhangd@xust.edu.cn)

11

12 Abstract: The paper presents data from laboratory testing of electrical properties of
13 various coal samples taken from Chinese mines. Test were performed on a
14 commercial Broadband Dielectric Spectrum Test System -Concept-80. It allows to
15 determine the values of dielectric permittivity and specific resistivity of grinded coal
16 samples subjected to the electromagnetic fields in wide frequency spectrum. On the
17 base of laboratory data a set of approximating equations showing the change of
18 electric properties in frequency domain ranging from 100-1000 MHz was obtained.
19 These equations together with known from EM theory formulas for EM waves speed
20 and attenuation were used for modeling and studying the process of radar waves
21 propagation in a coal seam and reflection from the miners entrapped in collapsed
22 tunnels. The modeling concept assumes that a radar transducer with dominating
23 frequency MHz will be lowered through a vertical or inclined rescue borehole to the
24 depth of coal seam with mining opening. It is assumed that in not collapsed part of
25 tunnel only miner is present. Thus on the path of radar wave from transducer to a
26 human we have only one geological interface reflecting the radar signals (coal-air)
27 and one connected with human body. The human reflector can be located in various
28 distances from the tunnel face. This factor was included in analyzed geometrical
29 model. On the base of modeling results the authors of the paper conclude that a
30 reliable human origin reflected radar signal can be measured when a distance between
31 human and 500 MHz transducer do not exceed 5-8 m.
32 Keywords: coal, dielectric permittivity, specific resistivity, radar wave, propagation.
33 1. Introduction
34 In China, coal is still main energy source for the foreseeable future [1, 2]. In
35 addition, it also accounts for a large proportion of the world's primary energy
36 consumption [3-5]. Therefore, safety issues in coal mining processes have always
37 been the focus and hotspot of research. Coal mine emergency rescue is the last barrier
38 to safety and security.
39 In recent years, with the progress in science, technology, management, and
40 quality of workers, the safety of coal mines has obviously improved. The total number
41 of accidents and death tolls have dropped significantly. However, major accidents in
42 coal mines still occur from time to time. For example, in 2016, there were two
43 particularly significant coal mine accidents and eight major accidents [6]. Coal mine
44 accidents cause serious casualties, as along with economic losses and adverse social
45 impacts [7]. Obviously, the sooner we knew the location and condition of the people
46 in distress, the greater the possibility that they would be rescued. We should consider
47 the ground rescue method when we encountered the following two situations. First
48 was that the roadway could not be used due to coal or rock fall, high temperature,
49 water, etc. Second was that the lives of rescue workers are threatened uncontrollably.
50 As of now, there have been some successful drilling rescue cases. For example, a
51 water permeable accident occurred in the Kuixi Coal Mine in USA in July 2002. A
52 mine collapse accident occurred in San Jose copper mine in Chile in August 2010 [8].
53 A mine collapse accident occurred in Yurong gypsum mine in China in December
54 2015 [9]. Long-distance drilling might be deflected during construction under the
55 current technical conditions (as shown in Fig. 3). Under these circumstances, life
56 information detection technologies, such as video or audio, infrared, and gas, would
57 not be available because they cannot penetrate the coal seam [10]. Among numerous
58 non-contact life information detection technologies, GPR radar was considered to be
59 the most effective technology due to its high resolution, deep penetration, and low
60 power radiation, which is suitable for the life rescue systems [11, 12]. It has been used
61 in life detection and rescue [13]. Many works have been conducted on life detection
62 based on radar [14-18]. Therefore, we believed that GPR radar technology might be
63 the future development direction of life information detection through coal seams.
64 This study showed that the propagation of GPR radar waves in coal, and the related
65 influencing factors have very important scientific and practical significance.
66 Numerous scholars have conducted extensive research on the electromagnetic
67 properties of coal and the propagation of electromagnetic waves in coal. Fan and Jia
68 et al studied dielectric properties of coals in the low-terahertz frequency region [19].
69 Fan and Chang et al studied the dielectric properties of coal in the terahertz frequency
70 region of 100–500 GHz [20]. Qin and Wei researched the relative dielectric constant
71 sweep measurement based on a vector network analyzer [21]. Brach studied the real
72 part of the permittivity of a series of coals as a function of frequency (100
73 Hz–1.3×10^7^ Hz) and temperature (200–400 K), and the results showed that certain
74 parameters are related to the microstructure of the carbonaceous phase [22]. Marland
75 et al. tested the dielectric properties of coal using the microwave cavity method in the
76 laboratory, and qualitatively discussed the effects of temperature, frequency, coal rank,
77 minerals and water content on the coal dielectric properties [23]. Valentina studied the
78 influence of polyethylene terephthalate on the carbonization of bituminous coals and
79 on the modification of their electric and dielectric properties [24]. The relationship
80 between interior moisture distribution and coal dielectric properties has been
81 constructed by Wang. The characteristics of permittivity variation with moisture
82 content has also been studied [25]. Peng studied the dielectric characteristics of an
83 Indonesian low-rank coal based on determination of its permittivity from 21^o^C to
84 approximately 1000^o^C at 915 and 2450 MHz in argon [26]. Tao et al., analyzed the
85 effect of sulfur content on the dielectric properties of coal [27]. Xu studied the
86 influence of the crystallite structure on the dielectric properties of coal chars [28]. Liu
87 studied the effect of coal rank on the dielectric properties through structural
88 differences in various coal chars [29]. Emslie et al. put forward the model of a coal
89 seam waveguide, for the first time, when studying radio wave communication in coal
90 mines [30]. Hill derived the theoretical formula of electromagnetic wave propagation
91 under the layered model and studied the propagation of electromagnetic waves in
92 uneven coal seams [31, 32]. Greenfield used finite difference simulations to analyze
93 the attenuation characteristics of electromagnetic waves in discontinuous coal seams
94 [33]. Luo and Stolarczyk et al., analyzed the effect of electrical properties of coal
95 seams on the attenuation characteristics of electromagnetic waves [34-37]. Zhu et al.
96 reviewed the research progress on the measurement technique of coal dielectric
97 constants and analyzed the dielectric models of several coals [38]. In summary, it is
98 easy to see that there are relatively few investigated on the electrical parameters of
99 different ranks of coal in the frequency range of 100-1000 MHz.
100 In this paper, we report an experimental study on the dielectric properties of five
101 coal samples of different ranks, with frequencies ranging from 100 MHz to 1000 MHz.
102 The propagation approximating equations of radar waves in coal is constructed, and
103 the propagation process is simulated and solved. The effective distance range of radar
104 waves penetration in coal was given.
105 2. Experiment and methods
106 *2.1. Dielectric properties measurement*
107 In this study, five kinds of coal samples were used, according to the rank of coal,
108 namely, lignite (1#), in the Dananhu Coal Mine of Xinjiang; long flame coal (2#), in
109 the Tangjiahui Coal Mine of Inner Mongolia; gas coal (3#), in the Baodian Coal Mine
110 of Shandong; lean coal (4#), in the Sangshuping Coal Mine of Shaanxi; anthracite
111 (5#), in the Huangyanhui Coal Mine of Shanxi. The proximate analysis and elemental
112 analysis results are shown in Table 1. The fresh coal samples that were not subjected
113 to water injection, spraying, and other treatments were sealed and packed with
114 multilayer plastic and nylon bags, and then transported to the laboratory. Each
115 experiment was performed four times to ensure test reproducibility. The samples were
116 prepared as follows. A fresh coal sample was crushed, and pulverized coal whose
117 diameter was less than 0.074 mm was screened out. A certain amount of pulverized
118 coal was weighed into a tableting machine to prepare a circular coal sample piece
119 having a diameter of 1.28 cm and a thickness of 1.0 mm. The conductive silver
120 colloid was evenly coated on both sides of the coal sample, and could be tested after
121 the silver gel was dried. The preparation process of the sample is shown in Fig. 4.
122 The experimental equipment was a Concept-80 broadband dielectric spectrum
123 test system, which mainly included control software, an E4991A impedance analyzer
124 and test fixture, as shown in Fig. 5.
125 *2.2. The Propagation equations of Radar Waves in Coal*
126 *2.2.1. Dielectric properties of tested coal samples*
127 The relative dielectric constant values of the five tested coals are shown in Fig. 6,
128 and resistivity values are shown in Fig. 7.
129 It can be seen from Fig. 6 that the relative dielectric constants of five different
130 coals followed similar regularities. They decreased first and then increased with
131 increasing test frequency. The minimum values of dielectric constant appeared around
132 300 MHz, while the largest one were near 1000 MHz. At the same test frequency, lean
133 coal had the highest relative dielectric constant, followed by lignite, gas coal, long
134 flame coal, and finally anthracite, as shown in Fig. 6(F). The lignite contained a large
135 number of hydrophilic polar functional groups, which could adsorb a large amount of
136 intrinsic moisture. Polar functional groups and moisture had a high relative dielectric
137 constant. Therefore, the dielectric constant of lignite was higher. The order of relative
138 dielectric constants of the other four ranks of coal should be determined by the carbon
139 content in coal. The relative dielectric constant of lean coal was larger than that of
140 lignite because it was influenced by other factors, including porosity [22]. The
141 relative dielectric constants and frequencies were fitted and were found to satisfy the
142 cubic polynomial relationship. The results are shown in Eq. (1), and correlation
143 143
144 144

coefficients are shown in Table 2.

*ε* =*C*_3_ *f* ^3^ + *C*_2_ *f* ^2^ + *C*_1_ *f* + *C*_0_,( *f* ∈[100*MHz*,1000*MHz*])

(1)

145 Where ε is the relative dielectric permittivity, *f* is frequency, and *Ci* is a coefficient

146	(*i*=0, 1, 2, 3).

147 It could be seen from Fig. 7 that the resistivity of coal with a different degree of
148 metamorphism had an overall decreasing trend with an increase in test frequency.
149 There was a convex transformation point near 500 MHz, which could divide the trend
150 of resistivity with frequency into two stages. It was found, through fitting, that part Ⅰ
151 conformed to a cubic polynomial and part Ⅱ conformed to a quadratic polynomial.
152 The results were shown in Eq. (2) and Table 3. After 700 MHz, the resistivity of tested
153 coals tended to be flat and the differences between them are small. As the test
154 frequency increased, electrons a in bundled state of the coal structure group might be
155 converted into freely excited electrons. This caused a significant increase in free
156 radicals (ie, unpaired electrons) of coals, so the resistivity would decrease. Previous
157 studies have shown that anthracite was a resistive absorbing material [39], and the
158 resistivity of mineral impurities in it was higher than that of organic matter [40].
159 These factors might make the anthracite resistivity relatively large.
160 160

ρ ⎧*C*_13_ *f* ^3^ + *C*_12_ *f* ^2^ + *C*_11_ *f* + *C*_10_，( *f* ∈[100*MHz,*500*MHz*])

(2)

= ⎨	*C f* ^2^ + *C f* + *C* ，( *f* ∈(500*MHz,*1000*MHz*])

⎩	22	21	20

161 *2.2.2. The equation of radar wave propagation in coal*
162 To simplify the model, it was assumed that the coal body was a homogeneous
163 medium. Since radar wave~~s~~ are a type of electromagnetic waves, their propagation
164 velocity in coal could also be expressed by Eq. (3) [41]. From Eq. (3) and Fig. 8, the
165 propagation velocity of radar waves in different coals could be obtained, as shown in
166 166
167 167

Table 4.

*v =* ⎡ *ε*

(⎢

⎣ 2

+1)⎤−1 2	(3)

168 Where *v* is the propagation speed of radar waves in coal, in m/s. *ε* is the specific

1+ (1 / 2*π f ερ*)^2^

⎥⎦

169 relative dielectric permittivity of coal.
170 According to the literature [42], the attenuation coefficient of electromagnetic
171 wave propagation in coal could be characterized by Eq. (4). Combined with the
172 dielectric constant and electrical conductivity of coal, the variation of attenuation
173 coefficient with frequency could be obtained, as shown in Fig. 8

(⎢

⎥⎦

174 174

*a*=2*π f* ⎡ *εμ*

⎣ 2

1 + (1 2*π* *f ρε* )^2^ -1)⎤1 2	(4)

175 where *α* is the attenuation coefficient of radar wave in coal, *f* is the frequency, *ε* is
176 the relative dielectric permittivity of coal, and *ρ* is the specific resistivity of coal, μ is…..
177 It could be seen from Fig. 8 that the propagation attenuation coefficient of
178 electromagnetic waves in five types of coal followed a similar rule in relation to
179 frequency. It could be divided into two parts, according to the boundary line near 500
180 MHz (I and II). In part I, the attenuation coefficient was a cubic function of frequency. It
181 increased first and then decreased with increasing frequency. At the same frequency,
182 the attenuation coefficients of coals, from large to small, were lean coal, gas coal,
183 long flame coal, anthracite, and lignite. In part II, the attenuation coefficient was a
184 quadratic function of frequency. It increases with increasing frequency. In ①of part
185 Ⅱ, at the same frequency, the attenuation coefficient from large to small was
186 anthracite, long flame coal (gas coal, lean coal), and brown coal. Among them, the
187 attenuation coefficient of long flame coal, gas coal and lean coal had little difference.
188 In ②of part Ⅱ, at the same frequency, the attenuation coefficient from large to small
189 was anthracite (long flame coal, gas coal), lean coal, and brown coal. Among them,
190 the attenuation coefficient of anthracite, long flame coal, and gas coal were similar.
191 The attenuation coefficient and frequency were fitted, and results were shown in
192 192
193 193

Eq. (5) and Table 5.

⎧*C^'^*

*f* ^3^ + *C^'^ f* ^2^ + *C^'^ f* + *C^'^* ，( *f* ∈[100*MHz,*500*MHz*])

22	21	20

(5)

*a*= ⎨

13	12	11	10

⎩	*C'*

*f* ^2^ + *C^'^*

*f* + *C^'^* ，( *f* ∈(500*MHz,*1000*MHz*])

194 According to literature [43], reflection and refraction coefficients of radar waves
195 propagating in coal on boundary interfaces could be characterized by Eqs. (6) and (7).
196 196
197 197

Reflection coefficient： *Rij* =

Refraction coefficient： *T_ij_* =

(6)

(7)

*εi*	−	*ε j*

*εi* +	*ε j*

2 *ε_i_*

*ε_i_* + *ε _j_*

198 198

where

*Rij*

is the reflection coefficient of radar waves at the interface between

199 199

medium *i* and medium *j*.

*Tij*

is the refraction coefficient of radar waves at the

200 200

interface between medium *i* and medium *j*. *ε i*

is the relative dielectric constant of

201 201

medium *i*. *ε _j_*

is the relative dielectric constant of medium *j*.

202 From eqs. (6) and (7), respectively, the reflection and refraction coefficients on
203 various interfaces (as shown in Fig. 9) during radar wave propagation could be
204 obtained. The reflection and refraction coefficients of the interface were calculated by
205 taking the relative dielectric constant of coal at a frequency of 500 MHz as an
206 example. The results are shown in Table 6. It was not difficult to find that a negative
207 number appears in the reflection coefficient because the radar wave from the side with
208 a small relatively dielectric constant moves to the side with high relatively dielectric
209 constant, indicating that the reflected wave was opposite in phase to the incident
210 wave.
211 The propagation distance of radar waves in coal was determined by the
212 attenuation coefficient. It could be seen from Eq. (4) that the independent variables
213 determining variable *α* (attenuation coefficient) are ƒ (frequency), *ε* (relatively
214 214

dielectric constant), the *ρ* (resistivity), i.e.,

*α* =*F* ( *f* ,*ε* , *ρ* ) . As can be seen from

215 equation (1), *ε* (relatively dielectric constant) is a function of ƒ (frequency), i.e.,
216 *f* =*F* -1 (*ε* ). From Eq. (5), it is found that ƒ (frequency) is a function of *α* (attenuation
217 217

coefficient), i.e.,

*α* =*F* -1 ( *f* ) . Thus, the propagation attenuation law of radar waves in

218 218

coal is given by

⎧

⎪

⎪

*a*=2π*f* ⎡ ε

⎣ 2

⎢ (

-1)⎤1 2

1 + (1 2π*f* ρε)^2^

⎪	⎧*C'*

⎥⎦

*f* 3 + *C' f* 2 + *C'*

*f* + *C'*

, (*f* ∈[100*MHz,*500*MHz*])

⎪ *a*= ⎨

13	12	11	10

219 219

*C'	f* 2 + *C'	f* + *C'*	,	(*f* ∈(500*MHz,*1*.*0*GHz*])

(8)

⎨	⎩	22	21	20

⎪	ε=*C*3 *f* 3 + *C*2 *f* 2 + *C*1 *f* + *C*0 *,*( *f* ∈[100*MHz,*1*.*0*GHz*])

⎪

⎪	⎧*C*13 *f* 3 + *C*12 *f* 2 + *C*11 *f* + *C*10 ,	(*f* ∈[100*MHz,*500*MHz*])

⎪ρ= ⎨

*C f* 2 + *C*

*f* + *C*

,	(*f* ∈(500*MHz,*1*.*0*GHz*])

⎩	⎩	22

21	20

220 where *α* is the attenuation coefficient of radar wave propagation in coal. *f* is the

*C*

,

*ij*

221 221

frequency. *ε*	is	a	relative dielectric constant.	*ρ*	is the resistivity.

- *C_i_* ,

222 222

*Cij* ,(*i*, *j* ∈[0,1, 2,3])

are the coefficients of the corresponding function, respectively.

223 It was necessary to analyze the characteristics of waves to determine whether
224 there was anyone behind the coal when using radar for rescue. The characteristics of
225 waves were determined by laws of refraction and reflection, that is, Eq. (9), consisting
226 226
227 227

of Eqs. (6) and (7).

⎧

⎪*Rij* =

⎪

⎨

⎪*T* =

(9)

⎪ *ij*

⎩

228 228

*εi* −	*ε j*

*εi* +	*ε j*

2 *εi*

*εi* + *ε j*

where *ε i*

and *ε _j_*

are the relative dielectric constants of substance *i* and *j*,

229 229

respectively.

*Rij*

and

*Tij*

are the reflection and refraction coefficients, respectively.

230 According to the relationship between distance, speed and time, the distance
231 between the radar and person in distress can be calculated according to the following
232 formula.
233 233

⎧

⎪

⎡ *ε*

⎨*v=* ⎢ (

*L*=*v* ⋅ *t*

⎤−1 2 (10)

1 + (1 / 2*π f ερ*)^2^

+ 1)

⎥

⎩⎪	⎣ ^2^	⎦

234 where *v* is the propagation speed of radar waves in coal, in m/s.
235 The equations formed by the combination of Eqs. (8), (9), and (10) were the
236 control equations for the propagation of radar waves in coal.

⎧	*a*=2π*f* ⎡ ε

⎪	⎢

(

⎪	⎣ 2

-1)⎤1 2

⎪	⎧*C' f* 3 + *C' f* 2 + *C'*

1 + (1 2π*f* ρε)^2^

⎥⎦

*f* + *C'*

*,*(*f* ∈[100*MHz,*500*MHz*])

⎪*a*= ⎨

13	12	11	10

⎪	⎩	*C'*

*f* 2 + *C'*

*f* + *C'*

*,*(*f* ∈(500*MHz,*1*.*0*GHz*])

22	21	20

⎪	ε=*C*3 *f* 3 + *C*2 *f* 2 + *C*1 *f* + *C*0 *,*( *f* ∈[100*MHz,*1*.*0*GHz*])

⎪	⎧*C f* 3 + *C f* 2 + *C f* + *C ,*(*f* ∈[100*MHz,*500*MHz*])

*C	f*	+ *C	f* + *C	,*(*f* ∈ 500*MHz,*1*.*0*GHz* )(	]

237 237

⎪ρ= ⎨

⎪	⎩

⎨

⎪

⎪

⎪

⎪

⎪

⎪

13	12	11	10

2

22	21	20

ε*i*	−	ε *j*

ε*i*	+	ε *j*

2 ε*i*

ε*i* + ε *j*

*Rij* =

*Tij* =

(11)

⎪	*v=* ⎡ ε (

⎢

⎪

⎤−1 2

1 ⎥

1 + *(*1 */* 2π*f* ερ *)*^2^

+ )

238 238

⎪	⎣ 2

⎪⎩

3. Results and discussion

⎦

*L*=*v* ⋅ *t*

239 The equations (11) could be solved using GprMax code. GprMax is a simulation
240 software based on FDTD theory, which was developed by Dr. Antonis Giannopoulos
241 of the University of Edinburgh [44]. The GprMax has been widely used for simulation
242 and modelling of radar waves propagation in such GPR applications as underground
243 pipeline surveys, roadbed inspections, ancient building surveys, military surveys,
244 quality inspections, and hidden grave surveys [45-49]. The simulation calculation
245 process is shown in Fig. 10. The simulated physical model is shown in Fig. 11(Ⅰ).
246 As shown in Fig. 11(Ⅱ), the thickness of a coal body between a trapped person
247 and radar was L1, the distance between a person and coal wall was L2, the width of the
248 model was L3, the length of model was L4, and the size of a human body is 1.6 m ×0.4
249 m. The specific settings of different models are shown in Table 7. Then, the simulated
250 data was post-processed using Matlab, and the result is shown in Fig. 11(Ⅲ).
251 It was assumed that coal was a semi-infinite continuous space with homogeneous
252 isotropy. The dielectric constant value of a human was 50 [50]. The dielectric constant of air
253 was 1, the antenna stepping distance was 0.06 m, and the transmitting and receiving
254 antenna spacing was 0.065 m. The number of measuring lines was 115, the spatial
255 grid step was 0.005 m×0.005 m, the permeability of all media in the model was 1.0,
256 the boundary condition was a perfect matching layer (PML), the excitation function of
257 the radar was Ricker, and the center frequency was 500 MHz. The partial electrical
258 parameters of the model were set as shown in Table 8. Due to the large number of
259 graphs of simulation results, only lignite was taken as an example for analysis here.
260 The results are shown in Fig. 12.
261 From Fig. 12, we found that the amplitudes of return wave from the interface
262 between the coal pillar and roadway in seven models were 12.51, 5.48, 2.56, 1.23,
263 0.63, 0.30, and 0.15 mV/m, and the return amplitude of wave reflected from the human body is -20.63.

264	-10.24, -5.30, -2.72, -1.37, -0.69, and -0.35 mV/m. It was not difficult to find that the

265 intensity of amplitude gradually decreased with the increase of L1 (thickness of the
266 coal pillar). The larger the L1, the longer the electromagnetic wave travels in coal, and
267 the loss of electromagnetic wave energy also increased, so the intensity of the target
268 return wave amplitude was gradually smaller. By fitting, it was found that the
269 amplitude of the human body wave decreases with the increase of L1 as an
270 exponential function (as shown in Fig. 13). The effective propagation distance of
271 radar waves in coal was between 6 and 8 m for transducer with central frequency 500
272 MHz.
273 4. Conclusions
274 Dielectric properties of five different coals in from Chinese deposits were
275 studied using the alternating current impedance method and the Concept-80
276 broadband	dielectric	Spectrum	test	system	for	electromagnetic	waves	with
277 frequencies ranging from 100 MHz to 1000 MHz, for the first time, to the best of our
278 knowledge. The propagation process of radar waves in coal was analyzed.
279 The experimental results showed that the dielectric constants of five different
280 coals followed similar rules. The values of relative dielectric constant of coals
281 decreased first and then increased with frequency. At the same frequency, the order of
282 values of the relative permittivity of coal from high to low was lean coal, lignite, gas
283 coal, long flame coal, and anthracite. The values of specific resistivity of coal
284 decreased considerably with increasing frequency, but there was observed an upward
285 bump around frequency 500 MHz.
286 The key parameters of radar waves propagating in coal were analyzed. The
287 propagation velocity values of radar waves in different rank coals and the reflection
288 and refraction coefficients values at different types of interfaces were calculated. The
289 attenuation coefficient of radar waves in five kinds of coals followed a similar rule.
290 With 500 MHz as the dividing point, the relation between attenuation coefficient
291 of radar waves and frequency was divided into two parts. In part I, the frequency was
292 a cubic function of the attenuation coefficient. The attenuation coefficient initially
293 increases to a certain point and then decreases with increasing of frequency values. In
294 part II, the frequency was a quadratic function of the attenuation coefficient, and the
295 values of attenuation coefficient increased with frequency increase. A set of equations
296 for the propagation of radar waves in coal were established, based on the above
297 researches. By analyzing the governing equations, it was found that the effective
298 penetration distance of radar wave with dominant frequency 500MHz in coal ranges is
299 299
300 300

between 6 and 8 m.

301 Acknowledgements
302 Funding: The project was supported by the National Natural Science Foundation of
303 China (grant numbers 51904234 and 51974240) and the Key R & D Program of
304 Shaanxi Provincial (grant number 2017ZDCXL-GY-01-02-03).

References

1. Guo L, Zhai M, Wang Z, Zhang Y, Dong P. Comprehensive coal quality index for evaluation of coal agglomeration characteristics, Fuel 2018; 231(12): 379-386.
2. Song H, Liu J, Xue F, Cheng F. The application of ultra-fine fly ash in the seal coating for the wall of underground coal mine, Advanced Powder Technology 2016; 27: 1645-1650.
3. Diniz DA, Prasad P, Pagan RJ. Modern environmental improvement pathways for the coal power generation industry in Australia. Process Safety and Environmental Protection 2004; 82: 191-199.
4. Warmuzinski K. Harnessing methane emissions from coal mining. Process Safety and Environmental Protection 2008; 86: 315-320.
5. British Petroleum (BP), Statistical Review of World Energy, 2015. http://www.bp.

ucom/statistical review.

6. Wang D. Thermodynamic disaster in coal mine and its characteristics. Journal of China Coal Society 2018; 43(1): 137-142.
7. Wang K, Jiang S, Ma X, Wu Z, Shao H, Zhang W, et al. Information fusion of plume control and personnel escape during the emergency rescue of external-caused fire in a coal mine.

Process Safety and Environmental Protection 2016; 103: 46-59.

8. Ken B. Buried alive, but carbide drills at core of Chile miners' rescue. Metal Powder Report 2010; 65(7): 7-10.
9. Yang T, Du B. Construction Technology of Large Diameter rescue borehole in Pingyi gypsum mine disaster of Shandong, Exploration Engineering ( Rock & Soil Drilling and Tunneling) 2017, 44(5): 19-23. (In Chinese)
10. Wen H, Zhang D, Zheng X-Z. Research Progress and Trend of Life Detection Technology of Drilling and Rescue in Mine. Safety in Coal Mines 2017, 48(9): 85-88. (In Chinese)
11. Li Z, Li W, Lv H, Zhang Y, Jing X, Wang J. A novel method for respiration-like clutter cancellation in life detection by dual-frequency IR-UWB radar, IEEE Transactions on Microwave Theory and Techniques 2013; 61 (5): 2086-2092.
12. Hu W, Zhao Z, Wang Y, Zhang H, Lin F. Noncontact accurate measurement of cardiopulmonary activity using a compact quadrature Doppler radar sensor, I IEEE Transactions on Biomedical Engineering 2014; 61 (3): 725-735.
13. Chen K. Huang Y, Zhang J, Norman A. Microwave life-detection systems for searching human subjects under earthquake rubble or behind barrier, IEEE Transactions on Biomedical Engineering 2000; 47 (1): 105-114.
14. Jalali Bidgoli F, Moghadami S, Ardalan S. A compact portable microwave lifedetection device for finding survivors. IEEE Embedded Systems Letters 2016; 8(1): 10-13.
15. Liang X, Zhang H, Fang G, Ye S, Gulliver T. An improved algorithm for through wall target detection using ultra-wideband impulse radar. IEEE Access 2017; (99): 22101-22118.
16. Guan S, Rice JA, Li C, Gu C. Automated dc offset calibration strategy for structural health monitoring based on portable cw radar sensor. IEEE Transactions on Instrumentation and Measurement 2014; 63(12): 3111-3118.
17. Wang F, Horng TS, Peng K, Jau J, Li J, Chen C. Single-antenna Doppler radars using self and mutual injection locking for vital sign detection with random body movement cancellation. IEEE Transactinos on Microwave Theory and Techniques 2011; 59 (12): 3577-3587.
18. Liang X, Zhang H, Lyu T, Xu L, Cao C, Gulliver TA. Ultra-wide band impulse radar for life

detection using wavelet packet decomposition. Physical Communication 2018; 29:31-47.

19. Fan W, Jia C-Y, Hu W, Yang C, Liu L, Zhang X, et al. Dielectric properties of coals in the low-terahertz frequency region. Fuel 2015; 162: 294-304.
20. Fan W, Chang T, Liu L, Cui H. Dielectric properties of coal in the terahertz frequency region of 100–500 GHz. Fuel 2017; 188: 246-253.
21. Qin F, Wei G. Measurement with Sweeping Frequency for Complex Permittivity Based on VNA. Journal of Astronautic Metrology and Measurement 2011; 31(3): 27-30.
22. Brach I, Giuntini JC, Zanchetta JC. Real part of the permittivity of coals and their rank. Fuel 1994; 73(5): 738-741.
23. Marland S, Merchant A., Rowson N. Dielectric properties of coal. Fuel 2001; 80: 1839-1849.
24. Valentina VZ. Influence of polyethylene terephthalate on the carbonisation of bituminous coals and on the modification of their electric and dielectric properties. Fuel; 2006, 85:1652-1665.
25. Wang Q, Zhang X, Gu F. Investigation on interior moisture distribution inducing dielectric anisotropy of coals. Fuel processing technology 2005; 89:633-641.
26. Peng Z, Lin X, Li Z, Hwang J, Kim B, Zhang Y, et al. Dielectric characterization of Indonesian low-rank coal for microwave processing. Fuel processing technology 2017; 156: 171-177.
27. Tao X, Tang L, Xie M, He H, Xu N, Feng L, et al. Dielectric properties analysis of sulfur-containing models in coal and energy evaluation of their sulfur-containing bond dissociation in microwave field. Fuel 2016; 181: 1027-1033.
28. Xu L, Liu H, Jin Y, Fan B, Qiao X, Jing B. Structural order and dielectric properties of coal chars. Fuel 2014; 137: 164-171.
29. Liu H, Xu L, Jin Y, Fan B, Qiao X, Yang Y. Effect of coal rank on structure and dielectric

properties of chars. Fuel 2015; 153: 249-256.

30. Emslie AG, Lagace RL. Propagation of low and medium frequency radio waves in a coal seam.

Radio Science 1976; (11): 253-261.

31. Hill DA. Radio propagation in a coal seam and the inverse problem. Journal of Research of the National Bureau of Standards 1984; (89): 385-394.
32. Hill DA. Electromagnetic detection of long conductors in tunnels. IEEE Trans 1985; 33(4).
33. Greenfield RJ,Wu ST. Electromagnetic wave propagation in disrupted coal seams. Geophysics 1991; 56(10): 1571-1577.
34. Luo Y, Stolarczyk LG, Peng SD. Demonstration of the advanced radio imaging method (rim): ground truth results for three-dimensional imaging of coal seams. Sme Meeting 2004.
35. Stolarczyk LG, Fry RC. Radio imaging method (RIM) or diagnostic imaging of anomalous geologic structures in coal seam waveguides. Transactions of Society for Mining, Metallurgy, and Exploration, Inc. 1990; (288): 1806-1814.
36. Stolarczyk LG. Electromagnetic seam wave mapping of roof rock conditions across a longwall panel. 18th International Conference on Ground Control in Mining, Aug 3-5.1999.
37. Stolarczyk LG, Peng SS, Luo Y. Imaging ahead of Mining with Radio Imaging Method (RIM-IV) Instrumentation and Three-dimensional Tomography. Software Proceedings of 22nd International Conference on Ground Control in Mining 2003, Morgantown, WV, 136-143.
38. Zhu H, Xin M, Chang M, Liu D. Research progress on measuring technology of coal dielectric

constant. Coal Science Technology 2016; 44(9): 6-12．

39. Singclair DC, West AR. Impedance and modulus spectroscopy of semiconducting BaTiO3

showing positive temperature coefficient of resistance. Journal of Applied. Physics 1989; 66(8): 3850-3856.

40. Feng X, Chen J, Li N, Cui H, Liu J. Study on electromagnetic properties of anthracite and soft coal in microwave field. Study on electromagnetic properties of anthracite 2007; 38(5): 405-411. (In Chinses)
41. Zhou, B. and Fullagar, P.K., 2001, Delineation of sulphide ore-zones by borehole radar tomography at Hellyer Mine, Australia: Journal of Applied Geophysics, 47, No 3-4, pp.261-269.
42. Qi C. Ground penetrating radar signal processing in advanced detection in coal seam, Xi’an university of science and technology 2014. (In Chinese)
43. Liu X. Research the propagation rules of middle-high frequency electromagnetic wave in coal medium. China University of Mining and Technology 2012.
44. Giannopoulos A. Modelling ground penetrating radar by GprMax. Construction and Building Materials 2005; 19: 755-762.
45. Núñez-Nieto X, Solla M, Novob A, Lorenzo H. Three-dimensional ground-penetrating radar

methodologies for thecharacterization and volumetric reconstruction of underground tunneling. Construction and Building Materials 2014; 71: 551-560.

46. Adam DB, Jamie K, Pringle. Semblance analysis to assess GPR data from a five-year forensic study of simulated clandestine graves. Journal of Applied Geophysics 2016; 125: 17-44.
47. Lopera O, Milisavljevic N. Prediction of the effects of soil and target properties on the antipersonnel landmine detection performance of ground- penetrating radar: A Colombian case study. Journal of Applied Geophysics 2007; 63: 13-23.
48. Shangguan P, Imad L. AQ, Lahouar S. Pattern recognition algorithms for density estimation of asphalt pavement during compaction: a simulation study. Journal of Applied Geophysics 2014; 107: 8-15.
49. Jaw SW, Hashim M. Locational accuracy of underground utility mapping using ground penetrating radar. Tunnelling and Underground Space Technology Incorporating Trenchless Technology Research 2013; 35: 20-29.
50. Dogaru T, Nguyen L, Le C. Computer models of the human body signature for sensing through the wall radar applications. DTIC Document Adelphi MD 2007.
51. Lee JH, Wang W. Characterization of snow cover using ground penetrating radar for vehicle trafficability–Experiments and modeling. Journal of Terramechanics 2009; 46: 189-202.

Suggested referencing publications supplementing the authors findings:

Kotyrba A., Stańczyk K., Application of a GPR Technique for Monitoring of Simulated Underground Coal Gasification in a Large Scale Model. Near Surface Geophysics. Vol 11, nr 5. 2013: 505-515.

Kotyrba A., Stańczyk K., Sensing the Underground Coal Gasification by Ground Penetrating Radar**.** Acta Geophysica vol.65,  [Issue 6](https://link.springer.com/journal/11600/65/6/page/1).  1185–1196, 2017: 1185–1196

The GPR "in situ" test described in those publications are consistent with data presented by authors in regard to electrical properties of coal.

Table captions

**Table 1** Proximate and elemental analysis of coal.

**Table 2** Coefficients of fitting equation.

**Table 3** Coefficients of fitting equation.

**Table 4** Propagation speed of radar waves in coal (m/s).

**Table 5** Coefficients of fitting equation. **Table 6** Reflection and refraction coefficients. **Table 7** Size of model (m).

**Table 8** Dielectric characterization of Coal.

Coal

|  | Mad | Aad | Vad | FCad | C | H | O | N | S | (%) |
| --- | --- | --- | --- | --- | --- | --- | --- | --- | --- | --- |
| 1# | 8.80 | 36.62 | 24.68 | 29.90 | 38.94 | 4.01 | 22.00 | 1.15 | 0.27 | 10.11 |
| 2# | 5.77 | 11.93 | 32.32 | 49.98 | 64.86 | 5.63 | 19.20 | 1.73 | 0.58 | 13.34 |
| 3# | 2.33 | 15.92 | 35.45 | 46.30 | 63.92 | 4.85 | 17.40 | 2.37 | 0.51 | 6.16 |
| 4# | 0.53 | 24.49 | 12.95 | 62.03 | 62.51 | 3.76 | 17.00 | 1.61 | 3.41 | 9.19 |
| 5# | 1.20 | 8.84 | 7.85 | 82.11 | 81.82 | 3.79 | 4.90 | 0.85 | 1.49 | 16.20 |
| **Table 2** Coefficients of fitting equation. | | | | | | | | | | |

**Table 1** Proximate and elemental analysis of coal.

Proximate analysis (%)	Elemental analysis (%)	Porosity

| Coals | C3 | C2 | C1 | C0 | R^2^（%） |
| --- | --- | --- | --- | --- | --- |
| 1# | -9.24×10^-10^ | 1.84×10^-6^ | -9.14×10^-4^ | 3.62 | 98.98 |
| 2# | -5.82×10^-10^ | 1.17×10^-6^ | -5.43×10^-4^ | 3.07 | 98.77 |
| 3# | -6.34×10^-10^ | 1.28×10^-6^ | -5.96×10^-4^ | 3.35 | 99.21 |
| 4# | -7.90×10^-10^ | 1.52×10^-6^ | -6.58×10^-4^ | 3.78 | 99.40 |
| 5# | -6.42×10^-10^ | 1.34×10^-6^ | -6.86×10^-4^ | 3.05 | 98.49 |

**Table 3** Coefficients of fitting equation.

| Partion | Coefficient | 1# | 2# | 3# | 4# | 5# |
| --- | --- | --- | --- | --- | --- | --- |
|  | C13 | -8.00×10^-5^ | -5.45×10^-5^ | -4.96×10^-5^ | -4.08×10^-5^ | -7.48×10^-5^ |
|  | C12 | 0.10 | 0.07 | 0.06 | 0.05 | 0.10 |
| Ⅰ | C11 | -43.06 | -30.19 | -27.52 | -23.03 | -40.16 |
|  | C10 | 6.13×10^3^ | 4.45×10^3^ | 4.06×10^3^ | 3.43×10^3^ | 5.78×10^3^ |
|  | R2 | 98.40 % | 99.25 % | 99.25 % | 99.24 % | 98.95 % |
| C22 | | 1.28×10^-3^ | 9.50×10^-4^ | 8.74×10^-4^ | 7.99×10^-4^ | 1.12×10^-3^ |
| C21 | | -2.36 | -1.81 | -1.66 | -1.48 | -2.22 |
| C20 | | 1.21×10^3^ | 9.60×10^3^ | 8.80×10^2^ | 7.86×10^2^ | 1.10×10^3^ |

Ⅱ

R^2^	97.94 %	99.11 %	98.84 %	98.58 %	98.91 %

**Table 4** Propagation speed of radar waves in coal (m/s).

| Frequency (MHz) | 1# | 2# | 3# | 4# | 5# |
| --- | --- | --- | --- | --- | --- |
| 100 | 15.70×10^7^ | 17.23×10^7^ | 16.14×10^7^ | 15.53×10^7^ | 17.32×10^7^ |
| 200 | 16.01×10^7^ | 17.29×10^7^ | 16.64×10^7^ | 15.57×10^7^ | 17.43×10^7^ |
| 300 | 16.05×10^7^ | 17.32×10^7^ | 16.90×10^7^ | 15.59×10^7^ | 17.46×10^7^ |
| 400 | 16.03×10^7^ | 17.29×10^7^ | 16.64×10^7^ | 15.55×10^7^ | 17.46×10^7^ |
| 500 | 16.01×10^7^ | 17.23×10^7^ | 16.14×10^7^ | 15.53×10^7^ | 17.39×10^7^ |
| 600 | 15.94×10^7^ | 17.20×10^7^ | 16.64×10^7^ | 15.45×10^7^ | 17.37×10^7^ |
| 700 | 15.87×10^7^ | 17.12×10^7^ | 16.15×10^7^ | 15.38×10^7^ | 17.29×10^7^ |
| 800 | 15.81×10^7^ | 17.66×10^7^ | 16.42×10^7^ | 15.32×10^7^ | 17.26×10^7^ |
| 900 | 15.76×10^7^ | 17.14×10^7^ | 16.29×10^7^ | 15.30×10^7^ | 17.17×10^7^ |
| 1000 | 15.74×10^7^ | 16.84×10^7^ | 16.26×10^7^ | 15.26×10^7^ | 17.14×10^7^ |

**Table 5** Coefficients of fitting equation.

| Part | Coefficient | 1# | 2# | 3# | 4# | 5# |
| --- | --- | --- | --- | --- | --- | --- |
|  | C'13 | -8.31×10^-14^ | -6.70×10^-14^ | -7.01×10^-14^ | -7.58×10^-14^ | -6.91×10^-14^ |
|  | C'12 | 6.36×10^-11^ | 4.95×10^-11^ | 5.17×10^-11^ | 5.55×10^-11^ | 5.21×10^-11^ |
| Ⅰ | C'11 | -1.03×10^-8^ | -6.20×10^-9^ | -6.46×10^-9^ | -6.90×10^-9^ | -7.25×10^-9^ |
|  | C'10 | 4.71×10^--7^ | 1.97×10^-7^ | 2.05×10^-7^ | 2.25×10^-7^ | 2.52×10^-7^ |
|  | R2 | 99.36% | 99.72% | 99.72% | 99.71% | 99.69% |
| C'22 | | -3.31×10^-12^ | -1.31×10^-12^ | -2.22×10^-12^ | -4.63×10^-12^ | -6.46×10^-11^ |
| C'21 | | 8.44×10^-9^ | 6.78×10^-9^ | 7.90×10^-9^ | 1.04×10^-8^ | 6.12×10^-9^ |
| C'20 | | -2.61×10^-6^ | 2.04×10^-6^ | -2.34×10^-6^ | -2.94×10^-6^ | -1.97×10^-6^ |

Ⅱ

R^2^	98.88%	99.52%	99.37%	98.66%	99.58%

**Table 6** Reflection and refraction coefficients.

Coal	Type

(1)

air→coal

(2)

coal→air

(3)

air→person

(4)

person→air

(5)

coal→person

(6)

person→coal

*Rij*	-0.31	0.31	-0.75	0.75	-0.58	0.58

1#

*Tij*	0.698	1.31	0.25	1.75	0.42	1.58

*Rij*	-0.27	0.27	-0.75	0.75	-0.60	0.60

2#

*Tij*	0.28	1.27	0.25	1.75	0.40	1.60

*Rij*	-0.29	0.29	-0.75	0.75	-0.59	0.59

3#

*Tij*	0.71	1.29	0.25	1.75	0.41	1.59

*Rij*	-0.32	0.32	-0.75	0.75	-0.57	0.57

4#

*Tij*	0.68	1.32	0.25	1.75	0.43	1.57

*Rij*	-0.27	0.27	-0.75	0.75	-0.61	0.67

5#

*Tij*	0.737	1.27	0.25	1.75	0.39	1.67

**Table 7** Size of model (m).

| Model | M1 | M2 | M3 | M4 | M5 | M6 | M7 |
| --- | --- | --- | --- | --- | --- | --- | --- |
| L1 | 2 | 3 | 4 | 5 | 6 | 7 | 8 |
| L2 | 1.3 | 1.3 | 1.3 | 1.3 | 1.3 | 1.3 | 1.3 |
| L3 | 3.3 | 4.3 | 5.3 | 6.3 | 7.3 | 8.3 | 9.3 |
| L4 | 10 | 10 | 10 | 10 | 10 | 10 | 10 |
| **Table 8** Dielectric characterization of Coal**.** | | | | | | | |

| Coal | 1# | 2# | 3# | 4# | 5# |
| --- | --- | --- | --- | --- | --- |
| ε | 3.51 | 3.03 | 3.30 | 3.73 | 2.99 |
| ρ (Ω·m) | 328.95 | 290.07 | 267.78 | 242.41 | 318.53 |

Figure	[Click here to access/download;Figure;Figure captions.doc](https://www.editorialmanager.com/pone/download.aspx?id=25814832&guid=628a51ac-e92b-4a55-a72b-eba503a432a7&scheme=1)

Figure captions

**Fig. 1.** Schematic concept of and application of borehole GPR method for location of miner trapped in underground tunnel in coal mine.

**Fig. 2.** Flow chart of processing test of coal samples.

**Fig. 3.** Concept-80 Broadband Dielectric Spectrum Test System.

**Fig. 4.** Changes of the relative dielectric permittivity with frequency for various coals.

**Fig. 5.** Variations of ~~various~~ tested coals resistivity with frequency.

**Fig. 6.** Changes of attenuation coefficient with frequency.

**Fig. 7.** Schematic diagram of types of reflection and refraction.

**Fig. 8.** Simulation calculation flow.

**Fig. 9.** Geometrical model of rock strata applied in numerical simulation.

**Fig. 10.** A plot of signal amplitudes versus travel time.

**Fig. 11.** Relationship(or a plot) between amplitude of a radar signal reflected from a person and a coal seam thickness L1.

**Fig. 1.** Schematic concept of and application of borehole GPR method for location of miner trapped in underground tunnel in coal mine.

A is a three-dimensional diagram of rescue drilling during the tunnel collapseB is a schematic

diagram of the drilling offset.

**Slice of coal**

**Tableting**

**machine**

**Fig. 2.** Flow chart of processing test of coal samples.

**Fig. 3.** Concept-80 Broadband Dielectric Spectrum Test System.

3.64	3.14

(A)

1#

Fitting line

(B)

2#

Fitting line

3.62	3.12

3.60	3.10

Relative dielctric permittivity

Relative dielctric permittivity

3.58	3.08

3.56	3.06

3.54	3.04

3.52	3.02

3.50

3.48

200	400	600	800	1000

Frequency (MHz)

3.00

2.98

200	400	600	800	1000

Frequency (MHz)

3.42	3.88

(C)

3#

Fitting line

3.40

3.38

Relative dielctric permittivity

3.36

3.34

3.32

3.30

3.28

3.26

3.24

3.86

3.84

Relative dielctric permittivity

3.82

3.80

3.78

3.76

3.74

3.72

3.70

3.68

4#

Fitting line

(D)

200	400	600	800	1000

Frequency (MHz)

200	400	600	800	1000

Frequency (MHz)

3.08

3.06

3.04

Relative dielctric permittivity

3.02

3.00

2.98

3.9

3.8

(E)

5#

Fitting line

1#

2#

3#

4#

5#

(F)

3.7

Relative dielctric permittivity

3.6

3.5

3.4

3.3

3.2

2.96

2.94

200	400	600	800	1000

Frequency (MHz)

3.1

3.0

2.9

200	400	600	800	1000	1200

Frequency (MHz)

**Fig. 4.** Changes of the relative dielectric permittivity with frequency for various coals

**A**: lignite; B: long flame coal; C: gas coal; D: lean coal; E: anthracite; F: ~~various~~ collective plot for all tested coals.

1400

1200

1000

Resistivity (Ω*m)

800

600

1000

750

(**B**)

y=-5.45×10^-5^x^3^+0.07x^2^-30.19x+4.45×10^3^

R^2^=99.25%

y=9.50×10^-4^x^2^-1.81x+9.60×10^2^

R^2^=99.11%

Part **Ⅰ**

Part **Ⅱ**

Resistivity (Ω*m)

500

400

200

250

0

(**A**)

y=-8.00×10^-5^x^3^+0.10x^2^-43.06x+6.13×103 R^2^=98.40%

y=1.28×10^-3^x^2^-2.36x+1.21×103 R^2^=97.94%

Part **Ⅰ**

Part **Ⅱ**

200	400	600	800	1000

Frequency (108 Hz)

0

200	400	600	800	1000

Frequency (MHz)

900

800

700

600

Resistivity (Ω*m)

500

400

300

800

700

(**D**)

y=-4.08×10^-5^x^3^+0.05x^2^-23.03x+3.43×10^3^ R^2^=99.24%

y=7.99×10^-4^x^2^-1.48x+7.86×10^2^ R^2^=98.58%

Part **Ⅰ**

Part **Ⅱ**

600

500

Resistivity (Ω*m)

400

300

200

100

0

200	400	600	800	1000

(**C**)

y=-4.96×10^-5^x^3^+0.06x^2^-27.52x+4.06×10^3^ R^2^=99.25%

y=8.74×10^-4^x^2^-1.66x+8.80×10^2^

R^2^=98.84%

Part **Ⅰ**

Part **Ⅱ**

Frequency (10^8^ Hz)

200

100

200	400	600	800	1000

Frequency (MHz)

1200

1000

1200

1000

(**F**)

1#

2#

3#

4#

5#

Part **Ⅰ**

Part **Ⅱ**

800	800

(**E**)

y=-7.48×10^-5^x^3^+0.10x^2^-40.16x+5.78×10^3^ R^2^=98.95%

y=1.12×10^-3^x^2^-2.22x+1.10×10^3^

R^2^=98.91%

Part **Ⅰ**

Part **Ⅱ**

Resistivity (Ω*m)

Resistivity (Ω*m)

600	600

400	400

200	200

0

200	400	600	800	1000

Frequency (MHz)

0

200	400	600	800	1000

Frequency (MHz)

**Fig. 5.** Variations of various coals resistivity with frequency.

**A**: lignite; B: long flame coal; C: gas coal; D: lean coal; E: anthracite; F: ~~various coals~~ collective plot for all tested samples.

2.5x10^-6^

Attenuation coefficient (Np/m)

2.0x10^-6^

1.5x10^-6^

1.0x10^-6^

5.0x10^-7^

0.0

3.5x10^-6^

3.0x10^-6^

(**A**)

y=-8.31e^-14^x^3^+6.36e^-11^x^2^-1.03e^-8^x+4.71e^-7^

R^2^=99.36%

y=-3.31e^-12^x^2^+8.44e^-9^x-2.61e^-6^ R^2^=98.88%

Part**Ⅰ**

Part **Ⅱ**

(**B**)

y=-6.7e^-14^x^3^+4.95e^-11^x^2^-6.2e^-9^x+1.97e^-7^ R^2^=99.72%

y=-1.31e^-12^x^2^+6.78e^-9^x+2.04e^-6^ R^2^=99.52%

Part**Ⅰ**

Part **Ⅱ**

Attenuation coefficient (Np/m)

2.5x10^-6^

2.0x10^-6^

1.5x10^-6^

1.0x10^-6^

5.0x10^-7^

0.0

-5.0x10^-7^

| 0 | 200 | 400 | 600 | 800 | 1000 |  | 0 | 200 | 400 | 600 | 800 | 1000 |
| --- | --- | --- | --- | --- | --- | --- | --- | --- | --- | --- | --- | --- |
|  |  | Frequency | (MHz) |  |  |  |  |  | Frequency | (MHz) |  |  |

3.5x10^-6^

3.0x10^-6^

Attenuation coefficient (Np/m)

3.0x10^-6^

2.5x10^-6^

(**C**)

y=-7.01e^-14^x^3^+5.17e^-11^x^2^-6.46e^-9^x+2.05e^-7^ R^2^=99.72%

y=-2.22e^-12^x^2^+7.90e^-9^x-2.34e^-6^ R^2^=99.37%

Part**Ⅰ**

Part **Ⅱ**

(**D**)

y=-7.58e^-14^x^3^+5.55e^-11^x^2^-6.90e^-9^x+2.25e^-7^ R^2^=99.71%

y=-4.63e x +1.04e x-2.94e R^2^=98.66%

-12	2	-8	-6

Part**Ⅰ**

Part **Ⅱ**

Attenuation coefficient (Np/m)

2.5x10^-6^

2.0x10^-6^

1.5x10^-6^

1.0x10^-6^

5.0x10^-7^

0.0

-5.0x10^-7^

0

200	400

600	800	1000

2.0x10^-6^

1.5x10^-6^

1.0x10^-6^

5.0x10^-7^

0.0

0

200	400

600	800	1000

Frequency (MHz)	Frequency (MHz)

3.5x10^-6^

(**E**)

y=-6.91e^-14^x^3^+5.21e^-11^x^2^-7.25e^-9^x+2.52e^-7^ R^2^=99.69%

y=-6.46e^-11^x^2^+6.12e^-9^x-1.97e^-6^ R^2^=99.58%

Part**Ⅰ**

Part **Ⅱ**

3.0x10^-6^

Attenuation coefficient (Np/m)

2.5x10^-6^

2.0x10^-6^

1.5x10^-6^

1.0x10^-6^

3.5x10^-6^

3.0x10^-6^

1#

2#

3#

4#

5#

(**F**)

① ②

Ⅰ

Ⅱ

Attenuation coefficient (Np/m)

2.5x10^-6^

2.0x10^-6^

1.5x10^-6^

1.0x10^-6^

5.0x10^-7^

0.0

-5.0x10^-7^

| 0 | 200 | 400 | 600 | 800 | 1000 | 200 | 400 | 600 | 800 | 1000 |
| --- | --- | --- | --- | --- | --- | --- | --- | --- | --- | --- |
|  |  | Frequency | (MHz) |  |  |  |  | Frequency (MHz) |  |  |

5.0x10^-7^

0.0

-5.0x10^-7^

**Fig. 6.** Changes of attenuation coefficient with frequency. **A**: lignite; B: long flame coal; C: gas coal; D: lean coal; E: anthracite; F: collective plot for all tested samples.

**Fig. 7.** Schematic diagram of types of reflection and refraction.

Radar waves travel from one object to another. (1): air→coal; (2): coal→air; (3): air→person; (4): person→air; (5): coal→person; (6): person→coal.

Physical model

**Optimization**

GprMax Modeling

Time-Domain Gprmax signal

Define propagation control equations

Define parameter and setting

**Fig. 8.** Simulation calculation flow.

Solve

**Matlab**

Two-way travel time and reflection coeff.

**Optimization**

**(**Ⅲ**)**

**(**Ⅱ**)**

**(**Ⅰ**)**

**Fig. 9.** Geometrical model of rock strata applied in numerical simulation.

Ⅰ: Schematic of illustration of a disaster; Ⅱ: Schematic of simplified of physical model;Ⅲ: Display of physical model in GPR software.

1000

h

20

1

10

0

h_2_

-10

-20

2.0x10-8 2.5x10-8 3.0x10-8 3.5x10^-8^

Time (s)

B

C

(**M1**)

800

600

1000

800

h_1_

15	h

10

2

5

B

0

B

-5

-10

-15

3.5x10-8 4.0x10-8 4.5x10-8 5.0x10^-8^

Time (s)

C

(**M2**)

Amplitude (mv/m)

600

400	400

Amplitude (mv/m)

Amplitude (mv/m)

Amplitude (mv/m)

200	200

0	0

-200

-400

-600

1000

800

600

Amplitude (mv/m)

400

200

0

-200

-400

0.0

0.0

h_1_

10

h2

5

B

0

-5

5x10-8 Time (s)

C

6x10^-8^

(**M3**)

2.0x10^-8^	4.0x10^-8^	6.0x10^-8^	8.0x10^-8^

Time (s)

Amplitude (mv/m)

2.0x10^-8^	4.0x10^-8^	6.0x10^-8^	8.0x10^-8^

Time (s)

-200

-400

-600

1000

800

600

Amplitude (mv/m)

400

200

0

-200

-400

-600

0.0

0.0

h_1_

5.0

h_2_

2.5

B

0.0

-2.5

C

-5.0

6.0x10^-8^	6.5x10^-8^	7.0x10^-8^	7.5x10^-8^

Time (s)

(**M4**)

2.0x10^-8^	4.0x10^-8^	6.0x10^-8^	8.0x10^-8^

Time (s)

Amplitude (mv/m)

2.0x10^-8^	4.0x10^-8^	6.0x10^-8^	8.0x10^-8^

Time (s)

Amplitude (mv/m)

Amplitude (mv/m)

1000

800

600

400

Amplitude (mv/m)

200

0

-200

-400

-600

0.0	2.0x10^-8^	4.0x10^-8^	6.0x10^-8^	8.0x10^-8^	1.0x10^-7^

h_1_

2

h_2_

1

B

0

-1

C

-2

7x10^-8^

8x10^-8^

Time (s)

9x10^-8^

(**M5**)

Time (s)

1000

800

600

Amplitude (mv/m)

400

200

0

-200

-400

-600

0.0	2.0x10^-8^	4.0x10^-8^	6.0x10^-8^	8.0x10^-8^	1.0x10^-7^

h_1_

1.0

h_2_

0.5

B

0.0

-0.5

C

-1.0

8x10^-8^

9x10^-8^

1x10^-7^

Time (s)

(**M6**)

Time (s)

1000

h_1_

0.50

h_2_

0.25

B

0.00

-0.25

C

-0.50

9.5x10^-8^ 1.0x10^-7^ 1.1x10^-7^ 1.1x10^-7^ 1.2x10^-7^

Time (s)

(**M7**)

800

Amplitude (mv/m)

600

400

Amplitude (mv/m)

200

0

-200

-400

-600

0.0	2.0x10^-8^ 4.0x10^-8^ 6.0x10^-8^ 8.0x10^-8^ 1.0x10^-7^ 1.2x10^-7^

Time (s)

**Fig. 10.** A plot of signal amplitudes versus travel time.

Mx [in m] are the thickness of the coal body between the radar and the trapped person.

M1: 2 m; M2: 3 m; M3: 4 m; M4: 5 m; M5: 6 m; M6: 7 m; M7: 8 m.

h*i* is two-way travel time of radar wave between two objects [52]. The meaning of B and C is

given in Fig. 9

**Fig. 11.** Relationship ~~(or a plot)~~ between amplitude of a radar signal reflected from a

person and a coal seam thickness L1.

Supporting Information

[Click here to access/download](https://www.editorialmanager.com/pone/download.aspx?id=25789100&guid=2a82d716-a214-454b-83c2-3797e4e55e11&scheme=1)

[**Supporting Information** Certificate of English Editing.pdf](https://www.editorialmanager.com/pone/download.aspx?id=25789100&guid=2a82d716-a214-454b-83c2-3797e4e55e11&scheme=1)

Revised Manuscript with Track Changes

1 Propagation of Ground Penetrating Radar Waves in Chinese
2 **Coals**
3 Duo Zhang ^a,^ ^b,^ ^c,^ ^*^, Rui Tang ^a,^ ^b^, Hu Wen ^a,^ ^b,^ ^c^, Shixing Fan ^a,^ ^b^
4 a School of Safety Science and Engineering, Xi’an University of Science and Technology, Xi'an
5 Shaanxi 710054, China
6 b Key Laboratory of Mine and Disaster Prevention and Control of Ministry of Education, Xi'an
7 University of Science and Technology, Xi'an Shaanxi 710054, China
8 c Postdoctoral Mobile station of Mining Engineering, Xi'an University of Science and Technology,
9 Xi’an Shaanxi 710054, China
10 ^*^Corresponding author: Duo Zhang. E-mail addresses: [zhangd@xust.edu.cn](mailto:zhangd@xust.edu.cn)

11

12 Abstract: The paper presents data from laboratory testing of electrical properties of
13 various coal samples taken from Chinese mines. Test were performed on a
14 commercial Broadband Dielectric Spectrum Test System -Concept-80. It allows to
15 determine the values of dielectric permittivity and specific resistivity of grinded coal
16 samples subjected to the electromagnetic fields in wide frequency spectrum. On the
17 base of laboratory data a set of approximating equations showing the change of
18 electric properties in frequency domain ranging from 100-1000 MHz was obtained.
19 These equations together with known from EM theory formulas for EM waves speed
20 and attenuation were used for modeling and studying the process of radar waves
21 propagation in a coal seam and reflection from the miners entrapped in collapsed
22 tunnels. The modeling concept assumes that a radar transducer with dominating
23 frequency MHz will be lowered through a vertical or inclined rescue borehole to the
24 depth of coal seam with mining opening. It is assumed that in not collapsed part of
25 tunnel only miner is present. Thus on the path of radar wave from transducer to a
26 human we have only one geological interface reflecting the radar signals (coal-air)
27 and one connected with human body. The human reflector can be located in various
28 distances from the tunnel face. This factor was included in analyzed geometrical
29 model. On the base of modeling results the authors of the paper conclude that a
30 reliable human origin reflected radar signal can be measured when a distance between
31 human and transducer do not exceed 5 m.
32 Keywords: coal, dielectric permittivity, specific resistivity, radar wave, propagtion.
33 1. Introduction
34 In China, coal is still main energy source for the foreseeable future [1, 2]. In
35 addition, it also accounts for a large proportion of the world's primary energy
36 consumption [3-5]. Therefore, safety issues in coal mining processes have always
37 been the focus and hotspot of research. Coal mine emergency rescue is the last barrier
38 to safety and security.
39 In recent years, with the progress in science, technology, management, and
40 quality of workers, the safety of coal mines has obviously improved. The total number
41 of accidents and death tolls have dropped significantly. However, major accidents in
42 coal mines still occur from time to time. For example, in 2016, there were two
43 particularly significant coal mine accidents and eight major accidents [6]. Coal mine
44 accidents cause serious casualties, as along with economic losses and adverse social
45 impacts [7]. Obviously, the sooner we knew the location and condition of the people
46 in distress, the greater the possibility that they would be rescued. We should consider
47 the ground rescue method when we encountered the following two situations. First
48 was that the roadway could not be used due to coal or rock fall, high temperature,
49 water, etc. Second was that the lives of rescue workers are threatened uncontrollably.
50 As of now, there have been some successful drilling rescue cases. For example, a
51 water permeable accident occurred in the Kuixi Coal Mine in USA in July 2002. A
52 mine collapse accident occurred in San Jose copper mine in Chile in August 2010 [8].
53 A mine collapse accident occurred in Yurong gypsum mine in China in December
54 2015 [9]. Long-distance drilling might be deflected during construction under the
55 current technical conditions (as shown in Fig. 3). Under these circumstances, life
56 information detection technologies, such as video or audio, infrared, and gas, would
57 not be available because they cannot penetrate the coal seam [10]. Among numerous
58 non-contact life information detection technologies, GPR radar was considered to be
59 the most effective technology due to its high resolution, deep penetration, and low
60 power radiation, which is suitable for the life rescue systems [11, 12]. It has been used
61 in life detection and rescue [13]. Many works have been conducted on life detection
62 based on radar [14-18]. Therefore, we believed that GPR radar technology might be
63 the future development direction of life information detection through coal seams.
64 This study showed that the propagation of GPR radar waves in coal, and the related
65 influencing factors have very important scientific and practical significance.
66 Numerous scholars have conducted extensive research on the electromagnetic
67 properties of coal and the propagation of electromagnetic waves in coal. Fan and Jia
68 et al studied dielectric properties of coals in the low-terahertz frequency region [19].
69 Fan and Chang et al studied the dielectric properties of coal in the terahertz frequency
70 region of 100–500 GHz [20]. Qin and Wei researched the relative dielectric constant
71 sweep measurement based on a vector network analyzer [21]. Brach studied the real
72 part of the permittivity of a series of coals as a function of frequency (100
73 Hz–1.3×10^7^ Hz) and temperature (200–400 K), and the results showed that certain
74 parameters are related to the microstructure of the carbonaceous phase [22]. Marland
75 et al. tested the dielectric properties of coal using the microwave cavity method in the
76 laboratory, and qualitatively discussed the effects of temperature, frequency, coal rank,
77 minerals and water content on the coal dielectric properties [23]. Valentina studied the
78 influence of polyethylene terephthalate on the carbonization of bituminous coals and
79 on the modification of their electric and dielectric properties [24]. The relationship
80 between interior moisture distribution and coal dielectric properties has been
81 constructed by Wang. The characteristics of permittivity variation with moisture
82 content has also been studied [25]. Peng studied the dielectric characteristics of an
83 Indonesian low-rank coal based on determination of its permittivity from 21 ^o^C to
84 approximately 1000 ^o^C at 915 and 2450 MHz in argon [26]. Tao et al., analyzed the
85 effect of sulfur content on the dielectric properties of coal [27]. Xu studied the
86 influence of the crystallite structure on the dielectric properties of coal chars [28]. Liu
87 studied the effect of coal rank on the dielectric properties through structural
88 differences in various coal chars [29]. Emslie et al. put forward the model of a coal
89 seam waveguide, for the first time, when studying radio wave communication in coal
90 mines [30]. Hill derived the theoretical formula of electromagnetic wave propagation
91 under the layered model and studied the propagation of electromagnetic waves in
92 uneven coal seams [31, 32]. Greenfield used finite difference simulations to analyze
93 the attenuation characteristics of electromagnetic waves in discontinuous coal seams
94 [33]. Luo and Stolarczyk et al., analyzed the effect of electrical properties of coal
95 seams on the attenuation characteristics of electromagnetic waves [34-37]. Zhu et al.
96 reviewed the research progress on the measurement technique of coal	dielectric
97 constants and analyzed the dielectric models of several coals [38]. In summary, it is
98 easy to see that there are relatively few investigated on the electrical parameters of
99 different ranks of coal in the frequency range of 100-1000 MHz.
100 In this paper, we report an experimental study on the dielectric properties of five
101 coal samples of different ranks, with frequencies ranging from 100 MHz to 1000 MHz.
102 The propagation approximating equations of radar waves in coal is constructed, and
103 the propagation process is simulated and solved. The effective distance range of radar
104 waves penetration in coal was given.
105 2. Experiment and methods
106 *2.1. Dielectric properties measurement*
107 In this study, five kinds of coal samples were used, according to the rank of coal,
108 namely, lignite (1#), in the Dananhu Coal Mine of Xinjiang; long flame coal (2#), in
109 the Tangjiahui Coal Mine of Inner Mongolia; gas coal (3#), in the Baodian Coal Mine
110 of Shandong; lean coal (4#), in the Sangshuping Coal Mine of Shaanxi; anthracite
111 (5#), in the Huangyanhui Coal Mine of Shanxi. The proximate analysis and elemental
112 analysis results are shown in Table 1. The fresh coal samples that were not subjected
113 to water injection, spraying, and other treatments were sealed and packed with
114 multilayer plastic and nylon bags, and then transported to the laboratory. Each
115 experiment was performed four times to ensure test reproducibility. The samples were
116 prepared as follows. A fresh coal sample was crushed, and pulverized coal whose
117 diameter was less than 0.074 mm was screened out. A certain amount of pulverized
118 coal was weighed into a tableting machine to prepare a circular coal sample piece
119 having a diameter of 1.28 cm and a thickness of 1.0 mm. The conductive silver
120 colloid was evenly coated on both sides of the coal sample, and could be tested after
121 the silver gel was dried. The preparation process of the sample is shown in Fig. 4.
122 The experimental equipment was a Concept-80 broadband dielectric spectrum
123 test system, which mainly included control software, an E4991A impedance analyzer
124 and test fixture, as shown in Fig. 5.
125 *2.2. The Propagation equations of Radar Waves in Coal*
126 *2.2.1. Dielectric properties of tested coal samples*
127 The relative dielectric constant values of the five tested coals are shown in Fig. 6,
128 and resistivity values are shown in Fig. 7.
129 It can be seen from Fig. 6 that the relative dielectric constants of five different
130 coals followed similar regularities. They decreased first and then increased with
131 increasing test frequency. The minimum values of dielectric constant appeared around
132 300 MHz, while the largest one were near 1000 MHz. At the same test frequency, lean
133 coal had the highest relative dielectric constant, followed by lignite, gas coal, long
134 flame coal, and finally anthracite, as shown in Fig. 6(F). The lignite contained a large
135 number of hydrophilic polar functional groups, which could adsorb a large amount of
136 intrinsic moisture. Polar functional groups and moisture had a high relative dielectric
137 constant. Therefore, the dielectric constant of lignite was higher. The order of relative
138 dielectric constants of the other four ranks of coal should be determined by the carbon
139 content in coal. The relative dielectric constant of lean coal was larger than that of
140 lignite because it was influenced by other factors, including porosity [22]. The
141 relative dielectric constants and frequencies were fitted and were found to satisfy the
142 cubic polynomial relationship. The results are shown in Eq. (1), and correlation
143 143
144 144

coefficients are shown in Table 2.

*ε* =*C*_3_ *f* ^3^ + *C*_2_ *f* ^2^ + *C*_1_ *f* + *C*_0_,( *f* ∈[100*MHz*,1000*MHz*])

(1)

145 Where ε is the relative dielectric permittivity, *f* is frequency, and *Ci* is a coefficient

146	(*i*=0, 1, 2, 3).

147 It could be seen from Fig. 7 that the resistivity of coal with a different degree of
148 metamorphism had an overall decreasing trend with an increase in test frequency.
149 There was a convex transformation point near 500 MHz, which could divide the trend
150 of resistivity with frequency into two stages. It was found, through fitting, that part Ⅰ
151 conformed to a cubic polynomial and part Ⅱ conformed to a quadratic polynomial.
152 The results were shown in Eq. (2) and Table 3. After 700 MHz, the resistivity of tested
153 coals tended to be flat and the differences between them are small. As the test
154 frequency increased, electrons a in bundled state of the coal structure group might be
155 converted into freely excited electrons. This caused a significant increase in free
156 radicals (ie, unpaired electrons) of coals, so the resistivity would decrease. Previous
157 studies have shown that anthracite was a resistive absorbing material [39], and the
158 resistivity of mineral impurities in it was higher than that of organic matter [40].
159 These factors might make the anthracite resistivity relatively large.
160 160

ρ ⎧*C*_13_ *f* ^3^ + *C*_12_ *f* ^2^ + *C*_11_ *f* + *C*_10_，( *f* ∈[100*MHz,*500*MHz*])

(2)

= ⎨	*C f* ^2^ + *C f* + *C* ，( *f* ∈(500*MHz,*1000*MHz*])

⎩	22	21	20

161 *2.2.2. The equation of radar wave propagation in coal*
162 To simplify the model, it was assumed that the coal body was a homogeneous
163 medium. Since radar waves area type of electromagnetic wave, their propagation
164 velocity in coal could also be expressed by Eq. (3) [41]. From Eq. (3) and Fig. 8, the
165 propagation velocity of radar waves in different coals could be obtained, as shown in
166 166
167 167

Table 4.

*v =* ⎡ *ε*

(⎢

⎣ 2

+1)⎤−1 2	(3)

168 Where *v* is the propagation speed of radar waves in coal, in m/s. *ε* is the specific

1+ (1 / 2*π f ερ*)^2^

⎥⎦

169 relative dielectric permittivity of coal.
170 According to the literature [42], the attenuation coefficient of electromagnetic
171 wave propagation in coal could be characterized by Eq. (4). Combined with the
172 dielectric constant and electrical conductivity of coal, the variation of attenuation
173 coefficient with frequency could be obtained, as shown in Fig. 8
174 174

*a*=2*π f* ⎢	(

-1)⎥	(4)

⎡*εμ*

1 + (1 2*π* *f ρε* )^2^

⎣ 2

⎤1 2

⎦

175 where *α* is the attenuation coefficient of radar wave in coal, *f* is the frequency, *ε* is
176 the relative dielectric permittivity of coal, and *ρ* is the specific resistivity of coal.
177 It could be seen from Fig. 8 that the propagation attenuation coefficient of
178 electromagnetic waves in five types of coal followed a similar rule in relation to
179 frequency. It could be divided into two parts, according to the boundary line near 500
180 MHz. In part I, the attenuation coefficient was a cubic function of frequency. It
181 increased first and then decreased with increasing frequency. At the same frequency,
182 the attenuation coefficients of coals, from large to small, were lean coal, gas coal,
183 long flame coal, anthracite, and lignite. In part II, the attenuation coefficient was a
184 quadratic function of frequency. It increases with increasing frequency. In ①of part
185 Ⅱ, at the same frequency, the attenuation coefficient from large to small was
186 anthracite, long flame coal (gas coal, lean coal), and brown coal. Among them, the
187 attenuation coefficient of long flame coal, gas coal and lean coal had little difference.
188 In ②of part Ⅱ, at the same frequency, the attenuation coefficient from large to small
189 was anthracite (long flame coal, gas coal), lean coal, and brown coal. Among them,
190 the attenuation coefficient of anthracite, long flame coal, and gas coal were similar.
191 The attenuation coefficient and frequency were fitted, and results were shown in
192 192
193 193

Eq. (5) and Table 5.

⎧*C^'^*

*f* ^3^ + *C^'^ f* ^2^ + *C^'^ f* + *C^'^* ，( *f* ∈[100*MHz,*500*MHz*])

22	21	20

(5)

*a*= ⎨

13	12	11	10

⎩	*C'*

*f* ^2^ + *C^'^*

*f* + *C^'^* ，( *f* ∈(500*MHz,*1000*MHz*])

194 According to literature [43], reflection and refraction coefficients of radar waves
195 propagating in coal could be characterized by Eqs. (6) and (7).
196 196
197 197

Reflection coefficient： *Rij* =

Refraction coefficient： *T_ij_* =

(6)

(7)

*εi*	−	*ε j*

*εi*	+	*ε j*

2 *ε_i_*

*ε_i_* + *ε _j_*

198 198

where

*Rij*

is the reflection coefficient of radar waves at the interface between

199 199

medium *i* and medium *j*.

*Tij*

is the refraction coefficient of radar waves at the

200 200

interface between medium *i* and medium *j*. *ε i*

is the relative dielectric constant of

201 201

medium *i*. *ε _j_*

is the relative dielectric constant of medium *j*.

202 From eqs. (6) and (7), respectively, the reflection and refraction coefficients on
203 various interfaces (as shown in Fig. 9) during radar wave propagation could be
204 obtained. The reflection and refraction coefficients of the interface were calculated by
205 taking the relative dielectric constant of coal at a frequency of 500 MHz as an
206 example. The results are shown in Table 6. It was not difficult to find that a negative
207 number appears in the reflection coefficient because the radar wave from the side with
208 a small relatively dielectric constant moves to the side with high relatively dielectric
209 constant, indicating that the reflected wave was opposite in phase to the incident
210 wave.
211 The propagation distance of radar waves	in	coal	was	determined by	the
212 attenuation coefficient. It could be seen from Eq. (4) that the independent variables
213 determining variable *α* (attenuation coefficient) are ƒ (frequency), *ε* (relatively
214 214

dielectric constant), the *ρ* (resistivity), i.e.,

*α* =*F* ( *f* ,*ε* , *ρ* ) . As can be seen from

215 equation (1), *ε* (relatively dielectric constant) is a function of ƒ (frequency), i.e.,
216 *f* =*F* -1 (*ε* ). From Eq. (5), it is found that ƒ (frequency) is a function of *α* (attenuation
217 217

coefficient), i.e.,

*α* =*F* -1 ( *f* ) . Thus, the propagation attenuation law of radar waves in

218 218

coal is given by

⎧

⎪

⎪

*a*=2π*f* ⎡ ε

⎣ 2

⎢ (

-1)⎤1 2

1 + (1 2π*f* ρε)^2^

⎪	⎧*C'*

⎥⎦

*f* 3 + *C' f* 2 + *C'*

*f* + *C'*

, (*f* ∈[100*MHz,*500*MHz*])

⎪ *a*= ⎨

13	12	11	10

219 219

*C'	f* 2 + *C'	f* + *C'*	,	(*f* ∈(500*MHz,*1*.*0*GHz*])

(8)

⎨	⎩	22	21	20

⎪	ε=*C*3 *f* 3 + *C*2 *f* 2 + *C*1 *f* + *C*0 *,*( *f* ∈[100*MHz,*1*.*0*GHz*])

⎪

⎪	⎧*C*13 *f* 3 + *C*12 *f* 2 + *C*11 *f* + *C*10 ,	(*f* ∈[100*MHz,*500*MHz*])

⎪ρ= ⎨

*C f* 2 + *C*

*f* + *C*

,	(*f* ∈(500*MHz,*1*.*0*GHz*])

⎩	⎩	22

21	20

220 where *α* is the attenuation coefficient of radar wave propagation in coal. *f* is the

*C*

,

*ij*

221 221

frequency. *ε*	is	a	relative dielectric constant.	*ρ*	is the resistivity.

- *C_i_* ,

222 222

*Cij* ,(*i*, *j* ∈[0,1, 2,3])

are the coefficients of the corresponding function, respectively.

223 It was necessary to analyze the characteristics of waves to determine whether
224 there was anyone behind the coal when using radar for rescue. The characteristics of
225 waves were determined by laws of refraction and reflection, that is, Eq. (9), consisting
226 226
227 227

of Eqs. (6) and (7).

⎧

⎪*Rij* =

⎪

⎨

⎪*T* =

(9)

⎪ *ij*

⎩

228 228

*εi* −	*ε j*

*εi* +	*ε j*

2 *εi*

*εi* + *ε j*

where *ε i*

and *ε _j_*

are the relative dielectric constants of substance *i* and *j*,

229 229

respectively.

*Rij*

and

*Tij*

are the reflection and refraction coefficients, respectively.

230 According to the relationship between distance, speed and time, the distance
231 between the radar and person in distress can be calculated according to the following
232 formula.
233 233

⎧

⎪

⎡ *ε*

⎨*v=* ⎢ (

*L*=*v* ⋅ *t*

⎤−1 2 (10)

1 + (1 / 2*π f ερ* )^2^

+ 1)

⎥

⎩⎪	⎣ ^2^	⎦

234 where *v* is the propagation speed of radar waves in coal, in m/s.
235 The equations formed by the combination of Eqs. (8), (9), and (10) were the
236 control equations for the propagation of radar waves in coal.

⎧	*a*=2π*f* ⎡ ε

⎪	⎢

(

⎪	⎣ 2

-1)⎤1 2

⎪	⎧*C' f* 3 + *C' f* 2 + *C'*

1 + (1 2π*f* ρε)^2^

⎥⎦

*f* + *C'*

*,*(*f* ∈[100*MHz,*500*MHz*])

⎪*a*= ⎨

13	12	11	10

⎪	⎩	*C'*

*f* 2 + *C'*

*f* + *C'*

*,*(*f* ∈(500*MHz,*1*.*0*GHz*])

22	21	20

⎪	ε=*C*3 *f* 3 + *C*2 *f* 2 + *C*1 *f* + *C*0 *,*( *f* ∈[100*MHz,*1*.*0*GHz*])

⎪	⎧*C f* 3 + *C f* 2 + *C f* + *C ,*(*f* ∈[100*MHz,*500*MHz*])

*C	f*	+ *C	f* + *C	,*(*f* ∈ 500*MHz,*1*.*0*GHz* )(	]

237 237

⎪ρ= ⎨

⎪	⎩

⎨

⎪

⎪

⎪

⎪

⎪

⎪

13	12	11	10

2

22	21	20

ε*i* −	ε *j*

ε*i* +	ε *j*

2 ε*i*

ε*i* + ε *j*

*Rij* =

*Tij* =

(11)

⎪	*v=* ⎡ ε (

⎢

⎪

⎤−1 2

1 ⎥

1 + *(*1 */* 2π*f* ερ *)*^2^

+ )

238 238

⎪	⎣ 2

⎪⎩

3. Results and discussion

⎦

*L*=*v* ⋅ *t*

239 The equations (11) could be solved using GprMax code. GprMax is a simulation
240 software based on FDTD theory, which was developed by Dr. Antonis Giannopoulos
241 of the University of Edinburgh [44]. The GprMax has been widely used for simulation
242 and modelling of radar waves propagation in such GPR applications as underground
243 pipeline surveys, roadbed inspections, ancient building surveys, military surveys,
244 quality inspections, and hidden grave surveys [45-49]. The simulation calculation
245 process is shown in Fig. 10. The simulated physical model is shown in Fig. 11(Ⅰ).
246 As shown in Fig. 11(Ⅱ), the thickness of a coal body between a trapped person
247 and radar was L1, the distance between a person and coal wall was L2, the width of the
248 model was L3, the length of model was L4, and the size of a human body is 1.6 m ×0.4
249 m. The specific settings of different models are shown in Table 7. Then, the simulated
250 data was post-processed using Matlab, and the result is shown in Fig. 11(Ⅲ).
251 It was assumed that coal was a semi-infinite continuous space with homogeneous
252 isotropy. The dielectric constant of a human was 50 [50]. The dielectric constant of air
253 was 1, the antenna stepping distance was 0.06 m, and the transmitting and receiving
254 antenna spacing was 0.065 m. The number of measuring lines was 115, the spatial
255 grid step was 0.005 m×0.005 m, the permeability of all media in the model was 1.0,
256 the boundary condition was a perfect matching layer (PML), the excitation function of
257 the radar was Ricker, and the center frequency was 500 MHz. The partial electrical
258 parameters of the model were set as shown in Table 8. Due to the large number of
259 graphs of simulation results, only lignite was taken as an example for analysis here.
260 The results are shown in Fig. 12.
261 From Fig. 12, we found that the amplitudes of return wave from the interface
262 between the coal pillar and roadway in seven models were 12.51, 5.48, 2.56, 1.23,
263 0.63, 0.30, and 0.15 mV/m, and the return amplitude of the human body is -20.63.

264	-10.24, -5.30, -2.72, -1.37, -0.69, and -0.35 mV/m. It was not difficult to find that the

265 intensity of amplitude gradually decreased with the increase of L1 (thickness of the
266 coal pillar). The larger the L1, the longer the electromagnetic wave travels in coal, and
267 the loss of electromagnetic wave energy also increased, so the intensity of the target
268 return wave amplitude was gradually smaller. By fitting, it was found that the
269 amplitude of the human body wave decreases with the increase of L1 as an
270 exponential function (as shown in Fig. 13). The effective propagation distance of
271 radar waves in coal was between 6 and 8 m for transducer with central frequency 500
272 MHz.
273 4. Conclusions
274 Dielectric properties of five different coals in from Chinese deposits were
275 studied using the alternating current impedance method and the Concept-80
276 broadband	dielectric	Spectrum	test	system	for	electromagnetic	waves	with
277 frequencies ranging from 100 MHz to 1000 MHz, for the first time, to the best of our
278 knowledge. The propagation process of radar waves in coal was analyzed.
279 The experimental results showed that the dielectric constants of five different
280 coals followed similar rules. The values of relative dielectric constant of coals
281 decreased first and then increased with frequency. At the same frequency, the order of
282 values of the relative permittivity of coal from high to low was lean coal, lignite, gas
283 coal, long flame coal, and anthracite. The values of specific resistivity of coal
284 decreased considerably with increasing frequency, but there was observed an upward
285 bump around frequency 500 MHz.
286 The key parameters of radar waves propagating in coal were analyzed. The
287 propagation velocity values of radar waves in different rank coals and the reflection
288 and refraction coefficients values at different types of interfaces were calculated. The
289 attenuation coefficient of radar waves in five kinds of coals followed a similar rule.
290 With 500 MHz as the dividing point, the relation between attenuation coefficient
291 of radar waves and frequency was divided into two parts. In part I, the frequency was
292 a cubic function of the attenuation coefficient. The attenuation coefficient initially
293 increases to a certain point and then decreases with increasing of frequency values. In
294 part II, the frequency was a quadratic function of the attenuation coefficient, and the
295 values of attenuation coefficient increased with frequency increase. A set of equations
296 for the propagation of radar waves in coal were established, based on the above
297 researches. By analyzing the governing equations, it was found that the effective
298 penetration distance of radar wave with dominant frequency 500MHz in coal ranges is
299 299
300 300

between 6 and 8 m.

301 Acknowledgements
302 Funding: The project was supported by the National Natural Science Foundation of
303 China (grant numbers 51904234 and 51974240) and the Key R & D Program of
304 Shaanxi Provincial (grant number 2017ZDCXL-GY-01-02-03).

References

1. Guo L, Zhai M, Wang Z, Zhang Y, Dong P. Comprehensive coal quality index for evaluation of coal agglomeration characteristics, Fuel 2018; 231(12): 379-386.
2. Song H, Liu J, Xue F, Cheng F. The application of ultra-fine fly ash in the seal coating for the wall of underground coal mine, Advanced Powder Technology 2016; 27: 1645-1650.
3. Diniz DA, Prasad P, Pagan RJ. Modern environmental improvement pathways for the coal power generation industry in Australia. Process Safety and Environmental Protection 2004; 82: 191-199.
4. Warmuzinski K. Harnessing methane emissions from coal mining. Process Safety and Environmental Protection 2008; 86: 315-320.
5. British Petroleum (BP), Statistical Review of World Energy, 2015. http://www.bp.

ucom/statistical review.

6. Wang D. Thermodynamic disaster in coal mine and its characteristics. Journal of China Coal Society 2018; 43(1): 137-142.
7. Wang K, Jiang S, Ma X, Wu Z, Shao H, Zhang W, et al. Information fusion of plume control and personnel escape during the emergency rescue of external-caused fire in a coal mine.

Process Safety and Environmental Protection 2016; 103: 46-59.

8. Ken B. Buried alive, but carbide drills at core of Chile miners' rescue. Metal Powder Report 2010; 65(7): 7-10.
9. Yang T, Du B. Construction Technology of Large Diameter rescue borehole in Pingyi gypsum mine disaster of Shandong, Exploration Engineering ( Rock & Soil Drilling and Tunneling) 2017, 44(5): 19-23. (In Chinese)
10. Wen H, Zhang D, Zheng X-Z. Research Progress and Trend of Life Detection Technology of Drilling and Rescue in Mine. Safety in Coal Mines 2017, 48(9): 85-88. (In Chinese)
11. Li Z, Li W, Lv H, Zhang Y, Jing X, Wang J. A novel method for respiration-like clutter cancellation in life detection by dual-frequency IR-UWB radar, IEEE Transactions on Microwave Theory and Techniques 2013; 61 (5): 2086-2092.
12. Hu W, Zhao Z, Wang Y, Zhang H, Lin F. Noncontact accurate measurement of cardiopulmonary activity using a compact quadrature Doppler radar sensor, I IEEE Transactions on Biomedical Engineering 2014; 61 (3): 725-735.
13. Chen K. Huang Y, Zhang J, Norman A. Microwave life-detection systems for searching human subjects under earthquake rubble or behind barrier, IEEE Transactions on Biomedical Engineering 2000; 47 (1): 105-114.
14. JalaliBidgoli F, Moghadami S, Ardalan S. A compact portable microwave lifedetection device for finding survivors. IEEE Embedded Systems Letters 2016; 8(1): 10-13.
15. Liang X, Zhang H, Fang G, Ye S, Gulliver T. An improved algorithm for throughwall target detection using ultra-wideband impulse radar. IEEE Access 2017; (99): 22101-22118.
16. Guan S, Rice JA, Li C, Gu C. Automated dc offset calibration strategy for structural health monitoring based on portable cw radar sensor. IEEE Transactions on Instrumentation and Measurement 2014; 63(12): 3111-3118.
17. Wang F, Horng TS, Peng K, Jau J, Li J, Chen C. Single-antenna Doppler radars using self and mutual injection locking for vital sign detection with random body movement cancellation. IEEE Transactinos on Microwave Theory and Techniques 2011; 59 (12): 3577-3587.
18. Liang X, Zhang H, Lyu T, Xu L, Cao C, Gulliver TA. Ultra-wide band impulse radar for life

detection using wavelet packet decomposition. Physical Communication 2018; 29:31-47.

19. Fan W, Jia C-Y, Hu W, Yang C, Liu L, Zhang X, et al. Dielectric properties of coals in the low-terahertz frequency region. Fuel 2015; 162: 294-304.
20. Fan W, Chang T, Liu L, Cui H. Dielectric properties of coal in the terahertz frequency region of 100–500 GHz. Fuel 2017; 188: 246-253.
21. Qin F, Wei G. Measurement with Sweeping Frequency for Complex Permittivity Based on VNA. Journal of Astronautic Metrology and Measurement 2011; 31(3): 27-30.
22. Brach I, Giuntini JC, Zanchetta JC. Real part of the permittivity of coals and their rank. Fuel 1994; 73(5): 738-741.
23. Marland S, Merchant A., Rowson N. Dielectric properties of coal. Fuel 2001; 80: 1839-1849.
24. Valentina VZ. Influence of polyethylene terephthalate on the carbonisation of bituminous coals and on the modification of their electric and dielectric properties. Fuel; 2006, 85:1652-1665.
25. Wang Q, Zhang X, Gu F. Investigation on interior moisture distribution inducing dielectric anisotropy of coals. Fuel processing technology 2005; 89:633-641.
26. Peng Z, Lin X, Li Z, Hwang J, Kim B, Zhang Y, et al. Dielectric characterization of Indonesian low-rank coal for microwave processing. Fuel processing technology 2017; 156: 171-177.
27. Tao X, Tang L, Xie M, He H, Xu N, Feng L, et al. Dielectric properties analysis of sulfur-containing models in coal and energy evaluation of their sulfur-containing bond dissociation in microwave field. Fuel 2016; 181: 1027-1033.
28. Xu L, Liu H, Jin Y, Fan B, Qiao X, Jing B. Structural order and dielectric properties of coal chars. Fuel 2014; 137: 164-171.
29. Liu H, Xu L, Jin Y, Fan B, Qiao X, Yang Y. Effect of coal rank on structure and dielectric

properties of chars. Fuel 2015; 153: 249-256.

30. Emslie AG, Lagace RL. Propagation of low and medium frequency radio waves in a coal seam.

Radio Science 1976; (11): 253-261.

31. Hill DA. Radio propagation in a coal seam and the inverse problem. Journal of Research of the National Bureau of Standards 1984; (89): 385-394.
32. Hill DA. Electromagnetic detection of long conductors in tunnels. IEEE Trans 1985; 33(4).
33. Greenfield RJ,Wu ST. Electromagnetic wave propagation in disrupted coal seams. Geophysics 1991; 56(10): 1571-1577.
34. Luo Y, Stolarczyk LG, Peng SD. Demonstration of the advanced radio imaging method (rim): ground truth results for three-dimensional imaging of coal seams. Sme Meeting 2004.
35. Stolarczyk LG, Fry RC. Radio imaging method (RIM) or diagnostic imaging of anomalous geologic structures in coal seam waveguides. Transactions of Society for Mining, Metallurgy, and Exploration, Inc. 1990; (288): 1806-1814.
36. Stolarczyk LG. Electromagnetic seam wave mapping of roof rock conditions across a longwall panel. 18th International Conference on Ground Control in Mining, Aug 3-5.1999.
37. Stolarczyk LG, Peng SS, Luo Y. Imaging ahead of Mining with Radio Imaging Method (RIM-IV) Instrumentation and Three-dimensional Tomography. Software Proceedings of 22nd International Conference on Ground Control in Mining 2003, Morgantown, WV, 136-143.
38. Zhu H, Xin M, Chang M, Liu D. Research progress on measuring technology of coal dielectric

constant. Coal Science Technology 2016; 44(9): 6-12．

39. Singclair DC, West AR. Impedance and modulus spectroscopy of semiconducting BaTiO3

showing positive temperature coefficient of resistance. Journal of Applied. Physics 1989; 66(8): 3850-3856.

40. Feng X, Chen J, Li N, Cui H, Liu J. Study on electromagnetic properties of anthracite and soft coal in microwave field. Study on electromagnetic properties of anthracite 2007; 38(5): 405-411. (In Chinses)
41. Zhou, B. and Fullagar, P.K., 2001, Delineation of sulphide ore-zones by borehole radar tomography at Hellyer Mine, Australia: Journal of Applied Geophysics, 47, No 3-4, pp.261-269.
42. Qi C. Ground penetrating radar signal processing in advanced detection in coal seam, Xi’an university of science and technology 2014. (In Chinese)
43. Liu X. Research the propagation rules of middle-high frequency electromagnetic wave in coal medium. China University of Mining and Technology 2012.
44. Giannopoulos A. Modelling ground penetrating radar by GprMax. Construction and Building Materials 2005; 19: 755-762.
45. Núñez-Nieto X, Solla M, Novob A, Lorenzo H. Three-dimensional ground-penetrating radar

methodologies for thecharacterization and volumetric reconstruction of underground tunneling. Construction and Building Materials 2014; 71: 551-560.

46. Adam DB, Jamie K, Pringle. Semblance analysis to assess GPR data from a five-year forensic study of simulated clandestine graves. Journal of Applied Geophysics 2016; 125: 17-44.
47. Lopera O, Milisavljevic N. Prediction of the effects of soil and target properties on the antipersonnel landmine detection performance of ground- penetrating radar: A Colombian case study. Journal of Applied Geophysics 2007; 63: 13-23.
48. Shangguan P, Imad L. AQ, Lahouar S. Pattern recognition algorithms for density estimation of asphalt pavement during compaction: a simulation study. Journal of Applied Geophysics 2014; 107: 8-15.
49. Jaw SW, Hashim M. Locational accuracy of underground utility mapping using ground penetrating radar. Tunnelling and Underground Space Technology Incorporating Trenchless Technology Research 2013; 35: 20-29.
50. Dogaru T, Nguyen L, Le C. Computer models of the human body signature for sensing through the wall radar applications. DTIC Document Adelphi MD 2007.
51. Lee JH, Wang W. Characterization of snow cover using ground penetrating radar for vehicle trafficability–Experiments and modeling. Journal of Terramechanics 2009; 46: 189-202.

Table captions

**Table 1** Proximate and elemental analysis of coal.

**Table 2** Coefficients of fitting equation.

**Table 3** Coefficients of fitting equation.

**Table 4** Propagation speed of radar waves in coal (m/s).

**Table 5** Coefficients of fitting equation. **Table 6** Reflection and refraction coefficients. **Table 7** Size of model (m).

**Table 8** Dielectric characterization of Coal.

Coal

|  | Mad | Aad | Vad | FCad | C | H | O | N | S | (%) |
| --- | --- | --- | --- | --- | --- | --- | --- | --- | --- | --- |
| 1# | 8.80 | 36.62 | 24.68 | 29.90 | 38.94 | 4.01 | 22.00 | 1.15 | 0.27 | 10.11 |
| 2# | 5.77 | 11.93 | 32.32 | 49.98 | 64.86 | 5.63 | 19.20 | 1.73 | 0.58 | 13.34 |
| 3# | 2.33 | 15.92 | 35.45 | 46.30 | 63.92 | 4.85 | 17.40 | 2.37 | 0.51 | 6.16 |
| 4# | 0.53 | 24.49 | 12.95 | 62.03 | 62.51 | 3.76 | 17.00 | 1.61 | 3.41 | 9.19 |
| 5# | 1.20 | 8.84 | 7.85 | 82.11 | 81.82 | 3.79 | 4.90 | 0.85 | 1.49 | 16.20 |
| **Table 2** Coefficients of fitting equation. | | | | | | | | | | |

**Table 1** Proximate and elemental analysis of coal.

Proximate analysis (%)	Elemental analysis (%)	Porosity

| Coals | C3 | C2 | C1 | C0 | R^2^（%） |
| --- | --- | --- | --- | --- | --- |
| 1# | -9.24×10^-10^ | 1.84×10^-6^ | -9.14×10^-4^ | 3.62 | 98.98 |
| 2# | -5.82×10^-10^ | 1.17×10^-6^ | -5.43×10^-4^ | 3.07 | 98.77 |
| 3# | -6.34×10^-10^ | 1.28×10^-6^ | -5.96×10^-4^ | 3.35 | 99.21 |
| 4# | -7.90×10^-10^ | 1.52×10^-6^ | -6.58×10^-4^ | 3.78 | 99.40 |
| 5# | -6.42×10^-10^ | 1.34×10^-6^ | -6.86×10^-4^ | 3.05 | 98.49 |

**Table 3** Coefficients of fitting equation.

| Partion | Coefficient | 1# | 2# | 3# | 4# | 5# |
| --- | --- | --- | --- | --- | --- | --- |
|  | C13 | -8.00×10^-5^ | -5.45×10^-5^ | -4.96×10^-5^ | -4.08×10^-5^ | -7.48×10^-5^ |
|  | C12 | 0.10 | 0.07 | 0.06 | 0.05 | 0.10 |
| Ⅰ | C11 | -43.06 | -30.19 | -27.52 | -23.03 | -40.16 |
|  | C10 | 6.13×10^3^ | 4.45×10^3^ | 4.06×10^3^ | 3.43×10^3^ | 5.78×10^3^ |
|  | R2 | 98.40 % | 99.25 % | 99.25 % | 99.24 % | 98.95 % |
| C22 | | 1.28×10^-3^ | 9.50×10^-4^ | 8.74×10^-4^ | 7.99×10^-4^ | 1.12×10^-3^ |
| C21 | | -2.36 | -1.81 | -1.66 | -1.48 | -2.22 |
| C20 | | 1.21×10^3^ | 9.60×10^3^ | 8.80×10^2^ | 7.86×10^2^ | 1.10×10^3^ |

Ⅱ

R^2^	97.94 %	99.11 %	98.84 %	98.58 %	98.91 %

**Table 4** Propagation speed of radar waves in coal (m/s).

| Frequency (MHz) | 1# | 2# | 3# | 4# | 5# |
| --- | --- | --- | --- | --- | --- |
| 100 | 15.70×10^7^ | 17.23×10^7^ | 16.14×10^7^ | 15.53×10^7^ | 17.32×10^7^ |
| 200 | 16.01×10^7^ | 17.29×10^7^ | 16.64×10^7^ | 15.57×10^7^ | 17.43×10^7^ |
| 300 | 16.05×10^7^ | 17.32×10^7^ | 16.90×10^7^ | 15.59×10^7^ | 17.46×10^7^ |
| 400 | 16.03×10^7^ | 17.29×10^7^ | 16.64×10^7^ | 15.55×10^7^ | 17.46×10^7^ |
| 500 | 16.01×10^7^ | 17.23×10^7^ | 16.14×10^7^ | 15.53×10^7^ | 17.39×10^7^ |
| 600 | 15.94×10^7^ | 17.20×10^7^ | 16.64×10^7^ | 15.45×10^7^ | 17.37×10^7^ |
| 700 | 15.87×10^7^ | 17.12×10^7^ | 16.15×10^7^ | 15.38×10^7^ | 17.29×10^7^ |
| 800 | 15.81×10^7^ | 17.66×10^7^ | 16.42×10^7^ | 15.32×10^7^ | 17.26×10^7^ |
| 900 | 15.76×10^7^ | 17.14×10^7^ | 16.29×10^7^ | 15.30×10^7^ | 17.17×10^7^ |
| 1000 | 15.74×10^7^ | 16.84×10^7^ | 16.26×10^7^ | 15.26×10^7^ | 17.14×10^7^ |

**Table 5** Coefficients of fitting equation.

| Part | Coefficient | 1# | 2# | 3# | 4# | 5# |
| --- | --- | --- | --- | --- | --- | --- |
|  | C'13 | -8.31×10^-14^ | -6.70×10^-14^ | -7.01×10^-14^ | -7.58×10^-14^ | -6.91×10^-14^ |
|  | C'12 | 6.36×10^-11^ | 4.95×10^-11^ | 5.17×10^-11^ | 5.55×10^-11^ | 5.21×10^-11^ |
| Ⅰ | C'11 | -1.03×10^-8^ | -6.20×10^-9^ | -6.46×10^-9^ | -6.90×10^-9^ | -7.25×10^-9^ |
|  | C'10 | 4.71×10^--7^ | 1.97×10^-7^ | 2.05×10^-7^ | 2.25×10^-7^ | 2.52×10^-7^ |
|  | R2 | 99.36% | 99.72% | 99.72% | 99.71% | 99.69% |
| C'22 | | -3.31×10^-12^ | -1.31×10^-12^ | -2.22×10^-12^ | -4.63×10^-12^ | -6.46×10^-11^ |
| C'21 | | 8.44×10^-9^ | 6.78×10^-9^ | 7.90×10^-9^ | 1.04×10^-8^ | 6.12×10^-9^ |
| C'20 | | -2.61×10^-6^ | 2.04×10^-6^ | -2.34×10^-6^ | -2.94×10^-6^ | -1.97×10^-6^ |

Ⅱ

R^2^	98.88%	99.52%	99.37%	98.66%	99.58%

**Table 6** Reflection and refraction coefficients.

(1)

| Coal | Type  air→coal coal→air air→person person→air coal→person person→coal | | | | | | |
| --- | --- | --- | --- | --- | --- | --- | --- |
| 1# *Rij* -0.31 0.31 -0.75 0.75 | | | | | | -0.58 | 0.58 |
| *Tij* | | 0.698 | 1.31 | 0.25 | 1.75 | 0.42 | 1.58 |
| 2# *Rij* | | -0.27 | 0.27 | -0.75 | 0.75 | -0.60 | 0.60 |
| *Tij* | | 0.28 | 1.27 | 0.25 | 1.75 | 0.40 | 1.60 |
| 3# *Rij* | | -0.29 | 0.29 | -0.75 | 0.75 | -0.59 | 0.59 |
| *Tij* | | 0.71 | 1.29 | 0.25 | 1.75 | 0.41 | 1.59 |
| 4# *Rij* | | -0.32 | 0.32 | -0.75 | 0.75 | -0.57 | 0.57 |
| *Tij* | | 0.68 | 1.32 | 0.25 | 1.75 | 0.43 | 1.57 |
| 5# *Rij* | | -0.27 | 0.27 | -0.75 | 0.75 | -0.61 | 0.67 |
| *Tij* | | 0.737 | 1.27 | 0.25 | 1.75 | 0.39 | 1.67 |
| **Table 7** Size of model (m). | | | | | | | |

(2)

(3)

(4)

(5)

(6)

| Model | M1 | M2 | M3 | M4 | M5 | M6 | M7 |
| --- | --- | --- | --- | --- | --- | --- | --- |
| L1 | 2 | 3 | 4 | 5 | 6 | 7 | 8 |
| L2 | 1.3 | 1.3 | 1.3 | 1.3 | 1.3 | 1.3 | 1.3 |
| L3 | 3.3 | 4.3 | 5.3 | 6.3 | 7.3 | 8.3 | 9.3 |
| L4 | 10 | 10 | 10 | 10 | 10 | 10 | 10 |
| **Table 8** Dielectric characterization of Coal**.** | | | | | | | |

| Coal | 1# | 2# | 3# | 4# | 5# |
| --- | --- | --- | --- | --- | --- |
| ε | 3.51 | 3.03 | 3.30 | 3.73 | 2.99 |
| ρ (Ω·m) | 328.95 | 290.07 | 267.78 | 242.41 | 318.53 |

Figure captions

**Fig. 1.** Schematic concept of and application of borehole GPR method for location of miner trapped in underground tunnel in coal mine.

**Fig. 2.** Flow chart of processing test of coal samples.

**Fig. 3.** Concept-80 Broadband Dielectric Spectrum Test System.

**Fig. 4.** Changes of the relative dielectric permittivity with frequency for various coals.

**Fig. 5.** Variations of various coals resistivity with frequency.

**Fig. 6.** Changes of attenuation coefficient with frequency.

**Fig. 7.** Schematic diagram of types of reflection and refraction.

**Fig. 8.** Simulation calculation flow.

**Fig. 9.** Geometrical model of rock strata applied in numerical simulation.

**Fig. 10.** A plot of signal amplitudes versus travel time.

**Fig. 11.** Relationship	(or a plot) between amplitude of a radar signal reflected from a person and a coal seam thickness L1.

**Fig. 1.** Schematic concept of and application of borehole GPR method for location of miner trapped in underground tunnel in coal mine.

A is a three-dimensional diagram of rescue drilling during the tunnel collapse；B is a schematic

diagram of the drilling offset.

**Slice of coal**

**Tableting**

**machine**

**Fig. 2.** Flow chart of processing test of coal samples.

**Fig. 3.** Concept-80 Broadband Dielectric Spectrum Test System.

3.64	3.14

(A)

1#

Fitting line

(B)

2#

Fitting line

3.62	3.12

3.60	3.10

Relative dielctric permittivity

Relative dielctric permittivity

3.58	3.08

3.56	3.06

3.54	3.04

3.52	3.02

3.50

3.48

200	400	600	800	1000

Frequency (MHz)

3.00

2.98

200	400	600	800	1000

Frequency (MHz)

3.42	3.88

(C)

3#

Fitting line

3.40

3.38

Relative dielctric permittivity

3.36

3.34

3.32

3.30

3.28

3.26

3.24

3.86

3.84

Relative dielctric permittivity

3.82

3.80

3.78

3.76

3.74

3.72

3.70

3.68

4#

Fitting line

(D)

200	400	600	800	1000

Frequency (MHz)

200	400	600	800	1000

Frequency (MHz)

3.08

3.06

3.04

Relative dielctric permittivity

3.02

3.00

2.98

3.9

3.8

(E)

5#

Fitting line

1#

2#

3#

4#

5#

(F)

3.7

Relative dielctric permittivity

3.6

3.5

3.4

3.3

3.2

2.96

2.94

200	400	600	800	1000

Frequency (MHz)

3.1

3.0

2.9

200	400	600	800	1000	1200

Frequency (MHz)

**Fig. 4.** Changes of the relative dielectric permittivity with frequency for various coals

**A**: lignite; B: long flame coal; C: gas coal; D: lean coal; E: anthracite; F: various coals.

1400

1200

1000

Resistivity (Ω*m)

800

600

1000

750

(**B**)

y=-5.45×10^-5^x^3^+0.07x^2^-30.19x+4.45×10^3^

R^2^=99.25%

y=9.50×10^-4^x^2^-1.81x+9.60×10^2^

R^2^=99.11%

Part **Ⅰ**

Part **Ⅱ**

Resistivity (Ω*m)

500

400

200

250

0

(**A**)

y=-8.00×10^-5^x^3^+0.10x^2^-43.06x+6.13×103 R^2^=98.40%

y=1.28×10^-3^x^2^-2.36x+1.21×103 R^2^=97.94%

Part **Ⅰ**

Part **Ⅱ**

200	400	600	800	1000

Frequency (108 Hz)

0

200	400	600	800	1000

Frequency (MHz)

900

800

700

600

Resistivity (Ω*m)

500

400

300

800

700

(**D**)

y=-4.08×10^-5^x^3^+0.05x^2^-23.03x+3.43×10^3^ R^2^=99.24%

y=7.99×10^-4^x^2^-1.48x+7.86×10^2^ R^2^=98.58%

Part **Ⅰ**

Part **Ⅱ**

600

500

Resistivity (Ω*m)

400

300

200

100

0

200	400	600	800	1000

(**C**)

y=-4.96×10^-5^x^3^+0.06x^2^-27.52x+4.06×10^3^ R^2^=99.25%

y=8.74×10^-4^x^2^-1.66x+8.80×10^2^

R^2^=98.84%

Part **Ⅰ**

Part **Ⅱ**

Frequency (10^8^ Hz)

200

100

200	400	600	800	1000

Frequency (MHz)

1200

1000

1200

1000

(**F**)

1#

2#

3#

4#

5#

Part **Ⅰ**

Part **Ⅱ**

800	800

(**E**)

y=-7.48×10^-5^x^3^+0.10x^2^-40.16x+5.78×10^3^ R^2^=98.95%

y=1.12×10^-3^x^2^-2.22x+1.10×10^3^

R^2^=98.91%

Part **Ⅰ**

Part **Ⅱ**

Resistivity (Ω*m)

Resistivity (Ω*m)

600	600

400	400

200	200

0

200	400	600	800	1000

Frequency (MHz)

0

200	400	600	800	1000

Frequency (MHz)

**Fig. 5.** Variations of various coals resistivity with frequency.

**A**: lignite; B: long flame coal; C: gas coal; D: lean coal; E: anthracite; F: various coals.

2.5x10^-6^

Attenuation coefficient (Np/m)

2.0x10^-6^

1.5x10^-6^

1.0x10^-6^

5.0x10^-7^

0.0

3.5x10^-6^

3.0x10^-6^

(**A**)

y=-8.31e^-14^x^3^+6.36e^-11^x^2^-1.03e^-8^x+4.71e^-7^

R^2^=99.36%

y=-3.31e^-12^x^2^+8.44e^-9^x-2.61e^-6^ R^2^=98.88%

Part**Ⅰ**

Part **Ⅱ**

(**B**)

y=-6.7e^-14^x^3^+4.95e^-11^x^2^-6.2e^-9^x+1.97e^-7^ R^2^=99.72%

y=-1.31e^-12^x^2^+6.78e^-9^x+2.04e^-6^ R^2^=99.52%

Part**Ⅰ**

Part **Ⅱ**

Attenuation coefficient (Np/m)

2.5x10^-6^

2.0x10^-6^

1.5x10^-6^

1.0x10^-6^

5.0x10^-7^

0.0

-5.0x10^-7^

| 0 | 200 | 400 | 600 | 800 | 1000 |  | 0 | 200 | 400 | 600 | 800 | 1000 |
| --- | --- | --- | --- | --- | --- | --- | --- | --- | --- | --- | --- | --- |
|  |  | Frequency | (MHz) |  |  |  |  |  | Frequency | (MHz) |  |  |

3.5x10^-6^

3.0x10^-6^

Attenuation coefficient (Np/m)

3.0x10^-6^

2.5x10^-6^

(**C**)

y=-7.01e^-14^x^3^+5.17e^-11^x^2^-6.46e^-9^x+2.05e^-7^ R^2^=99.72%

y=-2.22e^-12^x^2^+7.90e^-9^x-2.34e^-6^ R^2^=99.37%

Part**Ⅰ**

Part **Ⅱ**

(**D**)

y=-7.58e^-14^x^3^+5.55e^-11^x^2^-6.90e^-9^x+2.25e^-7^ R^2^=99.71%

y=-4.63e x +1.04e x-2.94e R^2^=98.66%

-12	2	-8	-6

Part**Ⅰ**

Part **Ⅱ**

Attenuation coefficient (Np/m)

2.5x10^-6^

2.0x10^-6^

1.5x10^-6^

1.0x10^-6^

5.0x10^-7^

0.0

-5.0x10^-7^

0

200	400

600	800	1000

2.0x10^-6^

1.5x10^-6^

1.0x10^-6^

5.0x10^-7^

0.0

0

200	400

600	800	1000

Frequency (MHz)	Frequency (MHz)

3.5x10^-6^

(**E**)

y=-6.91e^-14^x^3^+5.21e^-11^x^2^-7.25e^-9^x+2.52e^-7^ R^2^=99.69%

y=-6.46e^-11^x^2^+6.12e^-9^x-1.97e^-6^ R^2^=99.58%

Part**Ⅰ**

Part **Ⅱ**

3.0x10^-6^

Attenuation coefficient (Np/m)

2.5x10^-6^

2.0x10^-6^

1.5x10^-6^

1.0x10^-6^

3.5x10^-6^

3.0x10^-6^

1#

2#

3#

4#

5#

(**F**)

① ②

Ⅰ

Ⅱ

Attenuation coefficient (Np/m)

2.5x10^-6^

2.0x10^-6^

1.5x10^-6^

1.0x10^-6^

5.0x10^-7^

0.0

-5.0x10^-7^

| 0 | 200 | 400 | 600 | 800 | 1000 | 200 | 400 | 600 | 800 | 1000 |
| --- | --- | --- | --- | --- | --- | --- | --- | --- | --- | --- |
|  |  | Frequency | (MHz) |  |  |  |  | Frequency (MHz) |  |  |

5.0x10^-7^

0.0

-5.0x10^-7^

**Fig. 6.** Changes of attenuation coefficient with frequency. **A**: lignite; B: long flame coal; C: gas coal; D: lean coal; E: anthracite; F: various coals.

**Fig. 7.** Schematic diagram of types of reflection and refraction.

Radar waves travel from one object to another. (1): air→coal; (2): coal→air; (3): air→person; (4): person→air; (5): coal→person; (6): person→coal.

Physical model

**Optimization**

GprMax Modeling

Time-Domain Gprmax signal

Define propagation control equations

Define parameter and setting

**Fig. 8.** Simulation calculation flow.

Solve

**Matlab**

Two-way travel time and reflection coeff.

**Optimization**

**(**Ⅲ**)**

**(**Ⅱ**)**

**(**Ⅰ**)**

**Fig. 9.** Geometrical model of rock strata applied in numerical simulation.

Ⅰ: Schematic of illustration of a disaster; Ⅱ: Schematic of simplified of physical model;Ⅲ: Display of physical model in GPR software.

1000

h

20

1

10

0

h_2_

-10

-20

2.0x10-8 2.5x10-8 3.0x10-8 3.5x10^-8^

Time (s)

B

C

(**M1**)

800

600

1000

800

h_1_

15	h

10

2

5

B

0

B

-5

-10

-15

3.5x10-8 4.0x10-8 4.5x10-8 5.0x10^-8^

Time (s)

C

(**M2**)

Amplitude (mv/m)

600

400	400

Amplitude (mv/m)

Amplitude (mv/m)

Amplitude (mv/m)

200	200

0	0

-200

-400

-600

1000

800

600

Amplitude (mv/m)

400

200

0

-200

-400

0.0

0.0

h_1_

10

h2

5

B

0

-5

5x10-8 Time (s)

C

6x10^-8^

(**M3**)

2.0x10^-8^	4.0x10^-8^	6.0x10^-8^	8.0x10^-8^

Time (s)

Amplitude (mv/m)

2.0x10^-8^	4.0x10^-8^	6.0x10^-8^	8.0x10^-8^

Time (s)

-200

-400

-600

1000

800

600

Amplitude (mv/m)

400

200

0

-200

-400

-600

0.0

0.0

h_1_

5.0

h_2_

2.5

B

0.0

-2.5

C

-5.0

6.0x10^-8^	6.5x10^-8^	7.0x10^-8^	7.5x10^-8^

Time (s)

(**M4**)

2.0x10^-8^	4.0x10^-8^	6.0x10^-8^	8.0x10^-8^

Time (s)

Amplitude (mv/m)

2.0x10^-8^	4.0x10^-8^	6.0x10^-8^	8.0x10^-8^

Time (s)

Amplitude (mv/m)

Amplitude (mv/m)

1000

800

600

400

Amplitude (mv/m)

200

0

-200

-400

-600

0.0	2.0x10^-8^	4.0x10^-8^	6.0x10^-8^	8.0x10^-8^	1.0x10^-7^

h_1_

2

h_2_

1

B

0

-1

C

-2

7x10^-8^

8x10^-8^

Time (s)

9x10^-8^

(**M5**)

Time (s)

1000

800

600

Amplitude (mv/m)

400

200

0

-200

-400

-600

0.0	2.0x10^-8^	4.0x10^-8^	6.0x10^-8^	8.0x10^-8^	1.0x10^-7^

h_1_

1.0

h_2_

0.5

B

0.0

-0.5

C

-1.0

8x10^-8^

9x10^-8^

1x10^-7^

Time (s)

(**M6**)

Time (s)

1000

h_1_

0.50

h_2_

0.25

B

0.00

-0.25

C

-0.50

9.5x10^-8^ 1.0x10^-7^ 1.1x10^-7^ 1.1x10^-7^ 1.2x10^-7^

Time (s)

(**M7**)

800

Amplitude (mv/m)

600

400

Amplitude (mv/m)

200

0

-200

-400

-600

0.0	2.0x10^-8^ 4.0x10^-8^ 6.0x10^-8^ 8.0x10^-8^ 1.0x10^-7^ 1.2x10^-7^

Time (s)

**Fig. 10.** A plot of signal amplitudes versus travel time.

Mx m are the thickness of the coal body between the radar and the trapped person.

M1: 2 m; M2: 3 m; M3: 4 m; M4: 5 m; M5: 6 m; M6: 7 m; M7: 8 m.

h*i* is two-way travel time of radar wave between two objects [52]. The meaning of B and C is

given in Fig. 9

**Fig. 11.** Relationship (or a plot) between amplitude of a radar signal reflected from a

person and a coal seam thickness L1.

Response to Reviewers

**Reply to Reviewers’ Comments**

**Journal:** PLOS ONE

**Manuscript ID:** PONE-D-19-29325

**Title:** “Propagation of Ground Penetrating Radar Waves in Chinese Coals”

**Authors:** Duo Zhang, Rui Tang, Hu Wen, Shixing Fan

| **Editor** | |
| --- | --- |
| **Comments to the Author** | **Response** |
| Thank you for submitting your manuscript to PLOS ONE. After careful consideration, we feel that it has merit but does not fully meet PLOS ONE’s publication criteria as it currently stands. Therefore, we invite you to submit a revised version of the manuscript that addresses the points raised during the review process. We would appreciate receiving your revised manuscript by Feb 04 2020 11:59PM. Two independent reports have been collected for your submission. Both the reports are generally  encouraging, but recommend major improvements. | We thank the anonymous reviewers for taking precious time to review our manuscript. We appreciate the reviewers giving us valuable comments that are helpful to improve our manuscript. Here we attempt to reply where and how we revised our manuscript in the light of reviewers’ comments. Revised contents of our manuscript and our replies are marked in red font. |

| **Reviewer #1** | |
| --- | --- |
| **Comments to the Author** | **Response** |
| The key contribution of this paper is that it reports the effects of EM wave frequencies on two fundamental parameters or properties: resistivity and dielectric permitivity for different rank coal samples. I am not quite sure one can claim the outcome/results of the lab testing of these parameter can be declared as “Law”. The claim of the Ultra-wideband radar wave is also arguable as the radar wave used is not necessarily ultra-wideband but the frequency was varied from 100 Mhz – 1000 MHz. Otherwise they would not be able to obtain the parameter variations with frequencies. Based on these measurements, it also derived the EM wave attenuation and speed variations with frequencies for different rank coal samples.  The speed is computed by using the simplified version of the wave speed formula (3). I suggest you may also compute with a more accurate formula such as (Zhou and Fullagar, 2001):  So that it may get different values to the simplified one. Surely the results may do not change much as the frequencies used are | Thanks for the reviewer’s comments. In the revised manuscript, we have modified the title as well as removed the word of “Law”. The controversial term UWB to GPR. As shown in Figure 1, we have developed a prototype radar device that can penetrate the coal seam to detect life information. During the research, we tested the dielectric constant of coal and analyzed the propagation of radar waves in the coal. At the initial stage of research, we have assumed some basic physical models. Experts from the China National Key R & D Project team tested our radar prototype at Jining No. 2 Coal Mine. The results show that the radar can penetrate 5m coal seam.    Fig. 1 Radar prototype for penetration detection |

| relatively high and the approximated version could provide sufficient accuracy.  One of the objectives of the paper is to use GPR for detection of mine trapped personnel after a coal mine disaster. With a frequency ranging from 100 MHz to 1000 MHz, I wonder what kind of penetration capability it will be although the paper claims, in Introduction, the GPR has “strong penetration”. The detection examples in Figures 11 & 12 are simplified too much with no practical use at all. At most, you only illustrate you can estimate the distances of reflection objects without knowing which direction they come from. Such ability is well illustrated in seismic and GPR textbooks. GPR is relatively matured technique. Is there any successful examples such as from controlled experiments in coal mine environment? I guess not. Even if you can receive the reflections from trapped personnel, you need to address the issue such as how to differentiate the human reflections from many other reflections from imhomogeinities of the strata, underground equipment and objects. If you cannot resolve this issue, your GPR measurements have no use to this particular applications.  Based on my above comments, I suggest the author concentrated  on the property measurement report rather than try also to address | Fig. 2 Schematic of radar structure  1: battery; 2: Control module of power; 3: Data transmission module; 4: CPU; 5: Data acquisition module; 6: Receiving antenna;  7: Transmitting antenna; 8: Gyro; 9: Transmission line; 10: Computer  We use a combination of the delay band intersection algorithm and the |
| --- | --- |

| the trapped human detection. Properly understanding the property of coal samples for different frequencies is a good contribution to the literature.  In addition, please improve your English expression of the paper to ensure communicate your finding to your reader effectively. This can be achieved by asking a native English speaker or English Editing services to help you before submit your paper. | gyroscope at the bottom of the radar to determine the direction of the target. The electrical parameters of strata and human beings are different. Also the directions of reflected waves are different. Therefore, it is possible to distinguish between reflected waves of people and strata.  Underground equipments and objects bring enormous interference waves during the detection process. We use the following method to screen effective waves. FIR low-pass filter is made use of eliminating direct waves and some background noises. The adaptive minimum mean square algorithm is used to eliminate periodic interference waves and narrowband waves in order to improve the signal-to-noise ratio.  The speed formula was simplified from the accurate formula suggested by the reviewer. We find that the difference between the simplified formula and theaccurate formula was very small. To the certain accuracy, the difference between the results of the two formulas can no longer be seen. For the rigor of the manuscript, we decided to accept the reviewer's opinion that the exact formula was chosen.  The article has been edited by academic editing with the native language. |
| --- | --- |

| **Reviewer #2** | |
| --- | --- |
| **Comments to the Author** | **Response** |
| General remarks: The paper presents data from laboratory testing of electrical properties of various coal samples taken from Chinese mines. Test were performed on a commercial Broadband Dielectric Spectrum Test System -Concept-80. It allows to determine the values of dielectric permittivity and specific resistivity of grinded coal samples subjected to the electromagnetic fields in wide frequency spectrum. On the base of laboratory data a set of approximating equations showing the change of electric properties in frequency domain ranging from 100-1000 MHz was obtained. These equations together with known from EM theory formulas for EM waves speed and attenuation were used for modeling and studying the process of radar waves propagation in a coal seam and reflection from the miners entrapped in collapsed tunnels. The modeling concept assumes that a radar transducer with dominating frequency MHz will be lowered through a vertical or inclined rescue borehole to the depth of coal seam with mining opening. It is assumed that in not collapsed part of tunnel only miner is present. Thus on the path of radar wave from transducer to a  human we have only one geological interface reflecting the radar | 1. We thank the reviewer carefully checked on this part. The title has been modified to Thank you very much for your detailed suggestions and comments on the manuscript. In particular, the reviewers' general remarks can be directly used as a new abstract of the paper. We have carefully revised the manuscript based on the comments. For example, Figures 1 and 2 have been removed from the paper. We have checked and corrected the words, descriptions, spelling and grammar in the draft in detail. |

| signals (coal-air) and one connected with human body. The human reflector can be located in various distances from the tunnel face. This factor was included in analyzed geometrical model. On the base of modeling results the authors of the paper conclude that a reliable human origin reflected radar signal can be measured when a distance between human and transducer do not exceed 5 m.  Although the concept and research data presented in the manuscript are very interesting and valuable scientifically it needs a thorough revision. There are a lot of deficiencies. Starting from the title, the next parts of manuscript (abstract, introduction and conclusions) in my opinion should be completely re written (my general remarks can be used as a new abstract in the paper). The rest of them need to be corrected. I suggest to remove figures 1 and 2 from the paper. They do not bring nothing and are detached from the content of the paper. Some other figures also need corrections. I recommend to send the manuscript back to authors for major revision. My detailed remarks are included in a word processor file, attached to the revision. I hope that they help the authors to correct the  manuscript |  |
| --- | --- |
