# Supplementary material for: Propagation of ground penetrating radar waves in Chinese coals
Source: PLoS One. 2020 May 21;15(5):e0233434. doi: 10.1371/journal.pone.0233434 (PMC7241710; doi:10.1371/journal.pone.0233434)
Supplement: Attachment — Submitted filename: Response to reviewers.docx [file pone.0233434.s008.docx]

**Reply to Reviewers’ Comments**

**Journal:** PLOS ONE

**Manuscript ID:** PONE-D-19-29325R1

Title: “Propagation of Ground Penetrating Radar Waves in Chinese Coals”

**Authors:** Duo Zhang, Rui Tang, Hu Wen, Shixing Fan

| **Editor** | |
| --- | --- |
| **Comments to the Author** | **Response** |
| Thank you for submitting your manuscript to PLOS ONE. After careful consideration, we feel that it has merit but does not fully meet PLOS ONE’s publication criteria as it currently stands. Therefore, we invite you to submit a revised version of the manuscript that addresses the points raised during the review process. We would appreciate receiving your revised manuscript by Mar 06 2020 11:59PM. Two reports have been collected for your submission from the same reviewers of the first round. Both the reports are generally positive, but one of the report still recommends major improvements. | We thank the anonymous reviewers for taking precious time to review our manuscript again. We appreciate the reviewers giving us valuable comments that are helpful to improve our manuscript. Here we attempt to reply where and how we revised our manuscript in the light of reviewers’ comments. Revised contents of our replies are marked in red font. |

| **Reviewer #1** | |
| --- | --- |
| **Comments to the Author** | **Response** |
| My concern on how to differentiate the radar reflections from many other reflections from inhomogeneities of the strata, underground equipment and objects has not been properly addressed. I fully understand that human is different from strata and equipment. But there are so many situations that you can observe similar reflections to those from human bodies. For example, will you be able to tell the difference of human reflections from the waterbody reflections in the strata? This is related to multi-solutions of geophysical problems – the same observation can be caused by many different situations. In addition, I have not been convinced by the authors’ response that their prototype radar system with a gyro can tell the direction of received radar waves, unless their radar system is a directional system. Can you provide an example of the testing results for trapped humans using your prototype radar system. Otherwise, there is a 360 degrees ambiguity. If you want to use the radar method for detection of the trapped miners in disasters, you have to address and discuss these issues.  I don’t think your paper has been properly edited by an academic editing people as there are many Chinglish expressions remained – some of them are in the attached marked manuscript. It is interesting to see that you just copied and pasted the reviewer’s comments as your abstract without changing a word. | Thanks for the reviewer’s comments. We can distinguish the return wave is reflected by the human or the formation water. There are two reasons for this. The first is that the electrical parameters of water are significantly different from the electrical parameters of the human body. The second is that the human's reflected waves are loaded with regular heartbeat signals.  As the reviewer said, our radar system is a directional system. As shown in Figure 1, the radar structure includes the transmit and receive window. The size of the window is 70 mm × 50 mm. Experts from the China National Key R & D Project team tested our radar prototype at Jining No. 2 Coal Mine. One of the test scenarios is shown in Figure 2, and the test results are shown in Figure 3.  The article has been edited by academic editing with the native language. We have provided the certificate of english editing in the previous reply. If required, we can invite academic editors to make it more standard. After reading the reviewers' comments, we all think that it is very perfect. It is more suitable as an abstract for a dissertation. Therefore, we have completely used the reviewer's comments as a new abstract.    Fig. 1 Schematic of radar structure  1: Battery; 2: Control module of power; 3: Data transmission module;  4: CPU; 5: Data acquisition module; 6: Receiving antenna; 7: Transmitting antenna; 8: Gyro; 9: Transmission line; 10: Computer; 11: Transmit and receive window.    Fig. 2 Test scenarios    Fig. 3 Test results displayed by the terminal (5.04 m ≈ 5.00 m) |

| **Reviewer #2** | |
| --- | --- |
| **Comments to the Author** | **Response** |
| The manuscript has been deeply improved and all my suggestions have been considered. Nevertheless, I've noticed some minor errors or "bugs" which have to be removed before publication. I attach a revised manuscript indicating noticed "bugs". After removal of them the manuscript can be accepted for publishing. | 1. We thank the reviewer carefully checked on this part. Thank you very much for your detailed suggestions and comments on the manuscript. We have carefully revised the manuscript based on the comments. Thank you again for your meaningful suggestions and comments. |
