# Supplementary material for: Propagation of ground penetrating radar waves in Chinese coals
Source: PLoS One. 2020 May 21;15(5):e0233434. doi: 10.1371/journal.pone.0233434 (PMC7241710; doi:10.1371/journal.pone.0233434)
Supplement: Attachment — Submitted filename: Response to reviewers.docx [file pone.0233434.s010.docx]

**Reply to Reviewers’ Comments**

**Journal:** PLOS ONE

**Manuscript ID:** PONE-D-19-29325R2

Title: “Propagation of Ground Penetrating Radar Waves in Chinese Coals”

**Authors:** Duo Zhang, Rui Tang, Hu Wen, Shixing Fan

| **Editor** | | | |
| --- | --- | --- | --- |
| **Comments to the Author** | **Response** | | |
| Thank you for submitting your manuscript to PLOS ONE. After careful consideration, we feel that it has merit but does not fully meet PLOS ONE’s publication criteria as it currently stands. Therefore, we invite you to submit a revised version of the manuscript that addresses the points raised during the review process. We would appreciate receiving your revised manuscript by Mar 06 2020 11:59PM. Two reports have been collected for your submission from the same reviewers of the first round. Both the reports are generally positive, but one of the report still recommends major improvements. | We thank the reviewers for taking precious time to review our manuscript again. We appreciate the reviewers giving us valuable comments that are helpful to improve our manuscript. Here we attempt to reply where and how we revised our manuscript in the light of reviewers’ comments. Revised contents of our replies are marked in red font. | | |
| **Reviewer #1** | | |  |
| **Comments to the Author** | | **Response** |  |
| 1. We can distinguish the return wave is reflected by the human or the formation water. There are two reasons for this. The first is that the electrical parameters of water are significantly different from the electrical parameters of the human body. The second is that the human's reflected waves are loaded with regular heartbeat signals.  Theoretically what you said is correct. However, in practice, how do you differentiate the human reflection from the formation water body reflection from recorded radargrams? Please provide your method in the paper. Can the radar record show or detect human’s heartbeats? What is the resolution of the GPR system? Can you please provide any evidence or references on how your GPR can detect heartbeating? Please write your paper more scientifically, not with statements without support.  2. As the reviewer said, our radar system is a directional system. As shown in Figure 1, the radar structure includes the transmit and receive window. The size of the window is 70 mm × 50 mm. Experts from the China National Key R & D Project team tested our radar prototype at Jining No. 2 Coal Mine. One of the test scenarios is shown in Figure 2, and the test results are shown in Figure 3.  You only provide numerical examples which prove nothing as any different objects can cause radar reflections theoretically. Your Figure 3 in your reply also shows nothing – why are there no actual radar data presented to support your conclusion? How good is the directionality of your radar system? What is the radiation pattern? Why don’t you include a real data example in this paper for human detection or event to see the heartbeating?  3. The article has been edited by academic editing with the native language. We have provided the certificate of english editing in the previous reply. If required, we can invite academic editors to make it more standard. After reading the reviewers' comments, we all think that it is very perfect. It is more suitable as an abstract for a dissertation. Therefore, we have completely used the reviewer's comments as a new abstract.  I have made some suggestions in the last review but you totally ignored my suggestions and believe that your English is fine! You may pay some attention to the following comments/suggestions (I am sure there are more in the paper):  1. Line 29: Prefer to change “the authors of the paper conclude” to “, it is concluded”. The reviewer’s writing is ok, but I don’t think the authors of the paper should use similar expression.  2. Line 31: Not quite sure what exact you mean by “do not exceed 5-8 m”! Do you mean it should not exceed 5m or you really mean it should not exceed 8m. Or you mean the distances between 5m and 8m are ok. How did you derive these number? Or is this because you only modelled the data up to 8m?  3. Line 43: I am not sure you have used “particularly significant” and “major” correctly for these events – please check your original Chinese meaning with the corresponding English.  4. Line 67/8: “Fan and Jia et al.” -> “Fan et al.”  5. Line 69: “Fan and Chang et al.” -> “Fan et al.”  6. Line 82: “Peng” -> “Peng et al.”  7. Line 85: “Xu” -> “Xu et al.”  8. Line 88: “Emslie et al.” -> “Emslie and Lagace”  9. Line 94: “Luo and Stolarczyk et al.” -> “Luo et al.”  10. Line 106: “Dielectric properties measurement” ->”Dielectric property measurement”  11. Line 108-110: Change “in” to “from”.  12. Line 130: “followed similar regularities” -> “follow a similar trend”. Using “regularity” is very Chinese!  13. Line 139: “larger” -> “higher”. The constant is “higher” not “larger”.  14. Line 160: “large” -> “high”. We normally say the attenuation is high or low not large or small in English!  15. Line 184, 188, 191: “large”->”high”; “small” ->”low”. | | 1.Thanks for the reviewer’s comments. The beating of the heart can cause micro-undulation vibrations in the chest cavity at approximately periodic frequencies. The purpose of human body detection can be achieved form identifying the frequency of these micro-motion signals. Obstacles such as formation water bodies have no periodic frequency characteristics. But it still causes interference to radar waves. Therefore, it is necessary to effectively process the radar echoes in order to extract and identify signals containing human micro-motion information features. The method of identifying the characteristics of the micro-motion signal of radar echo information is as follows. The first is the preprocessing of the echo signal. It mainly includes DC offset removal, background removal, time-varying gain, and mean denoising of the echo signal. The second is the selection of sliding time window function and energy integration. The third is to use wavelet transform to process the energy integration results. The number of radar signal sampling points is 4096, and the scanning speed is 16Hz. In particular, radar can be used to obtain heartbeat and respiration signals, which has been employed in many areas. Such as: “Xiaolin Liang, Hao Zhang, Shengbo Ye, et al. Improved denoising method for through-wall vital sign detection using UWB impulse radar, Digital Signal Processing 74(2018) 72–93”, “Minhhuy Le. Heart rate extraction based on eigenvalues using UWB impulse radar remote sensing, Sensors and Actuators A 303 (2020) 111689”, “F. JalaliBidgoli, S. Moghadami, S. Ardalan, A compact portable microwave life-detection device for finding survivors, IEEE Embed. Syst. Lett. 8(1) (2016) 10–13”.  2. We thank the reviewer for this valuable comment. He is correct that any different object can cause radar reflections in theory. However, the slight fluctuation of the chest cavity caused by heartbeats are periodic and can be identified from radar echoes through a series of algorithm processing. In this article, we focus on establishing an approximate equation of radar wave propagation in coal and try to solve it. So there are no examples of measured data in this article. As a response to this comment, we add a set of actual radar data in the attachment. The schematic diagram of the experiment is shown in Figure 1. In future works, we will research on measured data and try to propose micro-vibration recognition algorithms for the life information. The detection direction of the radar system is shown in Figure 2. The angle between the detection direction and the horizontal direction is smaller than 120 ^o^C, while the angle between the detection direction and the vertical direction is smaller than 90 ^o^C. According to the detection angle, this detection scheme can be realised by adjusting direction of the radar window.    Fig. 1 Test scenarios     （a）horizontal direction （b）vertical direction  Fig. 2 detection angle of the radar system  3.We thank the reviewer’s comments. We found a lot of problems about English expression. We improved it according to the suggestion. The article was edited again by academic editing with the native language. Thank you again for your meaningful suggestions and comments. |  |

| **Reviewer #2** | |
| --- | --- |
| **Comments to the Author** | **Response** |
| I would response in point 5 "yes" but I have noticed some, following typographical errors in the manuscript:  line 102 ........ radar waves in coal "are" constructed (should be "is" constructed),  line 132 ........ largest "one" (should be plural "ones"),  line 216 ......... is relatively (should be "relative").  Figure captions:  Fig.5 variations of tesed coals (should be "tested"),  Fig.9 I - schematic "of" illustration of disaster (should be schematic illustration of disaster),  Fig.9 II - Schematic "of simplified" of physical model (should be schematic physical model),  Fig.11 - Relationship between (or a plot) between............. Dual expression. Should be only one eg. Relationship between amplitudes........ (remove "or a plot"). | 1. We thank the reviewer carefully checked on this part. Thank you very much for your detailed suggestions and comments on the manuscript. We have carefully revised the manuscript based on the comments. Thank you again for your meaningful suggestions and comments. |
